# Clinical manifestations of respiratory syncytial virus infection and the risk of wheezing and recurrent wheezing illness: a systematic review and meta-analysis

**DOI:** 10.1007/s12519-023-00743-5

**Published:** 2023-08-02

**Authors:** Ming-Yue Jiang, Yu-Ping Duan, Xun-Liang Tong, Qiang-Ru Huang, Meng-Meng Jia, Wei-Zhong Yang, Lu-Zhao Feng

**Affiliations:** 1 School of Population Medicine and Public Health, Chinese Academy of Medical Sciences and Peking Union Medical College, #9 Dong Dan San Tiao, Dongcheng District, Beijing, 100730 China; 2 Department of pulmonary and critical care medicine, Beijing Hospital/National Gerontology Center/Institute of Gerontology, Chinese Academy of Medical Sciences, Beijing, 100730 China; 3 State Key Laboratory of Respiratory Health and Multimorbidity, Chinese Academy of Medical Sciences and Peking Union Medical College, Beijing, China; 4 Key Laboratory of Pathogen Infection Prevention and Control (Peking Union Medical College), Ministry of Education, Beijing, China

**Keywords:** Clinical manifestations infants infection, Recurrent wheezing Respiratory syncytial virus infection, Wheezing

## Abstract

**Background:**

Respiratory syncytial virus (RSV) infection in infants is a global health priority. We aimed to investigate the common manifestations of RSV infection by age group and human development index (HDI) level and to assess its association with the development of wheezing and recurrent wheezing illness.

**Methods:**

We searched the literature published between January 1, 2010 and June 2, 2022 in seven databases. Outcomes included common manifestations and long-term respiratory outcomes of RSV infection in children. Random- and fixed-effect models were used to estimate the effect size and their 95% confidence intervals. Subgroup analysis was conducted by age and HDI levels. This review was registered in PROSPERO (CRD42022379401).

**Results:**

The meta-analysis included 47 studies. The top five manifestations were cough (92%), nasal congestion (58%), rhinorrhea (53%), shortness of breath (50%), and dyspnea (47%). The clinical symptoms were most severe in infants. In our analysis, compared to very high and high HDI countries, fewer studies in medium HDI countries reported related manifestations, and no study in low HDI countries reported that. The RSV-infected infants were more likely to develop wheezing than the non-infected infants [odds ratio (OR), 3.12; 95% CI, 2.59–3.76] and had a higher risk of developing wheezing illnesses after recovery (OR, 2.60; 95% CI, 2.51–2.70).

**Conclusions:**

Cough and shortness of breath are common manifestations of RSV infection. More attention should be given to infants and areas with low HDI levels. The current findings confirm an association between RSV infection and wheezing or recurrent wheezing illness.

**Supplementary Information:**

The online version contains supplementary material available at 10.1007/s12519-023-00743-5.

## Introduction

Respiratory syncytial virus (RSV) is one of the most frequently detected pathogens in the lower respiratory tract of infected patients aged ≤ 5 years [1]; most children are infected by RSV before the age of 2 years [2, 3]. RSV can cause acute and severe acute respiratory infection and severe acute respiratory syndrome [4, 5]. RSV can be detected in patients with influenza-like illness (ILI) and those hospitalized with community-acquired pneumonia [6, 7]. The absence of RSV-specific clinical manifestations in infected children poses an obstacle to early diagnosis and timely treatment. A study conducted in the United States identified 1554 RSV-associated hospitalizations in children aged < 2 years, of whom, 27% were admitted to an intensive care unit, 6% needed mechanical ventilation [8]. Another population-based cohort study, which included 310,423 patients aged 0–17 years admitted to hospitals with RSV in Denmark, found that 54 children with RSV received mechanical ventilation in 2021–22, compared with 15–28 annually in the 2016–17 to 2019–20 RSV seasons [9]. A meta-analysis during 2010 and 2015 showed that the most common clinical manifestations among patients with RSV in China were cough (93.9%), expectoration (66.3%), wheezing (65.7%), fever (43.0%), rhinorrhea (42.7%), cyanosis (38.9%), shortness of breath (32.2%), diarrhea (18.8%), and dyspnea (12.8%) [10]. However, few meta-analyses reported a synthesized rate for clinical manifestations and severity among RSV-infected patients aged ≤ 5 years worldwide. Therefore, country- or region-specific studies synthesizing age-stratified clinical manifestations are needed. Furthermore, in the postcoronavirus disease 2019 era, there has been decreased seasonal exposure and increased susceptibility to RSV among children, which might increase the severity of these manifestations [11].

Moreover, RSV infection is linked to the development of wheezing and asthma and might be a risk factor for a subsequent wheezing illness after recovery. Children aged < 1 year infected with RSV have an increased risk of wheezing and asthma during the following 12 years [12]. RSV infection in early life might also aggravate subsequent asthma and wheezing illnesses. The risks of hospitalization for asthma and use of asthma medication were 3.3 and 1.7 times higher, respectively, in patients with RSV infection than in those without [13]. Studies have noted that the influence of genetic susceptibility, underlying health status, and co-infections with other viruses cannot be fully covered in observational studies, confounding the association between RSV infection and subsequent wheezing or asthma [14]. The World Health Organization documented the lack of evidence showing that RSV immunization measures effectively prevent wheezing or asthma [15]. This implies a lack of evidence to support a direct causality between RSV infection and recurrent wheezing illness. Therefore, more studies are needed to clarify this association.

This systematic review and meta-analysis aimed to explore the common clinical manifestations of RSV infection by age group and HDI level and to clarify the association between RSV infection and wheezing or recurrent wheezing illness.

## Methods

### Search strategy and selection criteria

**Fig. 1:**
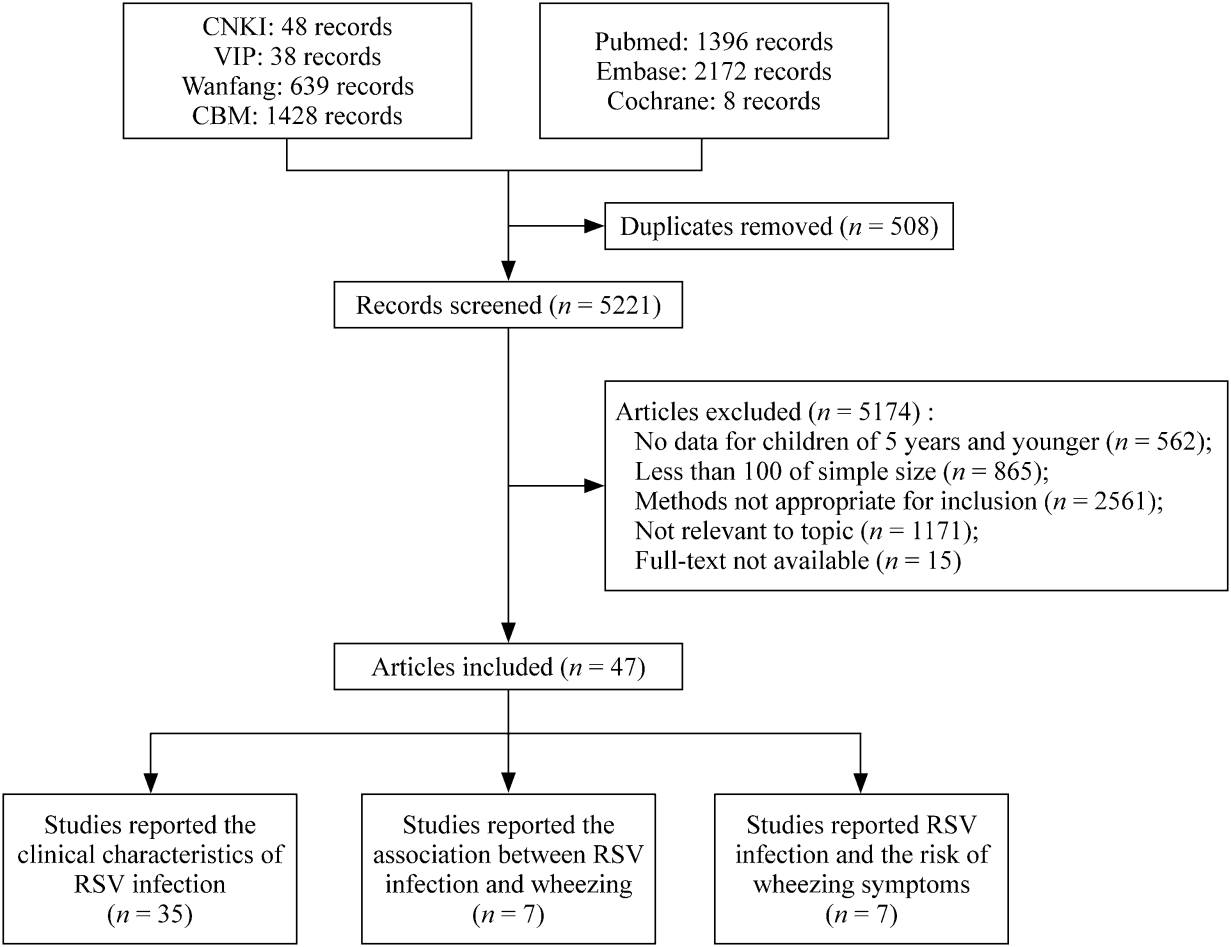
Flow diagram for the selection of studies. *RSV* respiratory syncytial virus, *CNKI* Chinese National Knowledge Infrastructure, *VIP* Chinese Scientific Journal Database, *CBM* Chinese Biomedical Literature Database, *Wanfang* Wanfang Database

We searched the published literature in seven databases—PubMed, Embase, The Cochrane Library, CNKI (Chinese National Knowledge Infrastructure), Wanfang (Wanfang Database), CBM (Chinese Biomedical Literature Database), and VIP (Chinese Scientific Journal Database)—using the following search terms: (Respiratory Syncytial Virus OR Respiratory Syncytial Virus Infection OR Human Respiratory Syncytial Virus OR Human orthopulmonary virus OR RSV OR HRSV) AND (Child OR Infant OR Pediatrics OR Toddler OR Baby OR Newborn OR Neonate OR kindergarten OR Preschool OR preschool child OR schoolchild OR school age OR Neonatology department OR Pediatric department OR nurser) AND (Incidence OR Infection OR Positive serum antibody OR Admission OR Hospitalization OR HLOS OR length of stay OR LOS OR Hospital stay) AND (Association OR Risk factor OR Prognosis OR SU = complication OR sequela) (Supplementary Table 1). All articles published between January 1, 2010, and June 2, 2022, were included. The search terms included Medical Subject Headings and “free text” terms (Fig. 1). The study was registered in the International Prospective Register of Systematic Reviews (PROSPERO; CRD42022379401) and is reported following the guidelines of the Preferred Reporting Items for Systematic Reviews and Meta-Analyses (PRISMA) [16].

The inclusion and exclusion criteria are described in Table 1 using the population, intervention or exposure, comparators, and outcomes format. Two independent reviewers screened the titles and/or abstracts after removing duplicates. A third reviewer was consulted when the two reviewers disagreed on a study’s assessment.

**Table 1:** Inclusion and exclusion criteria using the PICO format

Variables	Common clinical manifestations	Rehabilitation
Population characteristics	RSV-infected children aged five years and younger	
Intervention or exposure	RSV infection in the first five years of life	
Comparator (s)	^a^	RSV-negative children
Outcomes	Common clinical manifestations	Wheezing illness^b^
Study design	Cohort study, cross-sectional study, case–control study, case-cohort study, and nested case–control study	Case–control study, case-cohort study, and cohort study
Year published	January 1, 2010 to June 2, 2022	
Sample size	More than 100	
Exclusion	(1) Adults, the elderly, or children over the age of five; (2) unhealthy children, children with congenital heart disease or immunodeficiency disease, etc.; (3) reported composition ratio; (4) comparison of two RSV detection methods; (5) studies on languages other than Chinese and English; (6) animal research, repeated publications, letters, comments, case reports, case series, editorials, reviews, pure model studies, or model studies for trend estimation; (7) full text that was not available; and (8) only articles with the most comprehensive data were included in the repeated articles	

*PICO* population, intervention or exposure, comparators, and outcomes *RSV* respiratory syncytial virus

^a^To calculate a single proportion of each clinical manifestation, and no comparator variables for this section

^b^Because studies did not use consistent definitions for asthma and other wheezing illnesses, all variations of wheezing illness were combined into a single outcome

### Data extraction and quality assessment

Two investigator groups independently extracted the following data from eligible studies: first author; survey period; region or country; race; study design; data sources; case definitions; sample collection or detection method; sample size; age groups; sex; quality score; and outcomes, including common clinical manifestations and long-term respiratory outcomes of RSV infection (wheezing illness during RSV infection or after recovery; Supplementary tables 2–4). Disagreements were resolved by an independent senior reviewer.

The quality of the included studies was assessed using the Newcastle–Ottawa Scale for cohort studies [17]. A study with a score < 5 was considered low quality. The Agency for Healthcare Research and Quality evaluated the risk of bias for cross-sectional studies [18]. Studies were classified as high-, moderate-, or low-quality studies based on their total scores (8–11, 4–7, and 0–3 points, respectively; Supplementary Tables 5 and 6).

### Data synthesis and analysis

We used Stata SE 17.0 (Stata Corp., College Station, TX, USA) to analyze the extracted data. The common clinical manifestations were assessed using pooled estimates of one-group meta-analyses. The ratio-based effect was estimated in children with and without RSV infection who developed wheezing.

Data from each study were weighted and analyzed separately, and their pooled rates or ratio-based effects were estimated. The pooled estimate was calculated using the Mantel–Haenszel method with a random- or fixed-effects model. The statistical heterogeneity of the pooled data was assessed using *I*^2^ and Cochrane’s tests. When significant heterogeneity was observed (*P* < 0.05, *I*^2^ ≥ 50%), a random-effects model was applied [19].

Individual, summary pooled rate, ratio-based effects and their 95% confidence intervals (CIs) were visualized using forest plots. A funnel plot and Egger’s test were used to assess publication bias and detect symmetry. Influence analysis based on the “leave-one-out” method was performed by repeating the meta-analysis K-1 times, each time leaving one study out to evaluate its effect on the overall estimate.

## Results

### Search outcomes

Following the systematic search of the seven databases with advanced search criteria, 5729 records were retrieved. Of these, 508 were excluded as duplicates, and 5174 were excluded after reviewing their title and abstract. The remaining 47 records were included in this systematic review. Of the cross-sectional studies, 22 were considered high quality, and 18 were considered moderate quality. None of the cohort studies were of low quality. The detailed study selection process following the PRISMA guidelines is shown in Fig. 1.

### Characteristics of the studies

Of the 47 studies, 35 reported clinical manifestations of RSV infection, covering 24,822,974 participants. Overall, 7 studies, with 3035 participants, found an association between RSV infection and wheezing illness, and 7 studies, with 228,301 participants, found an association between early childhood RSV infection and a subsequent development of recurrent wheezing illness. There were 23, 20, and 4 studies that were conducted in very high-, high-, and medium-HDI countries, respectively (Supplementary Tables 2–4).

### Meta-analysis outcomes

#### Clinical manifestations in patients with RSV infection

The pooled incidence of mild clinical manifestations was 51%, significantly higher than that of moderate (37%) and severe (7%) clinical manifestations (Table 2). Cough was the most common mild clinical manifestation (92%), followed by nasal congestion (58%), rhinorrhea (53%), and fever (41%). Vomiting and diarrhea as mild clinical manifestations had incidences of 22% and 20%, respectively. Among the moderate clinical manifestations, shortness of breath (50%) and dyspnea (47%) were the most frequently reported, followed by wheezing (39%), pneumonia (28%), and the need for oxygen supplementation (26%). The incidence of otitis media was relatively low (13%). Respiratory failure was the most common severe clinical manifestation (29 %), followed by sepsis (10%) and the need to be admitted to the intensive care unit (ICU, 9%). Only 2% of the patients required mechanical ventilation intervention.

**Table 2:** Common clinical manifestations of RSV-infected children

Outcomes	Effect (95% CI)	*I*^2^ (%)	*P* value	Number of included studies
Mild	0.51 (0.39–0.64)	100.0	< 0.001	14
Cough	0.92 (0.88–0.95)	97.9	< 0.001	10
Nasal congestion	0.58 (0.4–0.76)	98.6	< 0.001	5
Rhinorrhea	0.53 (0.05–1.00)	100.0	< 0.001	5
Fever	0.41 (0.34–0.49)	98.2	< 0.001	13
Vomiting	0.22 (0.11–0.32)	99.2	< 0.001	4
Diarrhea	0.20 (0.12–0.27)	98.1	< 0.001	6
Moderate	0.37 (0.30–0.45)	100.0	< 0.001	20
Shortness of breath	0.50 (0.31–0.69)	99.8	< 0.001	9
Dyspnea	0.47 (0.05–0.88)	99.8	< 0.001	4
Wheezing	0.39 (0.30–0.48)	99.6	< 0.001	16
Pneumonia	0.28 (− 0.06–0.62)	100.0	< 0.001	6
Oxygen supplementation	0.26 (− 0.01–0.54)	100.0	< 0.001	5
Otitis media	0.13 (0.12–0.15)	0.0	0.642	2
Severe	0.07 (0.07–0.08)	99.8	< 0.001	19
Respiratory failure	0.29 (0.16–0.41)	98.8	< 0.001	5
Sepsis	0.10 (− 0.03–0.23)	99.4	< 0.001	3
ICU admission	0.09 (0.07–0.10)	99.8	< 0.001	15
Mechanical ventilation	0.02 (0.02–0.03)	99.1	< 0.001	8

*RSV* respiratory syncytial virus, *CI* confidential interval, *ICU* intensive care unit

The participants were divided into the 0–1 and 0–2 years age groups. The pooled incidences of mild, moderate, and severe clinical manifestations in the 0–1 year age group were 55%, 50%, and 9%, respectively. RSV-associated clinical manifestations included cough (90%), shortness of breath (60%), oxygen supplementation requirement (54%), wheezing (42%), fever (40%), diarrhea (24%), the need for ICU admission (12%), and mechanical ventilation requirement (1%). The pooled incidences of mild, moderate, and severe clinical manifestations in the 0–2 years age group were 51%, 39%, and 6%, respectively. RSV-associated clinical manifestations included cough (93%), shortness of breath (57%), fever (45%), wheezing (34%), oxygen supplementation requirement (26%), diarrhea (18%), the need for ICU admission (7%), and mechanical ventilation requirement (6%) (Table 3 and Supplementary Fig. 1).

**Table 3:** Common clinical manifestations of RSV infected children in different age groups and HDI level countries

Subgroups	Outcomes	Effect (95% CI)	*I*^2^ (%)	*P* value	Number of included studies
**0–1 y**					
Mild		**0.55 (0.34––0.76)**	**99.7**	** < 0.001**	**5**
	Cough	0.9 (0.83–0.98)	96.6	< 0.001	4
	Fever	0.4 (0.22–0.59)	98.0	< 0.001	5
	Diarrhea	0.24 (0.2–0.29)	33.6	0.220	2
Moderate		**0.50 (0.32–0.68)**	**99.5**	** < 0.001**	**6**
	Shortness of breath	0.6 (0.29–0.9)	98.8	< 0.001	3
	Wheezing	0.42 (0.07–0.77)	99.6	< 0.001	4
	Oxygen supplementation	0.54 (0.53–0.55)	N/A	N/A	1
Severe		**0.09 (0.07–0.12)**	**99.9**	** < 0.001**	**6**
	ICU admission	0.12 (0.09–0.16)	99.8	< 0.001	6
	Mechanical ventilation	0.01 (0.00–0.02)	94.5	< 0.001	2
0–2 y					
Mild		**0.51 (0.28–0.75)**	**99.9**	** < 0.001**	**5**
	Cough	0.93 (0.88–0.99)	98.0	< 0.001	4
	Fever	0.45 (0.31–0.58)	98.7	< 0.001	5
	Diarrhea	0.18 (0.08–0.27)	98.5	< 0.001	4
Moderate		**0.39 (0.26–0.51)**	**99.6**	** < 0.001**	**8**
	Shortness of breath	0.57 (0.4–0.74)	98.9	< 0.001	3
	Wheezing	0.34 (0.19–0.5)	99.5	< 0.001	8
	Oxygen supplementation	0.26 (0.11–0.42)	98.7	< 0.001	3
Severe		**0.06 (0.05–0.08)**	**99.6**	** < 0.001**	**6**
	ICU admission	0.07 (0.05–0.08)	99.7	< 0.001	6
	Mechanical ventilation	0.06 (0.03–0.08)	93.6	< 0.001	3
Very high HDI^a^					
Mild		**0.56 (0.33–0.80)**	**99.9**	** < 0.001**	**2**
	Cough	0.9 (0.8–1.00)	98.4	< 0.001	2
	Rhinorrhea	0.61 (0.37–0.86)	99.0	< 0.001	2
	Fever	0.59 (0.57–0.62)	0.0	0.516	2
	Diarrhea	0.15 (0.14–0.17)	0.0	0.928	2
Moderate		**0.25 (0.12–0.38)**	**100**	** < 0.001**	**8**
	Shortness of breath	0.57 (0.54–0.59)	N/A	N/A	1
	Wheezing	0.27 (0.18–0.35)	99.0	< 0.001	6
	Pneumonia	0.03 (–0.02 to 0.09)	98.1	< 0.001	2
	Oxygen supplementation	0.27 (–0.26 to 0.80)	100.0	< 0.001	2
Severe		**0.06 (0.06–0.07)**	**99.8**	** < 0.001**	**12**
	ICU admission	0.10 (0.08–0.12)	99.9	< 0.001	11
	Mechanical ventilation	0.02 (0.02–0.03)	99.0	< 0.001	6
High HDI^a^					
Mild		**0.49 (0.30–0.68)**	**100.0**	** < 0.001**	**11**
	Cough	0.93 (0.89–0.96)	97.5	< 0.001	7
	Rhinorrhea	0.21 (–0.15 to 0.57)	99.9	< 0.001	2
	Fever	0.33 (0.27–0.40)	96.4	< 0.001	9
	Diarrhea	0.22 (0.06–0.38)	98.5	< 0.001	4
Moderate		**0.47 (0.32–0.61)**	**99.9**	** < 0.001**	**10**
	Shortness of breath	0.54 (0.244–0.83)	99.8	< 0.001	6
	Wheezing	0.49 (0.33–0.66)	99.5	< 0.001	8
	Pneumonia	0.56 (–0.22 to 1.34)	100.0	< 0.001	2
	Oxygen supplementation	0.26 (0.15–0.37)	98.1	< 0.001	4
Severe		**0.05 (0.03–0.07)**	**93.4**	** < 0.001**	**5**
	ICU admission	0.06 (0.04–0.08)	88.5	< 0.001	4
	Mechanical ventilation	0.03 (0.01–0.05)	87.7	0.004	2
Medium HDI^a^					
Mild		**0.76 (0.58–0.94)**	**99.1**	** < 0.001**	**3**
	Cough	0.87 (0.83–0.91)	N/A	N/A	1
	Rhinorrhea	0.98 (0.97–0.99)	N/A	N/A	1
	Fever	0.59 (0.51–0.67)	68.10	0.077	2
Moderate		**0.31 (0.16–0.46)**	**99.0**	** < 0.001**	**4**
	Shortness of breath	0.34 (0.20–0.48)	93.60	< 0.001	2
	Wheezing	0.34 (–0.23 to 0.91)	99.70	< 0.001	2
	Pneumonia	0.25 (0.10–0.40)	97.60	< 0.001	2

The bold values in the table represent the synthetic percentage of mild, moderate, and severe clinical symptoms in each subgroup

*RSV* respiratory syncytial virus *CI* confidential interval *N/A* not analyzed *ICU* intensive care unit *HDI* human development index

^**a**^According to the classification of the United Nations Development Program in the Human Development Report 1990, we classified the countries included in our study into very high HDI countries (the United States of America, Finland, Sweden, Japan, Canada, Netherlands, Mexico, France, Argentina), high HDI countries (China, Jordan, India), and mediun HDI countries (Nepal, Kenya, Guatemala)

The included studies were divided into HDI levels of very high, high, medium, and low. In countries with very high HDI, the pooled incidences of mild, moderate, and severe clinical manifestations were 56%, 25%, and 6%, respectively. The clinical manifestations included cough (90%), rhinorrhea (61%), fever (59%), shortness of breath (57%), oxygen supplementation requirement (27%), wheezing (27%), diarrhea (15%), the need for ICU admission (10%), pneumonia (3%), and mechanical ventilation requirement (2%). In high HDI countries, the pooled incidences of mild, moderate, and severe clinical manifestations were 49%, 47%, and 5%, respectively. The clinical manifestations included cough (93%), pneumonia (56%), shortness of breath (54%), wheezing (49%), fever (33%), oxygen supplementation requirement (26%), diarrhea (22%), rhinorrhea (21%), the need for ICU admission (6%), and mechanical ventilation requirement (3%). Few studies in medium HDI countries reported the incidence of rhinorrhea (98%), cough (87%), fever (59%), wheezing (34%), shortness of breath (34%), and pneumonia (25%). In medium HDI countries, the pooled incidences of mild and moderate clinical manifestations were 76% and 31%, respectively. There were no data for low HDI countries (Table 3 and Supplementary Fig. 2). A total of 24 studies reported the length of hospital stay, and the average, weighted by sample size, was 4.5 (range, 2.0–13.5) days (Supplementary Table 7).

#### Association between RSV infection and wheezing or recurrent wheeze illness

**Fig. 2:**
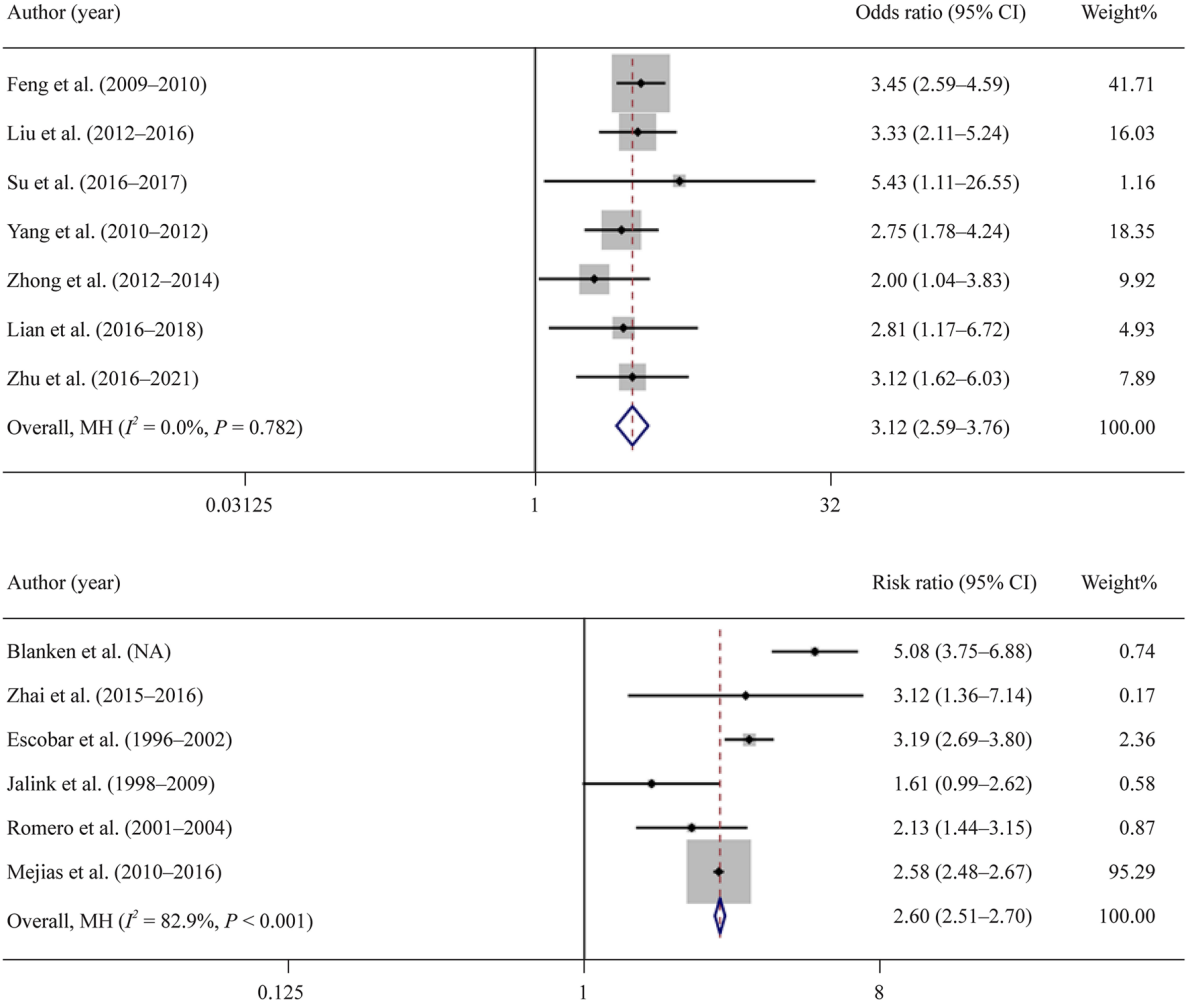
The associations of RSV-positive and RSV-negative patients with the development of wheezing and recurrent wheezing illness. a Overall impact of RSV infection on the occurrence of wheezing when compared with noninfected children (weights are from mantel-haenszel model); b overall impact of RSV infection in early childhood on subsequent development of recurrent wheeze illness when compared with noninfected children (Weights are from mantel-haenszel model). *RSV* respiratory syncytial virus *CI* Confidence interval, *MH *mantel-haenszel model

Seven studies investigated the association between RSV infection and wheezing. The RSV-positive group was more likely to develop wheezing than the control group [odds ratio OR, 3.12; 95% CI, 2.59–3.76)]. The forest plot demonstrated homogeneity among studies (*I*^2^ = 0.0%, *P* = 0.782; Fig. 2a).

Seven studies investigated the association between early childhood RSV infection and subsequent development of a recurrent wheezing illness. The RSV-positive group was more likely to develop recurrent wheezing illness than the control group (OR, 2.60; 95% CI, 2.51–2.70). There was substantial heterogeneity among these studies (*I*^2^ = 82.8%, *P* < 0.001; Fig. 2b).

#### Publication bias and sensitivity analysis

Potential publication bias was determined only for outcomes in at least 10 studies. Egger’s test found no publication bias for the incidence of wheezing (*t* = 1.05, *P* = 0.314) and fever (*t* = 0.11, *P* = 0.915) outcomes. However, a significant publication bias was found for the incidence of ICU admission (*t* = 4.12, *P* = 0.001) and cough (*t* = 2.63, *P* = 0.030) outcomes (Supplementary Fig. 3). Influence analysis showed that no study had a significant effect on the overall estimate of the meta-analysis (Supplementary Fig. 4), except for the study by Mejias et al., which reported an association between RSV infection and subsequent development of recurrent wheezing illness [20].

## Discussion

RSV infection could pose an enormous health threat to children, resulting in severe clinical outcomes and poor prognosis. This meta-analysis included 47 studies that reported on common clinical manifestations of RSV infection and the relationship between RSV infection and wheezing or recurrent wheezing illness. The results demonstrated that the prevalence of mild clinical manifestations was significantly higher than that of moderate or severe clinical manifestations. Cough and shortness of breath were common symptoms of RSV infection, whereas fever was less common. Moderate and severe clinical manifestations were more likely to occur among children aged 0–1 year than among those aged 0–2 years, and sufficient treatment in relatively high HDI countries may reduce disease severity. RSV infection was associated with the development of wheezing or recurrent wheezing illness. Our study highlighted the primary clinical manifestations of RSV infection in young children to help clinicians diagnose and treat in a timely manner. Subsequent wheezing or recurrent wheezing illness in children with RSV should be highlighted.

Only a few previous systematic reviews or meta-analyses have focused on common clinical manifestations of RSV infection. In our study, cough was the most common mild clinical symptom in children with RSV infection, and fever was less common, implying that fever might not be a sensitive indicator to identify patients with RSV infection by surveillance based on influenza-like illnesses. Among the moderate and severe clinical manifestations, the incidence of shortness of breath and dyspnea in our study was significantly higher than that of wheezing. However, Zhang et al. found a wheezing incidence of 65.7% among patients with RSV infection, whereas the incidences of shortness of breath and dyspnea were 32.2% and 12.8%, respectively [10]. Their study included all age groups in the population, which may have caused the slight difference from our study, suggesting that moderate and severe clinical manifestations might vary among age groups.

Meanwhile, the incidence of sepsis among hospitalized children with RSV infection less than 6 months of age and neonates (0–28 days), were reported to be 0.4% and 11.9% in the study of China [21, 22]. A hospital surveillance conducted by Halasa et al. found that the positive rate of RSV in children admitted to the hospital with suspected sepsis was 18% [23]. None of the above studies specifically reported the causative bacteria of superinfection. Other symptoms of RSV infection included digestive symptoms such as diarrhea and vomiting (pooled rate: vomiting: 22%; diarrhea: 20%). Impaired liver function (0.9%) [21], dehydration (8%), cyanosis (6%), and seizures (2%) have also been reported [8]. Furthermore, the in-hospital mortality among the included studies ranged from 1.7 to 2.0% [10, 24, 25].

Further age-based subgroup analysis in this study showed that the incidence of mild clinical manifestations such as cough, fever, and diarrhea was similar between the 0–1 year and 0–2 years age groups. Conversely, the incidences of moderate (50% vs. 39%) and severe (9% vs. 6%) clinical manifestations were higher in the 0–1 year age group than in the 0–2 years age group. This was especially apparent in those requiring oxygen supplementation and ICU admission, whose respective incidence in the 0–1 year age group (54% and 12%, respectively) was nearly twice that in the 0–2 years age group (26% and 7%, respectively). This finding suggested that RSV infection might show more severe clinical manifestations in younger children. More studies should be conducted to provide evidence and guidance for implementing age-adjusted clinical treatments.

Many studies have found that the incidence, hospitalization rate, and mortality of patients with RSV infection differed among economic levels [26–28]. The incidence of mild manifestations was higher than that of moderate manifestations in very high HDI countries (56% *vs.* 25%). However, these rates were almost equal in high HDI countries (49% *vs.* 47%). Early diagnosis and sufficient medical resource for treatment with RSV-infected children in very high HDI countries may result in a reduced clinical severity. This explanation is supported by the fact that although the RSV-related hospitalization rate was higher in developed countries, the case fatality and mortality rates were higher in developing countries [29]. There were few eligible studies in medium HDI countries, and no studies from low HDI countries. This may imply a lack of diagnostic awareness in those countries, leading to the misdiagnosis of RSV infection, an underestimation of disease burden, and a delay in the administration of optimal treatment. More data should be collected from these countries for future analysis.

Wheezing in infants can be divided into three main categories: transient, nonallergic persistent, and allergic wheezing, of which transient wheezing is the most common. Allergic wheezing is presumably related to some genetic factors [30], whereas nonallergic persistent wheezing is mostly caused by infection [31]. It is widely assumed that viral infection is linked to wheezing in children, with RSV infection being the most common. The detection rate of RSV in children with wheezing illnesses in Suzhou, China, was 22% and reached 42.4% at the peak of the RSV epidemic [32]. Another study found that RSV accounted for 67.4% of the detected viruses in children with wheezing illness, ranking first among the detected pathogens [33]. RSV infection was also associated with recurrent wheezing after infant bronchiolitis [34]. The mechanism through which RSV infection leads to wheezing and asthma remains unclear. The generally accepted theory suggests that RSV can invade respiratory epithelial cells and form syncytia bodies that can infect more cells after shedding. Shedding of infected cells and syncytia bodies might block the airway, expose the basement membrane of the respiratory tract, and lead to the release of neurogenic factors that can cause bronchial smooth muscle spasm and airway hyperresponsiveness [35]. Furthermore, RSV infection always occurs during the airway development window in children, leading to submucosal neural network remodeling and the release of inflammatory and immune response cellular effectors. These can also cause airway hyperresponsiveness [36].

However, owing to the complex relationship between the highly variable outcomes of RSV infection and host genetic diversity, observational studies could not confirm that RSV infection in early life is a direct cause of wheezing or recurrent wheezing illness after recovery [37]. Brunwasser et al. adjusted for genetic susceptibility factors through a modeling study and found that the risk of recurrent wheezing was 2.45 times higher in children with RSV infection than in children without RSV infection, slightly lower than that in our study [14]. These findings reveal that the risk of RSV infection for subsequent wheezing illness might be overestimated in observational studies that do not adjust for potential confounders. The case and control groups cannot be fully matched for genetic susceptibility and basic health conditions, and other factors affecting the association between RSV infection and wheezing illness might exist. Makrinioti et al. conducted a meta-analysis to investigate the relationship between RSV-related bronchitis and subsequent recurrent wheezing illness [38]. Their results showed that the risk of subsequent wheezing illness and asthma in children with RSV-positive bronchitis was similar to that in children with RSV-negative bronchitis. However, children with RSV-positive bronchitis had a significantly higher risk of developing subsequent wheezing illness and asthma than healthy children [38]. They also pointed out that the rhinovirus infection group had a higher risk of developing subsequent asthma than the RSV infection group, suggesting that infection by other pathogens might also be a confounding factor affecting the correlation between RSV infection and the occurrence of wheezing symptoms. However, most studies have confirmed a positive association between RSV infection and the occurrence of wheezing illnesses, suggesting that these unmatchable confounders might mainly affect the intensity. Therefore, more attention should be given to wheezing-related complications in children with RSV infection. It is important to reduce their exposure to factors that might lead to wheezing illnesses, such as allergens and specific pathogens. Early prevention and timely treatment could reduce the development and severity of wheezing illnesses and improve patients’ quality of life. More researches, such as studies on twins, is needed to match confounding factors and obtain reliable conclusions.

This study had some limitations. First, a limited number of original studies met our inclusion criteria, especially on some clinical manifestations, resulting in large confidence intervals that limited the precision of our findings. Nevertheless, we combined the sample sizes of all the studies and tried to avoid small sample size effects. Second, different methods were used in the original studies to assess the outcomes, including case ascertainment of RSV infection and assessment of wheezing illness. These factors possibly introduced heterogeneity in our synthetical results, which cannot be adjusted for. Third, a subanalysis could not be performed on all clinical manifestations due to the limited number of studies included. In addition, few studies have reported clinical manifestations in children aged 2–5 years or 1–2 years; therefore, we could only divide the groups into age groups of 0–1 year and 0–2 years.

Substantial heterogeneity among studies in the association between RSV infection and recurrent wheezing was noted. Some studies included term infants [20, 39], whereas others included moderately premature infants [40–42]. Baseline characteristics such as gene susceptibility to wheezing illnesses, history of infection with other pathogens, and general health status could not be controlled and differentiated among studies, resulting in increased heterogeneity. Nevertheless, all seven included studies showed a positive association between RSV infection and recurrent wheezing illness. Consequently, RSV infection should still be considered a risk factor for subsequent wheezing illnesses.

In conclusion, respiratory tract infection symptoms, such as cough and shortness of breath, are the most common clinical manifestations of RSV infection, and the incidence of fever is low. This suggests that surveillance based on the influenza-like illnesses, which takes fever as the surveillance symptom, might not be a suitable surveillance strategy for identifying patients with RSV infection. More attention should be given to young children aged 0–1 year and to areas with low development levels. RSV infection is associated with wheezing and recurrent wheezing illnesses. Early diagnosis and treatment of RSV infection should be strengthened, especially in young children, to minimize the risk of complications and long-term symptoms.

### Supplementary Information

Below is the link to the electronic supplementary material.

Supplementary file1 (DOC 5065 KB)

## Data Availability

The data that support the findings of this study are available from the corresponding author, (Feng), upon reasonable request.
