# Supplementary material for: Clinical manifestations of respiratory syncytial virus infection and the risk of wheezing and recurrent wheezing illness: a systematic review and meta-analysis
Source: World J Pediatr. 2023 Aug 2;19(11):1030–40. doi: 10.1007/s12519-023-00743-5 (PMC10533619; doi:10.1007/s12519-023-00743-5)
Supplement: Supplementary file 1 — Supplementary file1 (DOC 5065 KB) [file 12519_2023_743_MOESM1_ESM.doc]

**Supplementary appendix**

**Supplementary methods**

**Supplementary table 1. Search strategy**

| CNKI |
| --- |
| (SU=Respiratory Syncytial Virus OR SU=Respiratory Syncytial Virus Infections OR SU=Human Respiratory Syncytial Virus OR SU=Human orthopulmonary virus OR SU=RSV OR SU=HRSV) AND (SU=Child OR SU=Infant OR SU=Pediatrics OR SU=Toddler OR SU=Baby OR SU=Newborn OR SU=Neonate OR SU=kindergarten OR SU=Preschool OR SU=preschool child OR SU=schoolchild OR SU=school age OR SU=Neonatology department OR SU=Pediatric department OR SU=nurser) AND (SU=HLOS OR SU=length of stay OR SU=LOS OR SU=Hospital stay) AND (SU=Association OR SU=Risk factor OR SU=Prognosis OR SU=complication OR SU=sequela) |
| 48 records |
| CBM |
| #1"Respiratory Syncytial Virus"[Unweighted: extended] OR "Respiratory Syncytial Virus, Human"[Unweighted: extended]  3,954  #2"Respiratory Syncytial Virus"[Common words: Intelligence] OR "Respiratory Syncytial Virus Infections"[Common words: Intelligence] OR "Human Respiratory Syncytial Virus Infections"[Common words: Intelligence] OR "Human orthopulmonary virus"[Common words: Intelligence] OR "RSV"[Common words: Intelligence] OR "HRSV"[Common words: Intelligence]  6,619  #3 #1 OR #2  6,619  #4((("Child"[Unweighted: extended]) OR "Infant, Newborn "[Unweighted: extended]) OR "Toddler"[Unweighted: extended]) OR "Child, Preschool"[Unweighted: extended]  [27,021](javascript:historyLink('((([不加权:扩展]) OR [不加权:扩展]) OR [不加权:扩展]) OR [不加权:扩展]'))  #5"Child"[Common words: Intelligence] OR "Infant"[Common words: Intelligence] OR "Pediatric"[Common words: Intelligence] OR "Toddler"[Common words: Intelligence] OR "Baby"[Common words: Intelligence] OR "child patient"[Common words: Intelligence] OR "Newborn "[Common words: Intelligence] OR "Pediatric department"[Common words: Intelligence] OR "Neonatology department"[Common words: Intelligence]  883,892  #6 #4 OR #5  883,892  #7 (("Hospital stay "[Unweighted: extended]) OR " Hospital length of stay "[Unweighted: extended])  [48,751](javascript:historyLink('(([不加权:扩展]) OR [不加权:扩展])'))  #8 "admission"[Common words: Intelligence] OR "Hospitalization"[Common words: Intelligence] OR "HLOS"[Common words: Intelligence] OR " LOS"[Common words: Intelligence] OR " Hospital stay"[Common words: Intelligence]  917,515  #9 #7 OR #8  917,515  #10 " Prognosis "[Unweighted: extended] OR " Risk factor "[Unweighted: extended]  427,905  #11 " Association"[Common words: Intelligence] OR " Risk factor"[Common words: Intelligence] OR " Prognosis"[Common words: Intelligence] OR " complication"[Common words: Intelligence] OR " sequela"[Common words: Intelligence]  2,426,606  #12 #10 OR #11  2,426,606  #13 #9 OR #12  3,000,582  #14 #3 AND #6 AND #13 AND 2010-2022[data] |
| **1428** records |
| VIP |
| ((((M=Respiratory Syncytial Virus OR M=Respiratory Syncytial Virus Infections) OR M=Respiratory Syncytial Virus，Human) AND (((((((((((((((M=Child OR M=Infant) OR M=Pediatrics) OR M=Neonate) OR M=Baby) OR M=Child patient) OR M=Newborn) OR M=Pediatric department) OR M=Neonatology department) OR M=Infancy) OR M=Toddler) OR M=Preschool) OR M=kindergarten) OR M=nurser) OR M=preschool child) OR M=5 years old)) AND ((((((((M= Incidence OR M= Incidence) OR M= Infection) OR M= Positive serum antibody) OR M=Admission) OR M=Hospitalization) OR M=Hospital stay) OR M=Hospital length of stay) OR M= HLOS) AND (((((M= Association) OR M= Risk factor) OR M= Prognosis) OR M= Complication) OR M= Sequela) |
| 38 records |
| Wanfang |
| theme:("Respiratory Syncytial Virus" or "Respiratory Syncytial Virus Infections " or "Respiratory Syncytial Virus，Human ") and theme:("Child" or "Infant" or "Pediatrics" or "Neonat" or "Baby" or "Child patient" or "Newborn" or "Pediatric department" or "Neonatology department" or "Infancy" or "Toddler" or "Preschool" or "kindergarten" or "preschool child") and theme:(" Incidence" or "Prevalence" or "Infection" or "Positive serum antibody" or "admission" or "hospitalization" or "hospital stay" or "leigh of stay" or "hospital length of stay") and theme:(" Association" or " Risk factor" or " Prognosis" or " Complication " or " Sequela"） |
| 639 records |
| Pubmed |
| 1.(("Respiratory Syncytial Virus Infections"[Mesh]) OR "Respiratory Syncytial Viruses"[Mesh]) OR "Respiratory Syncytial Virus, Human"[Mesh]  5,868  2.((((((respiratory Syncytial Virus Infection*[Title/Abstract]) OR (human respiratory syncytial virus[Title/Abstract])) OR (RSV[Title/Abstract])) OR (respiratory syncytial virus*[Title/Abstract])) OR (HRSV[Title/Abstract])) OR (RS virus[Title/Abstract])) OR (RSV[Title/Abstract])  10,668  3.1 OR 2  11,054  4.(((((("Child, Preschool"[Mesh]) OR "Child"[Mesh]) OR "Infant"[Mesh]) OR "Pediatrics"[Mesh]) OR "Schools, Nursery"[Mesh]) OR "Nurseries, Infant"[Mesh]) OR "Nurseries, Hospital"[Mesh]  941,596  5.(((((((((((((Child*[Title/Abstract]) OR (infant*[Title/Abstract])) OR (pediatric*[Title/Abstract])) OR (Toddler*[Title/Abstract])) OR (Kindergarten*[Title/Abstract])) OR (Preschool*[Title/Abstract])) OR (Baby[Title/Abstract])) OR (babies[Title/Abstract])) OR (Newborn*[Title/Abstract])) OR (Neonat*[Title/Abstract])) OR (pre-school*[Title/Abstract])) OR (nurser*[Title/Abstract])) OR (schoolchild*[Title/Abstract])) OR (school age*[Title/Abstract])  86,427  6.4 OR 5  1,357,568  7.((("Inpatients"[Mesh]) OR "Hospitalization"[Mesh]) OR "Length of Stay"[Mesh])  171,295  8.(((((Inpatient*[Title/Abstract]) OR length of stay [Title/Abstract]) OR LOS[Title/Abstract]) OR Hospital stay [Title/Abstract]) OR HLOS [Title/Abstract])  197,386  9.7 OR 8  296,950  10.3 AND 6 AND 9 Filters: from 2010/1/1 - 2022/6/12  1,360  (("Prognosis"[Mesh]) OR "Risk Factors"[Mesh])  1,476,403  12.(((((Association [Title/Abstract]) OR Risk factor*[Title/Abstract]) OR Prognosis*[Title/Abstract]) OR complication*[Title/Abstract]) OR sequela [Title/Abstract])  2,101,268  13. 11 OR 12  2,892,355  14.13 AND 3 AND 6  1,545  15 "Humans"[MeSH Terms]  8,212,899  16."human"[Title/Abstract] OR "humans"[Title/Abstract]  1,484,954  17.15 OR 16  8,620,349  18.14 AND 17 |
| 1,396 records |
| Embase |
| #1 'respiratory syncytial virus infection'/exp OR 'pneumovirus'/exp OR 'human respiratory syncytial virus'/exp  26,537  #2 'respiratory syncytial virus infection*':ab,ti OR 'human respiratory syncytial virus':ab,ti OR 'rsv':ab,ti OR 'respiratory syncytial virus*':ab,ti OR hrsv:ab,ti OR 'rs virus':ab,ti  26,962  #3 #1 OR #2  34,520  #4 'preschool child'/exp OR 'child'/exp OR 'infant'/exp OR 'pediatrics'/exp OR 'nursery school'/exp OR 'nursery'/exp  3,439,162  #5 child*:ab,ti OR infant*:ab,ti OR pediatric*:ab,ti OR toddler*:ab,ti OR kindergarten*:ab,ti OR preschool*:ab,ti OR baby:ab,ti OR babies:ab,ti OR newborn*:ab,ti OR neonat*:ab,ti OR 'pre school*':ab,ti OR nurser*:ab,ti OR schoolchild*:ab,ti OR 'school age*':ab,ti  3.231,489  #6 #4 OR #5  4,370,064  #7 'incidence'/exp OR 'morbidity'/exp OR 'prevalence'/exp  1,858,341  #8 incidence:ab,ti OR morbidity:ab,ti OR 'attack rate':ab,ti OR 'incidence rate':ab,ti OR 'prevalence rate':ab,ti OR prevalence:ab,ti OR 'sickness rate':ab,ti OR 'incidence rate ratio':ab,ti OR 'infection rate':ab,ti OR 'antibody positive':ab,ti OR 'serum antibody positive rate':ab,ti OR occurrence:ab,ti  3.301322  #9 #7 OR #8  3,749,276  #10 'hospital patient'/exp OR 'hospitalization'/exp OR 'mortality'/exp  1,891,087  #11 'outpatient rate':ab,ti OR 'visiting rate':ab,ti OR 'admission rate':ab,ti OR 'hospitalization rate':ab,ti OR inpatient*:ab,ti OR 'severe rate':ab,ti OR 'fatality rate':ab,ti OR 'case fatality rate':ab,ti OR 'death rate':ab,ti OR mortality:ab,ti OR death:ab,ti  2,602,722  #12 #10 OR #11  3,327,315  #13 #9 OR #12  6,058,730  #14 #3 AND #6 AND #13  7,810  #15 #14 AND (2010:py OR 2011:py OR 2012:py OR 2013:py OR 2014:py OR 2015:py OR 2016:py OR 2017:py OR 2018:py OR 2019:py OR 2020:py OR 2021:py OR 2022:py)  5,615  #16 'human'/exp  25,497,543  #17 human*:ab,ti  3,909,306  #18 #16 OR #17  26,367,343  #19 #15 AND #18  5,418  #20 #19 AND [embase]/lim NOT ([embase]/lim AND [medline]/lim) |
| 2,172 records |
| The Cochrane library |
| 1.MeSH descriptor: [Respiratory Syncytial Virus Infections] explode all trees  243  2.MeSH descriptor: [Respiratory Syncytial Virus, Human] explode all trees  81  3.MeSH descriptor: [Respiratory Syncytial Viruses] explode all trees  193  4.(respiratory Syncytial Virus Infection* OR human respiratory syncytial virus OR RSV OR respiratory syncytial virus* OR HRSV):ti,ab,kw AND (enteral nutrition):ti,ab,kw  3  5.OR/1-4  365  6.MeSH descriptor: [Child, Preschool] explode all trees  31,618  7.MeSH descriptor: [Child] explode all trees  62,176  8.MeSH descriptor: [Infant] explode all trees  35,271  9.MeSH descriptor: [Pediatrics] explode all trees  729  10.MeSH descriptor: [Nurseries, Infant] explode all trees  12  11.MeSH descriptor: [Nurseries, Hospital] explode all trees  35  12.MeSH descriptor: [Nurseries, Infant] explode all trees  12  13.(Child* OR infant* OR pediatric* OR Toddler* OR Kindergarten* OR Preschool* OR Baby OR babies OR Newborn* OR Neonat* OR pre-school* OR nurser* OR schoolchild* OR school age*):ti,ab,kw  239,960  14.OR/ 6-13  239,960  15.#5 AND #14  265  16.(Inpatient* OR length of stay OR LOS OR Hospital stay OR HLOS):ti,ab,kw 72532  17.#15 AND #16  29  18.#17 with Publication Year from 2010 to 2022  18  19.(Association OR Risk factor OR Prognos* OR complication* OR sequela):ti,ab,kw 23735  20.MeSH descriptor: [Prognosis] OR [Risk Factors] explode all trees  182,626  21.#19 OR #20  446,817  22.#18 AND #21 |
| 8 records |

**Description of individual studies included in the analysis**

**Supplementary table 2. Summary of studies that contributed to common clinical manifestations of RSV infection**

| **No** | **The first author** | **Study Period** | **Country** | **Race** | **Study type** | **Data sources** | **Case definition** | **Specimen** | **Diagnostic test** | **Sample size** | **Ages** | **Gender (male/female)** | **QA score** | **Symptoms** |
| --- | --- | --- | --- | --- | --- | --- | --- | --- | --- | --- | --- | --- | --- | --- |
| 1 | T. Zhang [1] | 2005-2009 | China | NA | Observational study | Hospital database | ARI | NS | IF | 2,721 | 0-15Y | 1,717/966 | 8 | o; k; i; j; n; q |
| 2 | C. S. Arriola [2] | 2014-2015 | USA | Hispanic: 360；  White non-Hispanic: 494；  Black non-Hispanic: 375；  Other: 168；  Missing data: 157 | Observational study | National based | RSV confirmed cases | n/a | PCR, rapid antigen  Test, fluorescent antibody, or viral culture | 1,554 | 0-2Y | 895/659 | 10 | o; p; i; d; a; c; g; f; e; j; q |
| 3 | Y. Zhou [3] | 2011 | China | NA | Prospective cohort study | Hospital database | RSV confirmed cases | NPS | IF | 266 | 0-2Y | 190/76 | 8 | k; I; d; g |
| 4 | L. Toivonen [4] | 2008-2010 | Finland | n/a | Prospective cohort study | Birth-cohort | ARI | n/a | PCR | 923 | 0-2Y | 488/435 | 8 | i; j; l |
| 5 | J. Boonyaratanakornkit [5] | 2011-2014 | Nepal | n/a | Prospective cohort study | Birth-cohort | ARI | n/a | PCR | 3,528 | 0-6M | 1,861/1,667 | 8 | i; d; a; h |
| 6 | MI Pei-ming [6] | 2018-2019 | China | n/a | Observational study | Hospital database | Pneumonia | OPS | Double amplification | 842 | 1M-6Y | n/a | 7 | p; k; d; a; b; q |
| 7 | E. A. Okiro [7] | 2002-2004 | Kenya | n/a | Prospective cohort study | National based | ARI | n/a | IF | 2,143 | 0-5Y | 1,069/1094 | 10 | i; d; g |
| 8 | J. P. McCracken [8] | 2007-2012 | Guatemala | NA | Observational study | National based | RSV confirmed cases | NPS and OPS | PCR | 1,356 | 0-5Y | 591/765 | 8 | c; j; h; g |
| 9 | R. Thwaites [9] | 2000-2011 | USA | n/a | Observational study | National based | SARI | n/a | n/a | 13,362 | 0-2Y | 7,554/11,808 | 8 | o; q |
| 10 | C. Svensson [10] | 2004-2011 | Sweden | n/a | Observational study | National based | ARI | NPS | rapid antigen detection | 52,781 | 0-4Y | n/a | 8 | o; p; j; l; q |
| 11 | Y. Kobayashi [11] | 2017-2018 | Japan | n/a | Observational cohort | National based | Medical requirement  RSV | n/a | n/a | 18,220 | 0-2Y | 10,001/8,219 | 7 | o; p; m; q |
| 12 | H. Chi [12] | 2004-2007 | China | n/a | Observational study | Hospital database | ARI | n/a | n/a | 470 | 0-5Y | 276/194 | 7 | o; k; q |
| 13 | Luo Ying-ying [13] | 2012-2014 | China | NA | Observational study | Hospital database | RSV confirmed cases | n/a | n/a | 365 | 0-5Y | n/a | 7 | i; d; g; h; q |
| 14 | Zhang Xiao-bo [14] | 2012-2013 | China | n/a | Observational study | Hospital database | ARI | Airway suction fluid | IF | 1,726 | 0-1Y | 1,150/575 | 7 | m; n; q |
| 15 | K. K. McLaurin [15] | 2003-2013 | USA | NA | Observational study | National based | RSV confirmed cases | n/a | n/a | 38,372 | 0-1Y | n/a | 9 | o; p; q |
| 16 | M. L. Forbes [16] | 2003-2007 | USA | NA | Observational study | National based | RSV confirmed cases | n/a | n/a | 1,983 | 0-1Y | 1,162/820 | 7 | o; q |
| 17 | J. Fergie [17] | 2010-2017 | USA | Wight: 40137;  Black: 10541;  Asian: 1689;  Indian: 473;  The Islamic: 338;  Others: 10271;  Missing: 4121 | Retrospective cohort | National based | RSV confirmed cases | n/a | n/a | 67,570 | 0-6M | 38,177/29,393 | 9 | o; q |
| 18 | J. Nguyen-Van-Tam [18] | 2008-2016 | USA | African-American: 630;  Asian: 491;  White Caucasian: 8781;  Missing: 2939 | Retrospective cohort | National based | RSV confirmed cases | n/a | n/a | 12,841 | 0-2Y | 7,292/5,549 | 8 | i |
| 19 | Fan Li [19] | 2006-2011 | China | NA | Observational study | Hospital database | RSV confirmed cases | Airway suction fluid | IF | 176 | 0-1Y | 113/63 | 7 | i; d; a; b; f |
| 20 | Ren Kang-yi [20] | 2013-2018 | China | NA | Observational study | Hospital database | RSV confirmed cases | Airway suction fluid | PCR | 298 | 0-2Y | 204/94 | 7 | i; d; a; m; h; f; q |
| 21 | Ren Shao-long [21] | 2010-2014 | China | NA | Observational study | Hospital database | RSV confirmed cases | n/a | IF | 4,096 | 29D-5Y | 2,656/1,440 | 8 | o; i; d; c; g; e; q |
| 22 | Yu Yun [22] | 2015-2018 | China | NA | Observational study | Hospital database | RSV confirmed cases with pneumonia | Airway suction fluid | IF | 152 | 1-4W | 101/51 | 7 | D; a; m; g |
| 23 | S. A. Buchan [23] | 2009-2014 | Canada | NA | Observational study | Hospital database | RSV confirmed cases | n/a | PCR or viral culture or IF | 6,364 | 0-5Y | 3,612/2,752 | 8 | o; p; q |
| 24 | N. Halasa [24] | 2010-2013 | Jordan | n/a | Observational study | Hospital database | ARI | NS or OPS | PCR | 3,168 | 0-2Y | 1,912/1,256 | 8 | o; p; k; i; d; a; c; j; g; f; e; n; q |
| 25 | R. S. Linssen [25] | 2003-2016 | Netherlands | NA | Observational study | National based | RSV confirmed cases | n/a | n/a | 2,161 | 0-2Y | n/a | 9 | o; q |
| 26 | E. J. Anderson [26] | 2014-2016 | USA | NA | Observational study | National based | RSV confirmed cases | n/a | n/a | 1,378 | 0-1Y | n/a | 9 | o; q |
| 27 | Q. Zhong [27] | 2009-2015 | China | NA | Retrospective cohort | Hospital database | RSV confirmed cases | NPS | IF | 341 | 0-28D | 188/153 | 9 | I; d; a; g; f |
| 28 | P. K. Munywoki [28] | 2002-2010 | Kenya | NA | Retrospective cohort | Hospital database | RSV confirmed cases | NPS | IF | 560 | 0-5Y | 296/264 | 8 | a; j; q |
| 29 | A. Mejias [29] | 2010-2016 | USA | NA | Observational study | National based | RSV confirmed cases | n/a | n/a | 124,439 | 0-5Y | 66,034/58,405 | 10 | p; k; i; q |
| 30 | J. R. Romero [30] | 2001-2004 | USA | NA | Retrospective cohort study | Hospital database | RSV confirmed preterm or low birth weight infants | n/a | n/a | 378 | 0-3Y | 215/163 | 9 | i |
| 31 | H. Brenes-Chacon [31] | 2014-2018 | USA | Whit: 339;  Black: 113;  Others: 82 | Observational study | Hospital database | RSV confirmed cases | NPS | PCR | 534 | 0-2Y | 285/249 | 8 | I; d; a; c; f; e |
| 32 | S. Vizcarra-Ugalde [32] | 2003-2014 | Mexico | n/a | Observational study | Hospital database | RSV confirmed cases | NPS | IF or PCR | 1,153 | 0-5Y | n/a | 7 | o; q |
| 33 | C. Demont [33] | 2010-2018 | France | n/a | Observational study | National based | ARI | n/a | n/a | 23,835,288 | 0-5Y | n/a | 8 | q |
| 34 | R. Kramer [34] | 2012-2016 | France | n/a | Observational study | Hospital database | ARI | n/a | n/a | 21,930 | 0-1Y | n/a | 7 | q |
| 35 | A. M. Helfrich [35] | 2005-2011 | Argentina | n/a | Observational study | National based | ARI | n/a | n/a | 599,535 | 0-1Y | n/a | 8 | q |

*RSV* respiratory syncytial virus, *USA* the United States of America, *ARI* acute respiratory infection, *ILI* influenza-like illness, *SARI* severe acute respiratory tract infection, *LRTI* lower respiratory tract infection, *NPS* nasopharyngeal swab, *NS* nose swab, *OPS* oropharyngeal swab, *NLF* nasal lavage fluid, *PCR* polymerase chain reaction, *IF* immunofluorescence, *QA* quality assessment, *n/a* not analyzed, *a* Cough, *b* nasal congestion, *c* rhinorrhea, *d* fever, *e* vomiting, *f* diarrhea, *g* shortness of breath, *h* dyspnea, *i* wheezing, *j* pneumonia, *k* oxygen, *l* otitis media, *m* respiratory failure, *n* sepsis, *o* ICU admission, *p* mechanical ventilation, *q* hospital length of stay.

**Supplementary table 3.** **Summary of studies that contributed to overall impact of RSV infection on the occurrence of wheezing when compared with non-infected children**

| **No** | **The first author** | **Study Period** | **Country** | **Race** | **Study type** | **Data sources** | **Case and matched control** | **Specimen** | **Diagnostic test** | **Sample size** | **Ages** | **Gender (male/female)** | **QA score** |
| --- | --- | --- | --- | --- | --- | --- | --- | --- | --- | --- | --- | --- | --- |
| 1 | Feng Ying [36] | 2009-2010 | China | NA | Observational study | Hospital database | Wheezing and non-wheezing patients among hospitalized children with community-acquired pneumonia | Airway suction fluid | IF | 1,106 | 0-3 | 742/364 | 8 |
| 2 | Liu Feng [37] | 2012-2016 | China | n/a | Observational study | Hospital database | Wheezing and non-wheezing patients among acute upper respiratory tract infection or bronchitis children | n/a | n/a | 410 | 0-3 | 298/112 | 7 |
| 3 | Su Hong-jie [38] | 2016-2017 | China | n/a | Observational study | Hospital database | Wheezing and non-wheezing patients among respiratory tract infection children | blood | IF | 315 | 0-3 | 162/153 | 8 |
| 4 | Yang Sai [39] | 2010-2012 | China | n/a | Observational study | Hospital database | Infants with wheezing bronchiolitis or wheezing bronchopneumonia diagnosed as bronchiolitis and infants hospitalized with no wheezing diagnosed as acute upper respiratory tract infection, bronchitis and bronchopneumonia | n/a | IF | 600 | 0-3 | 226/74 | 9 |
| 5 | Zhong Qiu-lan [40] | 2012-2014 | China | n/a | Observational study | Hospital database | wheezing children with bronchial pneumonia, bronchitis and bronchiolitis were matched with children acquired the same disease at the same time but without wheezing | n/a | IF | 204 | 0-3 | 123/81 | 7 |
| 6 | Lian Peng-qiang [41] | 2016-2018 | China | n/a | Observational study | Hospital database | Children with asthmatic bronchitis were matched to children with lower respiratory tract infection without wheezing symptoms | Airway suction fluid | PCR | 200 | 0-3 | 104/96 | 8 |
| 7 | Wang Kun [42] | 2016-2021 | China | NA | Observational study | Hospital database | Children with asthmatic bronchitis were matched with healthy children at the same time | blood | Serology | 200 | 0-3 | 131/69 | 8 |

*RSV* respiratory syncytial virus, *ARI* acute respiratory infection, *ILI* influenza-like illness, *SARI* severe acute respiratory tract infection, *LRTI* lower respiratory tract infection, *NPS* nasopharyngeal swab, *NS* nose swab, *OPS* oropharyngeal swab, *NLF* nasal lavage fluid, *PCR* polymerase chain reaction, *IF* immunofluorescence, *QA* quality assessment, *n/a* not analyzed.

**Supplementary table 4.** **Summary of studies that contributed to overall impact of RSV infection in early childhood on subsequent development of recurrent wheeze illness when compared with non-infected children**

| **No** | **The first author** | **Study Period** | **Country** | **Race** | **Study type** | **Data sources** | **Case and matched control** | **Specimen** | **Diagnostic test** | **Sample size** | **Ages (year)** | **Infected time (year)** | **Gender (male/female)** | **QA score** |
| --- | --- | --- | --- | --- | --- | --- | --- | --- | --- | --- | --- | --- | --- | --- |
| 1 | M. O. Blanken [43] | NA | Netherlands | n/a | Prospective cohort study | National based | Children with recurrent wheezing were matched with healthy children | n/a | n/a | 3,952 | 0-1 | 0-1 | 2192/1756 | 6 |
| 2 | Zhai Jia [44] | 2015-2016 | China | n/a | Observational study | Hospital database | Patients with recurrent wheezing at the first diagnosis matched with children without recurrent wheezing | Airway suction fluid | n/a | 145 | 0-4 | 0-3 | 50/30 | 7 |
| 3 | G. J. Escobar [45] | 1996-2002 | USA | Caucasian: 30289；  African-American : 6541；  Asian: 13865；  Spanish: 15358；  Others: 5048 | Retrospective cohort study | National based | Children with recurrent wheezing matched with healthy children | n/a | n/a | 71,102 | 0-3 | 0-1 | 36404/34698 | 9 |
| 4 | J. R. Romero [30] | 2001-2004 | USA | n/a | Retrospective cohort study | National based | RSV infected children and matched uninfected children | n/a | PCR | 984 | 0-3 | 0-1 | 619/365 | 8 |
| 5 | A. Mejias [29] | 2010-2016 | USA | n/a | Retrospective cohort study | National based | RSV infected children and matched uninfected children | n/a | n/a | 124,439 pairs | 0-5 | 0-1 | 66034/58405 pairs | 9 |
| 6 | E. A. F. Simões [46] | 2016-2020 | India | n/a | Retrospective cohort study | National based | RSV infected patients and matched uninfected LRTI patients | NPS | PCR | 23,763 | 0-2Y | 0-2 | premature: 8943/2203；  term: 19127/81025 | 8 |
| 7 | M. B. Jalink [47] | 1998-2009 | Canada | n/a | Observational study | National based | RSV infected patients and matched uninfected ARI patients | n/a | n/a | 3,916 | 0-5Y | **?** | 2098/1818 | 8 |

*RSV* respiratory syncytial virus, *USA* the United States of America, *ARI* acute respiratory infection, *ILI* influenza-like illness, *SARI* severe acute respiratory tract infection, *LRTI* lower respiratory tract infection, *NPS* nasopharyngeal swab, *NS* nose swab, *OPS* oropharyngeal swab, *NLF* nasal lavage fluid, *PCR* polymerase chain reaction, *IF* immunofluorescence, *QA* quality assessment; n/a not analyzed.

**Supplementary table 5. Quality scoring criteria for cross-sectional study studies**

| **No** | **The first author** | **Study Period** | **①** | **②** | **③** | **④** | **⑤** | **⑥** | **⑦** | **⑧** | **⑨** | **⑩** | **⑪** | **QA score** | **Outcomes** |
| --- | --- | --- | --- | --- | --- | --- | --- | --- | --- | --- | --- | --- | --- | --- | --- |
| 1 | T. Zhang | 2005-2009 | 1 | 1 | 1 | 0 | 1 | 1 | 0 | 1 | 0 | 1 | 1 | 8 | **a** |
| 2 | C. S. Arriola | 2014-2015 | 1 | 1 | 1 | 1 | 1 | 1 | 1 | 1 | 0 | 1 | 1 | 10 | **a** |
| 3 | R. Thwaites | 2000-2011 | 1 | 1 | 1 | 1 | 0 | 1 | 0 | 1 | 0 | 1 | 1 | 8 | **a** |
| 4 | C. Svensson | 2004-2011 | 1 | 1 | 1 | 0 | 0 | 1 | 1 | 1 | 0 | 1 | 1 | 8 | **a** |
| 5 | Y. Kobayashi | 2017-2018 | 1 | 0 | 1 | 0 | 1 | 1 | 0 | 1 | 0 | 1 | 1 | 7 | **a** |
| 6 | H. Chi | 2004-2007 | 1 | 1 | 1 | 1 | 0 | 0 | 0 | 1 | 0 | 1 | 1 | 7 | **a** |
| 7 | K. K. McLaurin | 2003-2013 | 1 | 1 | 1 | 1 | 0 | 1 | 1 | 1 | 0 | 1 | 1 | 9 | **a** |
| 8 | M. L. Forbes | 2003-2007 | 1 | 1 | 1 | 0 | 1 |  | 0 | 1 | 0 | 1 | 1 | 7 | **a** |
| 9 | J. Fergie | 2010-2017 | 1 | 1 | 1 | 0 | 1 | 1 | 1 | 1 | 0 | 1 | 1 | 9 | **a** |
| 10 | Ren Sao-long | 2010-2014 | 1 | 1 | 1 | 0 | 1 | 1 | 0 | 1 | 0 | 1 | 1 | 8 | **a** |
| 11 | S. A. Buchan | 2009-2014 | 1 | 1 | 1 | 0 | 1 | 1 | 0 | 1 | 0 | 1 | 1 | 8 | **a** |
| 12 | N. Halasa | 2010-2013 | 1 | 1 | 1 | 0 | 1 | 1 | 0 | 1 | 0 | 1 | 1 | 8 | **a** |
| 13 | Z. F. Lin | 2003-2016 | 1 | 1 | 1 | 1 | 0 | 1 | 1 | 1 | 0 | 1 | 1 | 9 | **a** |
| 14 | E. J. Anderson | 2014-2016 | 1 | 1 | 1 | 1 | 0 | 1 | 1 | 1 | 0 | 1 | 1 | 9 | **a** |
| 15 | S. Vizcarra-Ugalde | 2003-2014 | 1 | 1 | 1 | 0 | 0 | 1 | 0 | 1 | 0 | 1 | 1 | 7 | **a** |
| 16 | Y. Zhou | 2011 | 1 | 1 | 1 | 0 | 1 | 1 | 0 | 1 | 0 | 1 | 1 | 8 | **a** |
| 17 | L. Toivonen | 2008-2010 | 1 | 1 | 1 | 1 | 1 | 1 | 0 | 1 | 0 | 0 | 1 | 8 | **a** |
| 18 | J. Boonyaratanakornkit | 2011-2014 | 1 | 1 | 1 | 1 | 1 | 1 | 0 | 1 | 0 | 0 | 1 | 8 | **a** |
| 19 | MI Pei-ming | 2018-2019 | 1 | 1 | 1 | 0 | 0 | 1 | 0 | 1 | 0 | 1 | 1 | 7 | **a** |
| 20 | E. A. Okiro | 2002-2004 | 1 | 1 | 1 | 1 | 1 | 1 | 1 | 1 | 0 | 1 | 1 | 10 | **a** |
| 21 | N. I. Mazur | 2007-2012 | 1 | 1 | 1 | 0 | 1 | 1 | 0 | 1 | 0 | 1 | 1 | 8 | **a** |
| 22 | Luo Ying-ying | 2012-2014 | 1 | 1 | 1 | 0 | 0 | 1 | 0 | 1 | 0 | 1 | 1 | 7 | **a** |
| 23 | Zhang-Xiao-bo | 2012-2013 | 1 | 1 | 1 | 0 | 0 | 1 | 0 | 1 | 0 | 1 | 1 | 7 | **a** |
| 24 | V. Wyffels | 2008-2016 | 1 | 1 | 1 | 0 | 1 | 1 | 0 | 1 | 0 | 1 | 1 | 8 | **a** |
| 25 | Fan li | 2006-2011 | 1 | 1 | 1 | 0 | 1 | 0 | 0 | 1 | 0 | 1 | 1 | 7 | **a** |
| 26 | Ren Kang-yi | 2013-2018 | 1 | 1 | 1 | 0 | 1 | 0 | 0 | 1 | 0 | 1 | 1 | 7 | **a** |
| 27 | Yu Yun | 2015-2018 | 1 | 1 | 1 | 0 | 0 | 1 | 0 | 1 | 0 | 1 | 1 | 7 | **a** |
| 28 | Q. Zhong | 2009-2015 | 1 | 1 | 1 | 0 | 1 | 1 | 1 | 1 | 0 | 1 | 1 | 9 | **a** |
| 29 | P. K. Munywoki | 2002-2010 | 1 | 1 | 1 | 0 | 1 | 1 | 0 | 1 | 0 | 1 | 1 | 8 | **a** |
| 30 | H. Brenes-Chacon | 2014-2018 | 1 | 1 | 1 | 0 | 1 | 1 | 0 | 1 | 0 | 1 | 1 | 8 | **a** |
| 31 | C. Demont | 2010-2018 | 1 | 1 | 1 | 1 | 0 | 1 | 0 | 1 | 0 | 1 | 1 | 8 | **a** |
| 32 | R. Kramer | 2012-2016 | 1 | 1 | 1 | 0 | 0 | 1 | 0 | 1 | 0 | 1 | 1 | 7 | **a** |
| 33 | A. M. Helfrich | 2005-2011 | 1 | 1 | 1 | 0 | 0 | 1 | 1 | 1 | 0 | 1 | 1 | 8 | **a** |
| 34 | Feng Ying | 2009-2010 | 1 | 1 | 1 | 0 | 1 | 1 | 0 | 1 | 0 | 1 | 1 | 8 | b |
| 35 | Liu Feng | 2012-2016 | 1 | 1 | 1 | 0 | 0 | 1 | 0 | 1 | 0 | 1 | 1 | 7 | b |
| 36 | Su Hong-jie | 2016-2017 | 1 | 1 | 1 | 0 | 1 | 1 | 0 | 1 | 0 | 1 | 1 | 8 | b |
| 37 | Yang sai | 2010-2012 | 1 | 1 | 1 | 1 | 1 | 1 | 0 | 1 | 0 | 1 | 1 | 9 | b |
| 38 | Zhong Qiu-lan | 2012-2014 | 1 | 1 | 1 | 0 | 0 | 1 | 0 | 1 | 0 | 1 | 1 | 7 | b |
| 39 | Lian Peng-qiang | 2016-2018 | 1 | 1 | 1 | 0 | 1 | 1 | 0 | 1 | 0 | 1 | 1 | 8 | b |
| 40 | Wang Kun | 2016-2021 | 1 | 1 | 1 | 0 | 1 | 1 | 0 | 1 | 0 | 1 | 1 | 8 | b |

*a* Clinical characteristic, *b* Wheezing.

To score of included articles by using items below (the answer is yes, get 1 score, otherwise get 0):

1. Define the source of information (survey, record review);
2. List inclusion and exclusion criteria for exposed and unexposed subjects (cases and control) or refer to previous publications;
3. Indicate time period used for identifying patients;
4. Indicate whether subjects were consecutive if not population-based;
5. Indicate if evaluators of subjective components of study were masked to other aspects of the status of the participants;
6. Describe any assessments undertaken for quality assurance purposes (e. g. test/retest of primary outcome measurements);
7. Explain any patient exclusions from analysis;
8. Describe how confounding was assessed and/or controlled;
9. If applicable, explain how missing data were handled in the analysis;
10. Summarize patient response rates and completeness of data collection;
11. Clarify what follow-up, if any, was expected and the percentage of patients for which incomplete data or follow-up was obtained;

**Supplementary table 6. Quality scoring criteria for cohort study studies**

| **No** | **The first author** | **Study Period** | **①** | **②** | **③** | **④** | **⑤** | **⑥** | **⑦** | **⑧** | **QA score** | **Outcomes** |
| --- | --- | --- | --- | --- | --- | --- | --- | --- | --- | --- | --- | --- |
| 1 | M. O. Blanken | NA | 1 | 1 | 0 | 1 | 2 | 0 | 1 | 1 | 6 | **b** |
| 2 | G. J. Escobar | 1996-2002 | 1 | 1 | 1 | 1 | 2 | 1 | 1 | 1 | 9 | **b** |
| 3 | J. R. Romero | 2001-2004 | 0 | 1 | 1 | 1 | 2 | 1 | 1 | 1 | 8 | **a, b** |
| 4 | A. Mejias | 2010-2016 | 1 | 1 | 1 | 1 | 2 | 1 | 1 | 1 | 9 | **a, b** |
| 5 | E. A. F. Simões | 2004-2015 | 1 | 1 | 1 | 1 | 2 | 0 | 1 | 1 | 8 | **b** |
| 6 | M. B. Jalink | 1998-2009 | 0 | 1 | 1 | 1 | 2 | 1 | 1 | 1 | 8 | **b** |
| 7 | Zhai Jia | 2015-2016 | 0 | 1 | 1 | 1 | 2 | 0 | 1 | 1 | 7 | **b** |

*a* Clinical characteristic, *b* Wheezing.

1. Representativeness of the exposed group (1 score: truly represents the characteristics of the exposed group in the population; 0 score: select a certain group of people/not described);
2. Selection method of the non-exposed group (1 score: from the same population as the exposed group);
3. Determination of exposure factors (1 score: fixed archival records or structured interviews; 0 score: self-reported by subjects);
4. The outcome index of the study's initiation fashion (1 score);
5. The comparability of exposed and unexposed groups was considered in the design and statistical analysis (2 marks: confounding factors were controlled in the study);
6. Whether the evaluation of the results is sufficient (1 score: blind independent evaluation/recorded by archives; 0 score: self-report);
7. Whether the follow-up is long enough after the occurrence of results (1 score: the appropriate follow-up time is stipulated before the evaluation)
8. Whether the exposed group and the non-exposed group were followed up adequately (1 score: no loss to follow up or little loss to follow up but no bias was introduced);

**Supplementary table 7. Hospital length of stay for RSV infected children**

| **First author** | **Study period** | **Subgroups** | **Simple size** | **HLOS (days)** |
| --- | --- | --- | --- | --- |
| **Medium (16 studies included)** | | | | **3.4** |
| **Mean (8 studies included)** | | | | **4.5*** |
| **0-6 month** | | | | |
| H. Chi | 2004-2007 | 0-1M with underlying disease | 943 | 9.9* |
|  |  | 0-1M without underlying disease | 10138 | 6.1* |
| J. Fergie | 2010-2017 | 0-6M | 67570 | 3.0 |
| E. J. Anderson | 2014-2016 | 0-6M | 1074 | 5.5 |
| **Medium (2 studies included)** | | | | **3.0** |
| **Mean (1 studies included)** | | | | **6.4*** |
| **0-1 years old** | | | | |
| M. L. Forbes | 2003-2007 | 0-1Y | 1983 | 3.4* |
| K. K. McLaurin | 2003-2013 | Medicaid 0-90D | 24487 | 4.3* |
|  |  | Medicaid 0-1Y | 12699 | 4.7* |
|  |  | Commercial 0-90D | 13885 | 4.1* |
|  |  | Commercial 0-1Y | 7478 | 6.0* |
| C. Svensson | 2004-2011 | 0-1Y | 1764 | 3.0 |
| A. M. Helfrich | 2005-2011 | 0-1Y | 6954 | 2.0 |
| C. Demont | 2010-2018 | 0-1Y | 35228 | 3.0 |
| Zhang Xiao-bo | 2012-2013 | 0-1Y | 295 | 12.0 |
| R. Kramer | 2012-2016 | 0-1Y | 350 | 4.0 |
| E. J. Anderson | 2014-2016 | 0-1Y | 1378 | 4.0 |
| **Medium (6 studies included)** | | | | **2.9** |
| **Mean (2 studies included)** | | | | **4.5*** |
| **0-2 years old** | | | | |
| R. Thwaites | 2000-2011 | 0-2Y | 13362 | 2.0 |
| Z. F. Lin | 2003-2016 | 0-2Y | 2161 | 13.5 |
| T. Zhang | 2005-2009 | 0-2Y | 849 | 8* |
| N. Halasa | 2010-2013 | 0-2Y | 1397 | 4.0 |
| Ren Kang-yi | 2013-2018 | 0-2Y | 826 | 6.0 |
| C. S. Arriola | 2014-2015 | 0-2Y | 1554 | 3.0 |
| Y. Kobayashi | 2017-2018 | 0-2Y in 2017 | 9711 | 5.5 |
|  |  | 0-2Y in 2018 | 8509 | 5.8 |
| **Medium (6 studies included)** | | | | **4.6** |
| **Mean (1 studies included)** | | | | **8*** |
| **0-5 years old** | | | | |
| P. K. Munywoki | 2002-2010 | 0-5Y | 560 | 4.0 |
| S. Vizcarra-Ugalde | 2003-2014 | 0-5Y | 1153 | 5.0* |
| S. A. Buchan | 2009-2014 | 0-5Y | 6364 | 3.0 |
| Ren Shao-long | 2010-2014 | 29D-5Y | 4096 | 8 |
| C. Demont | 2010-2018 | 1-5Y | 15652 | 2.0 |
| A. Mejias | 2010-2016 | 0-5Y | 124439 | 4.2* |
| Luo Ying-ying | 2012-2014 | 0-5Y | 365 | 9.7* |
| MI Pei-ming | 2018-2019 | 1M-6Y | 842 | 7.2* |
| **Medium (4 studies included)** | | | | **3.2** |
| **Mean (4 studies included)** | | | | **4.2*** |

*Mean of HOLS; *HOLS* Hospital length of stay, *Y* years, *M* month, *D* days.

| 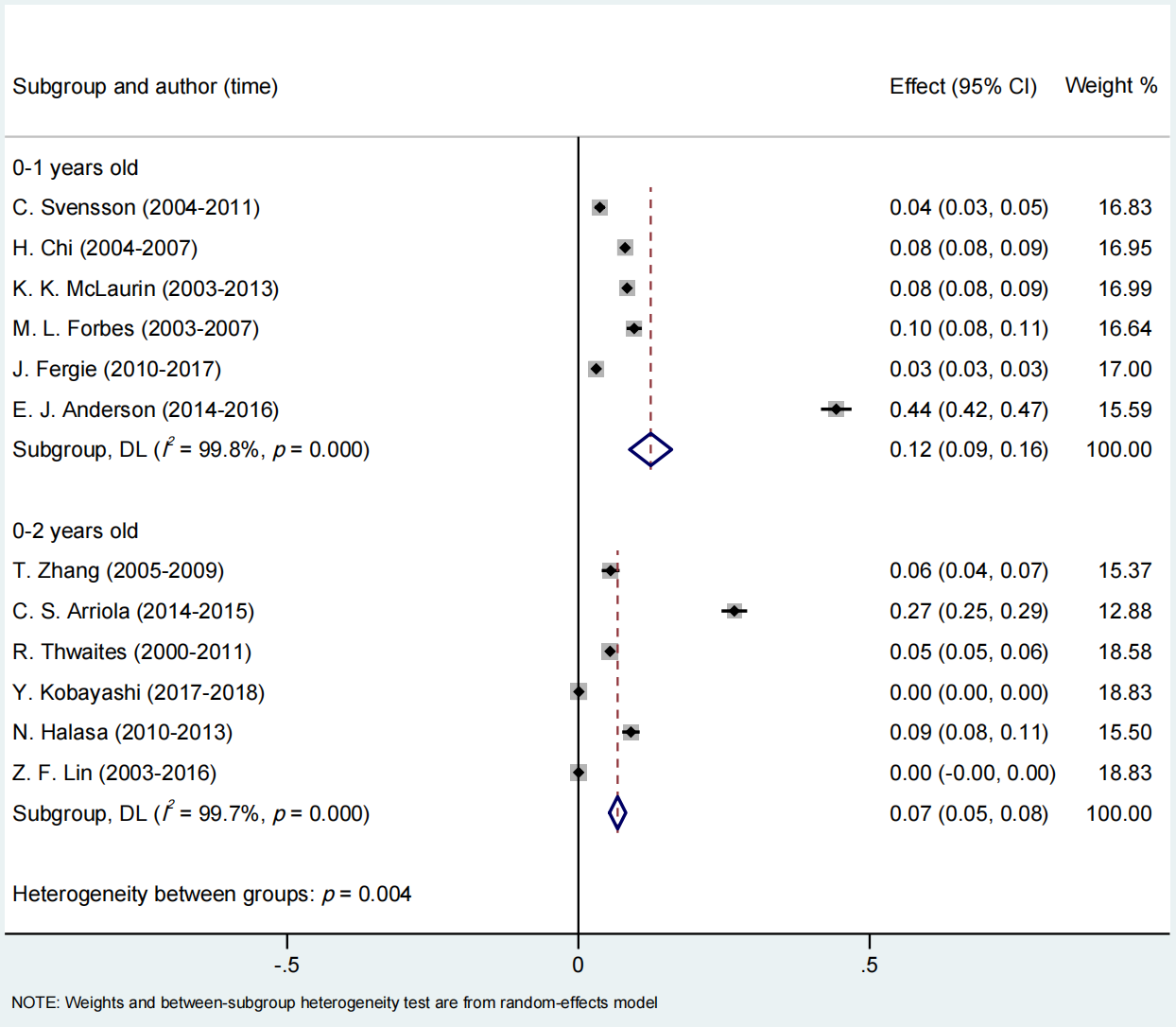 | 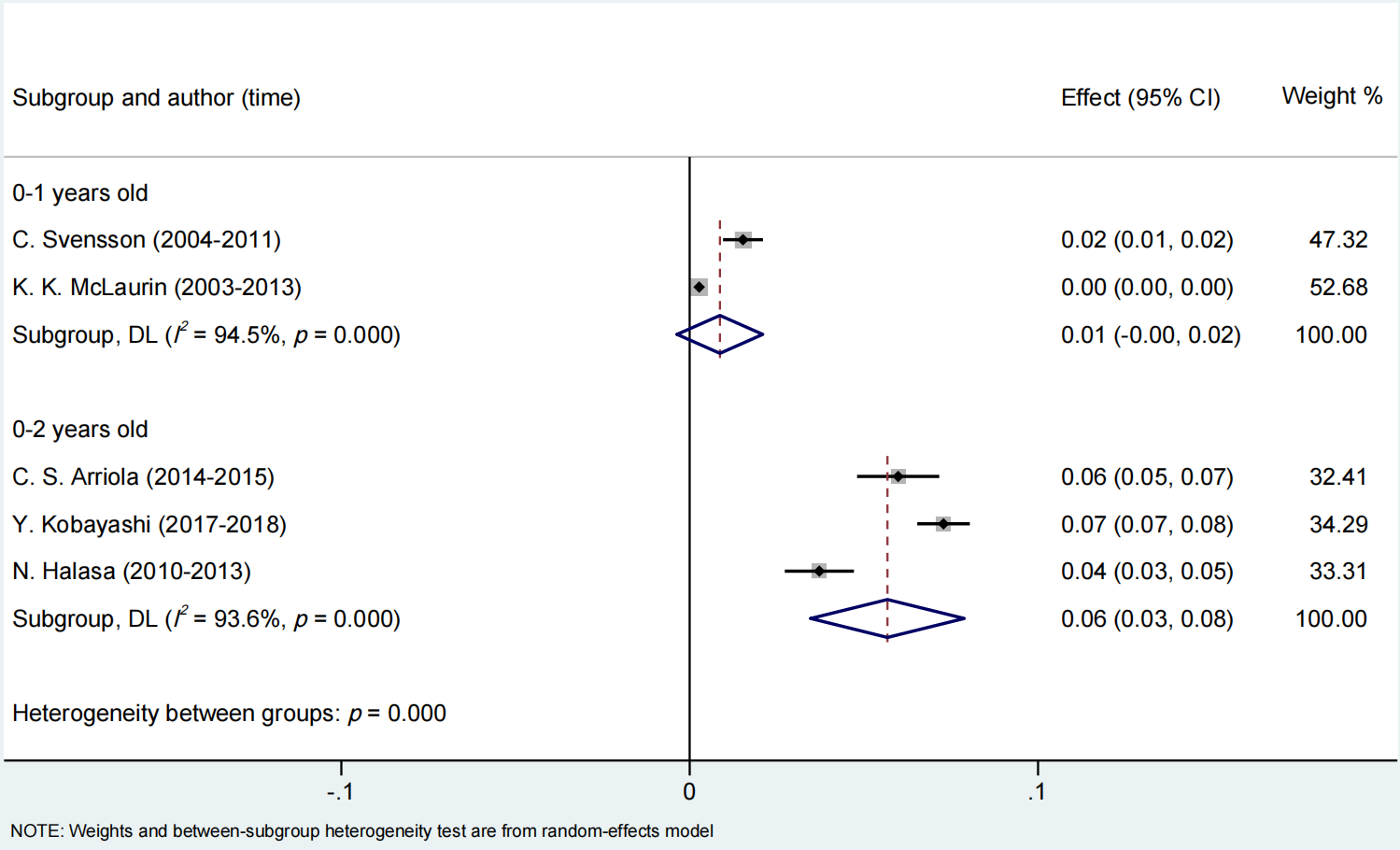 |
| --- | --- |
| **ICU admission** | **Mechanical ventilation** |
| 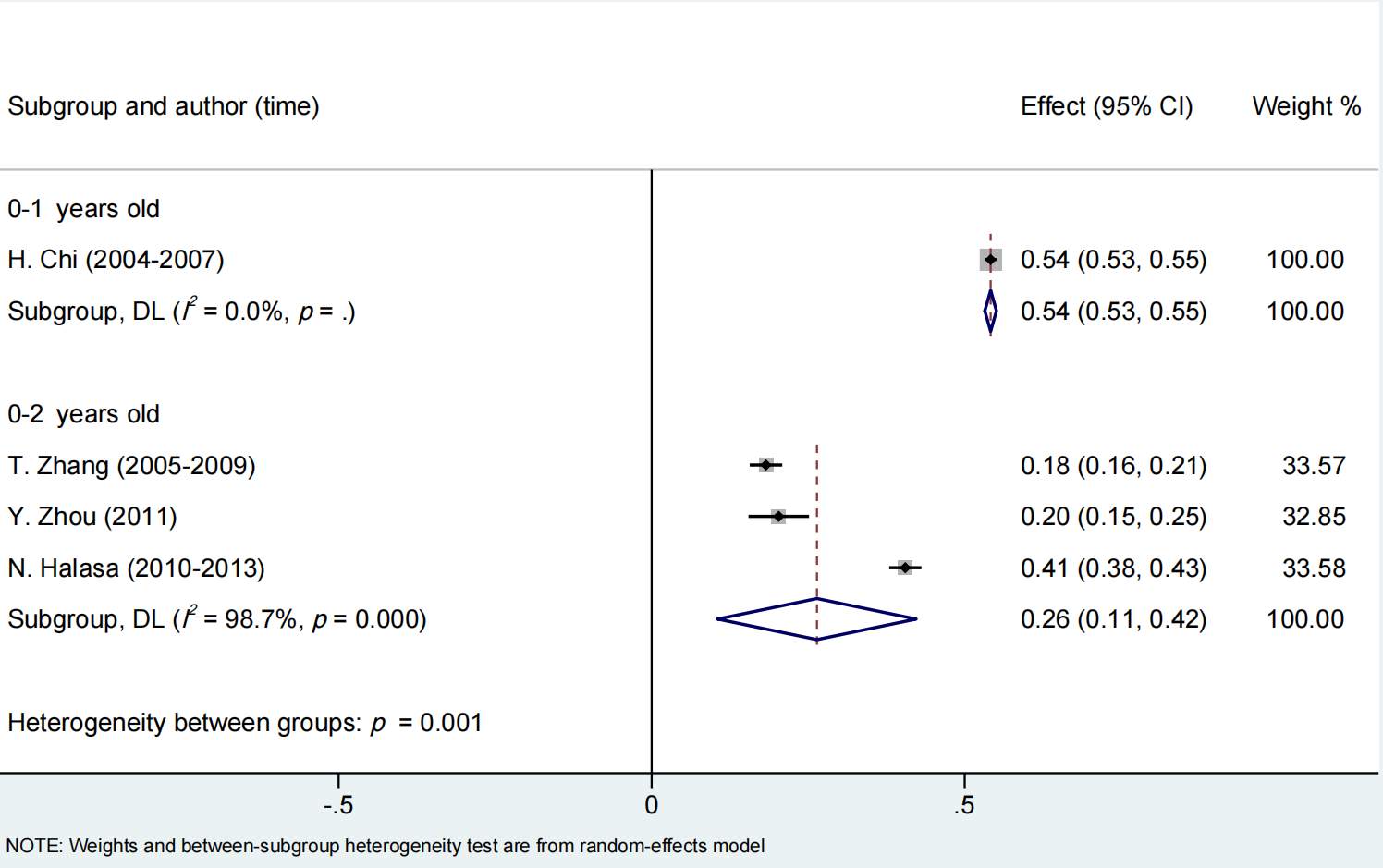 | 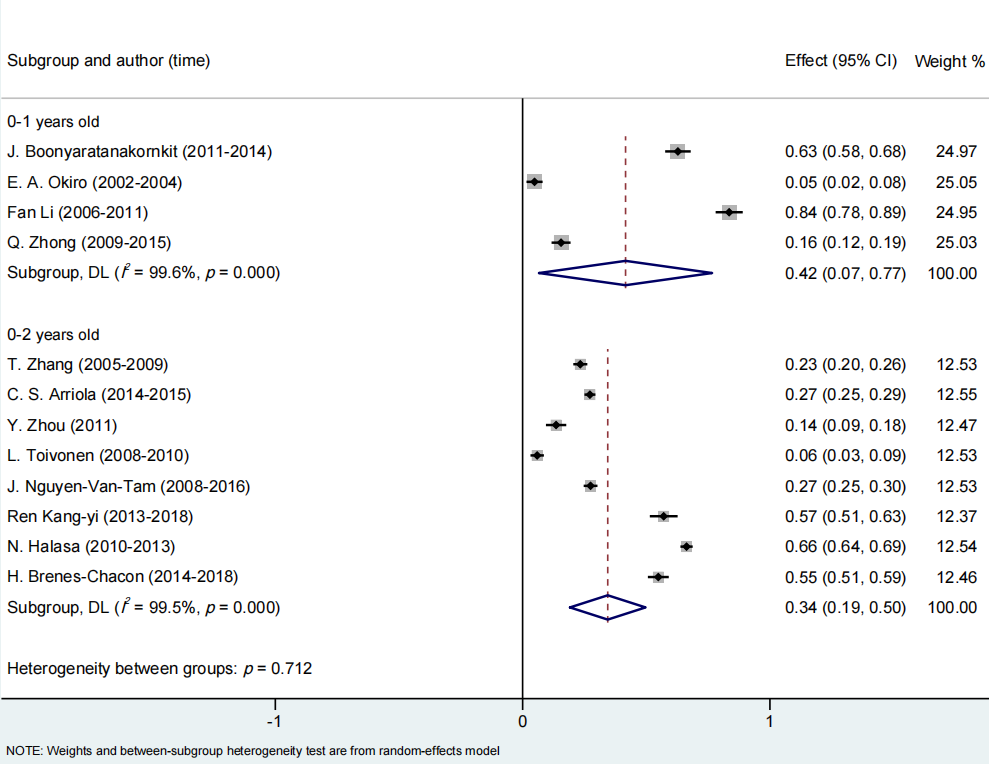 |
| **Oxygen supplementation** | **Wheezing** |
| 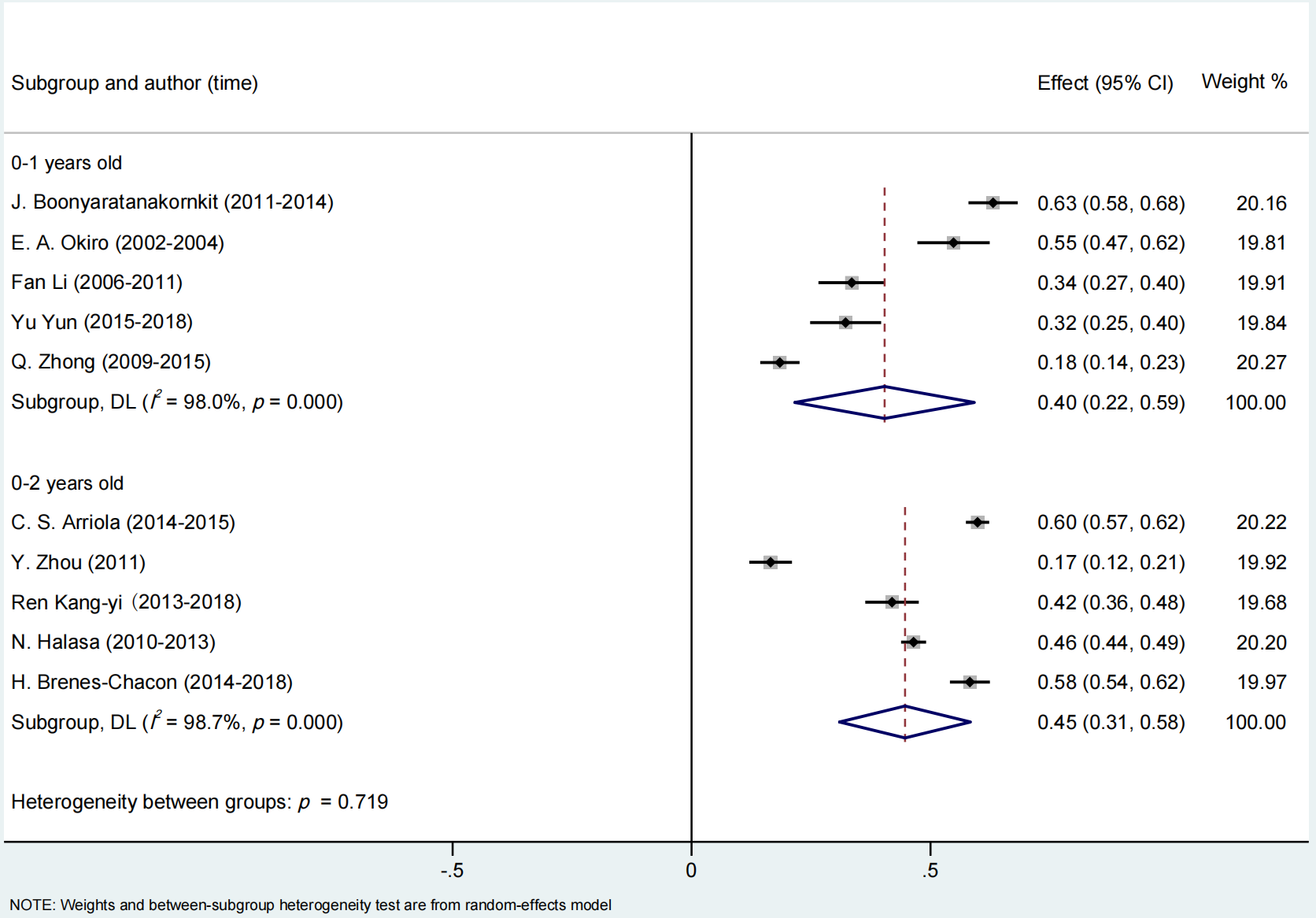 | 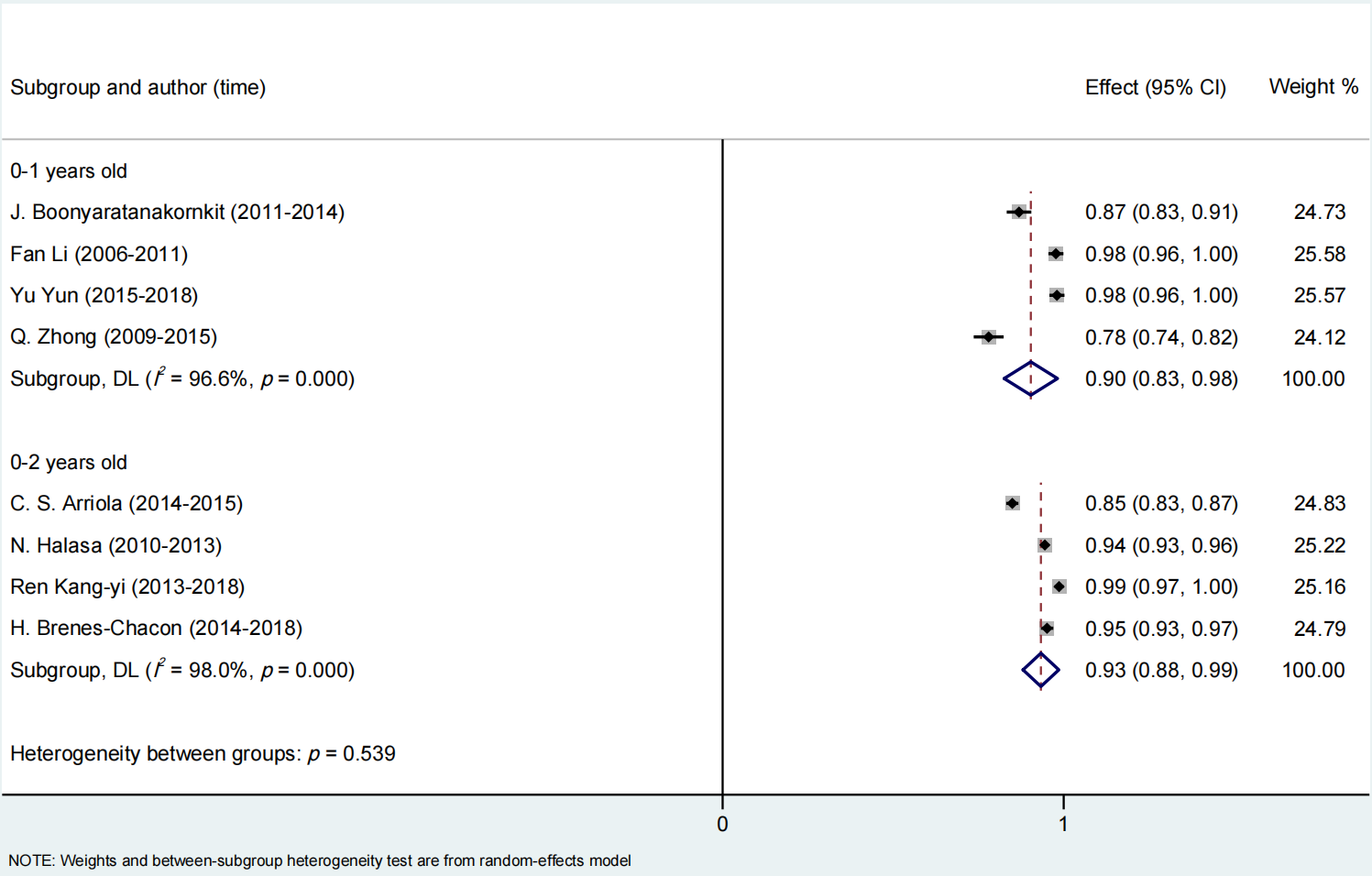 |
| **Fever** | **Cough** |
| 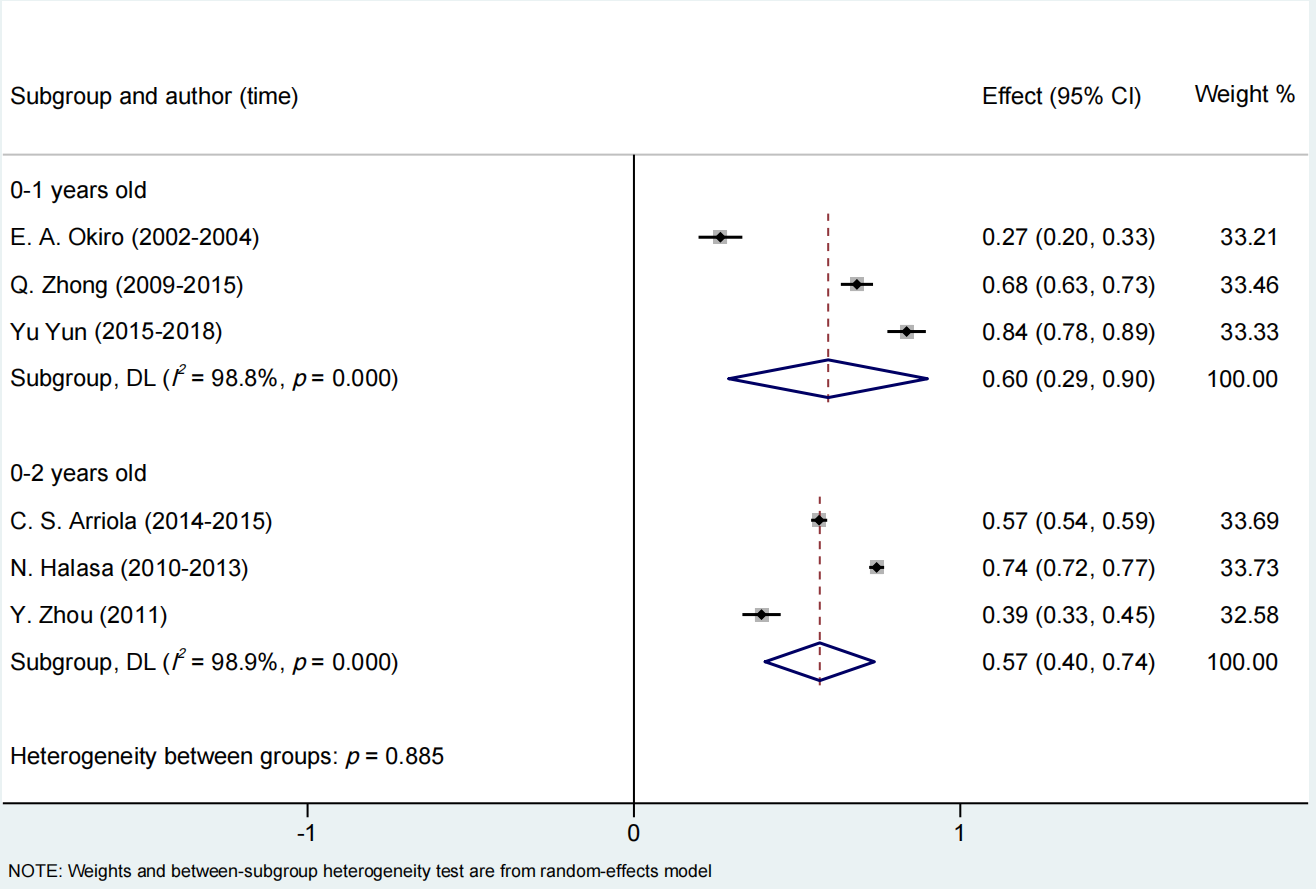 | 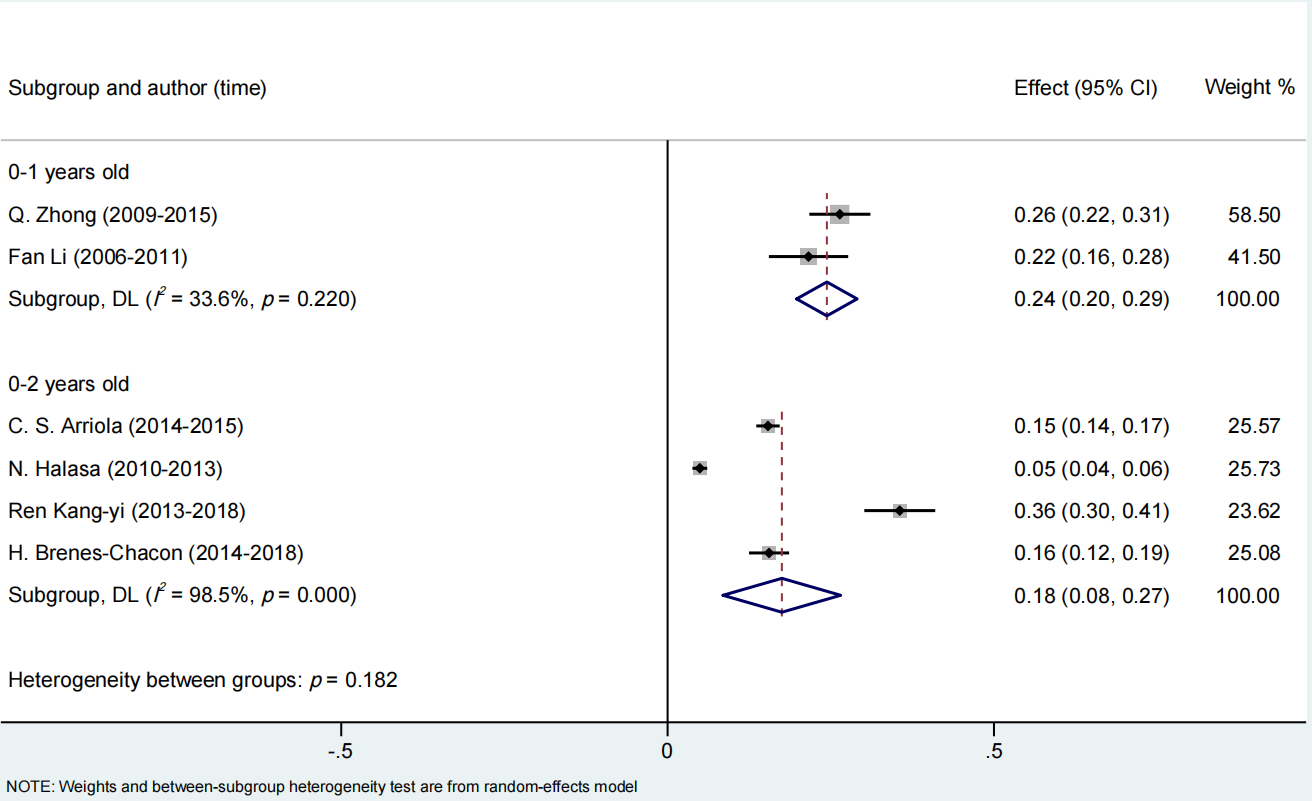 |
| **Shortness of breath** | **Diarrhea** |

**Supplementary figure 1. Common clinical manifestations of RSV infected children in different age groups**

| 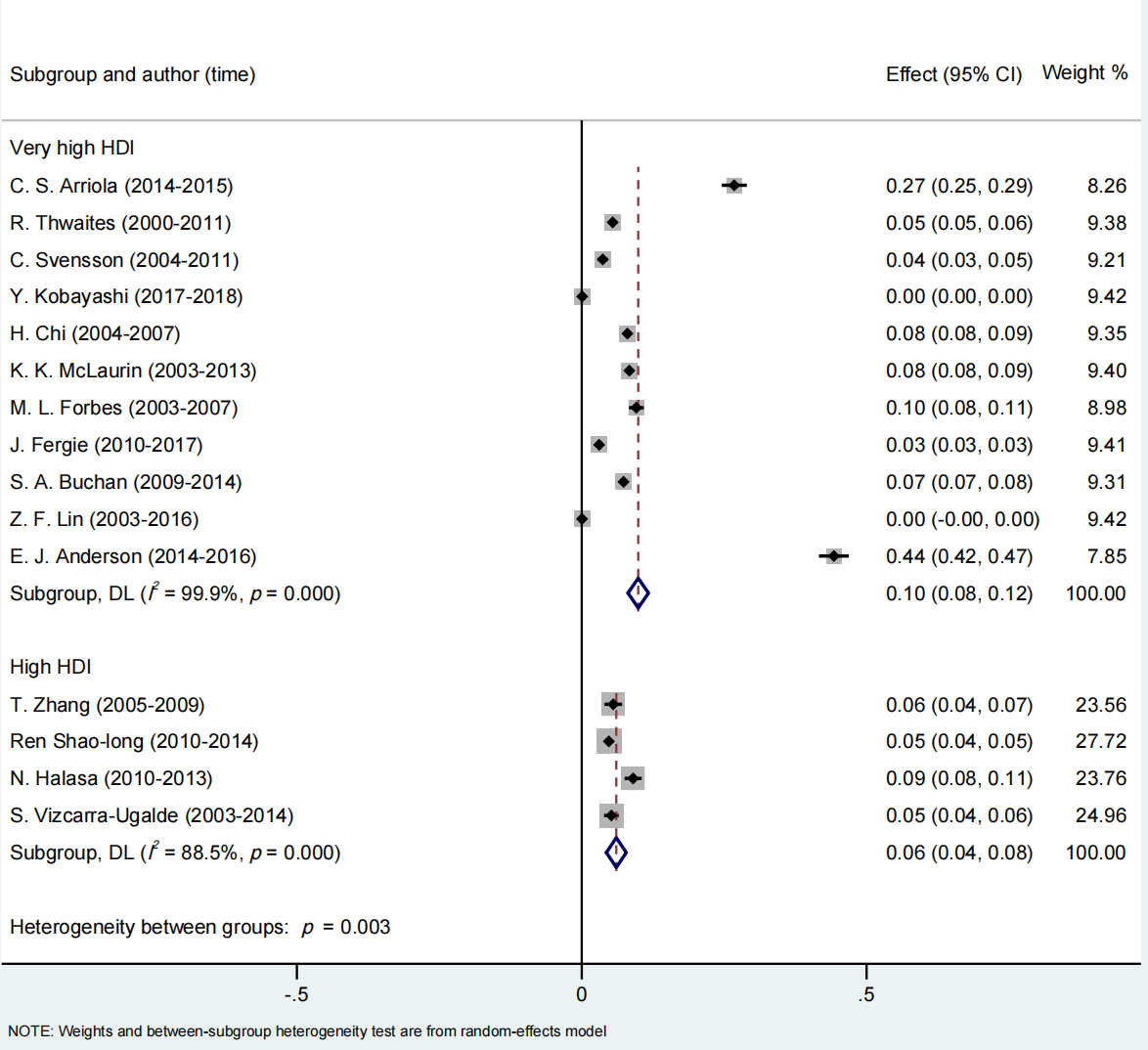 | 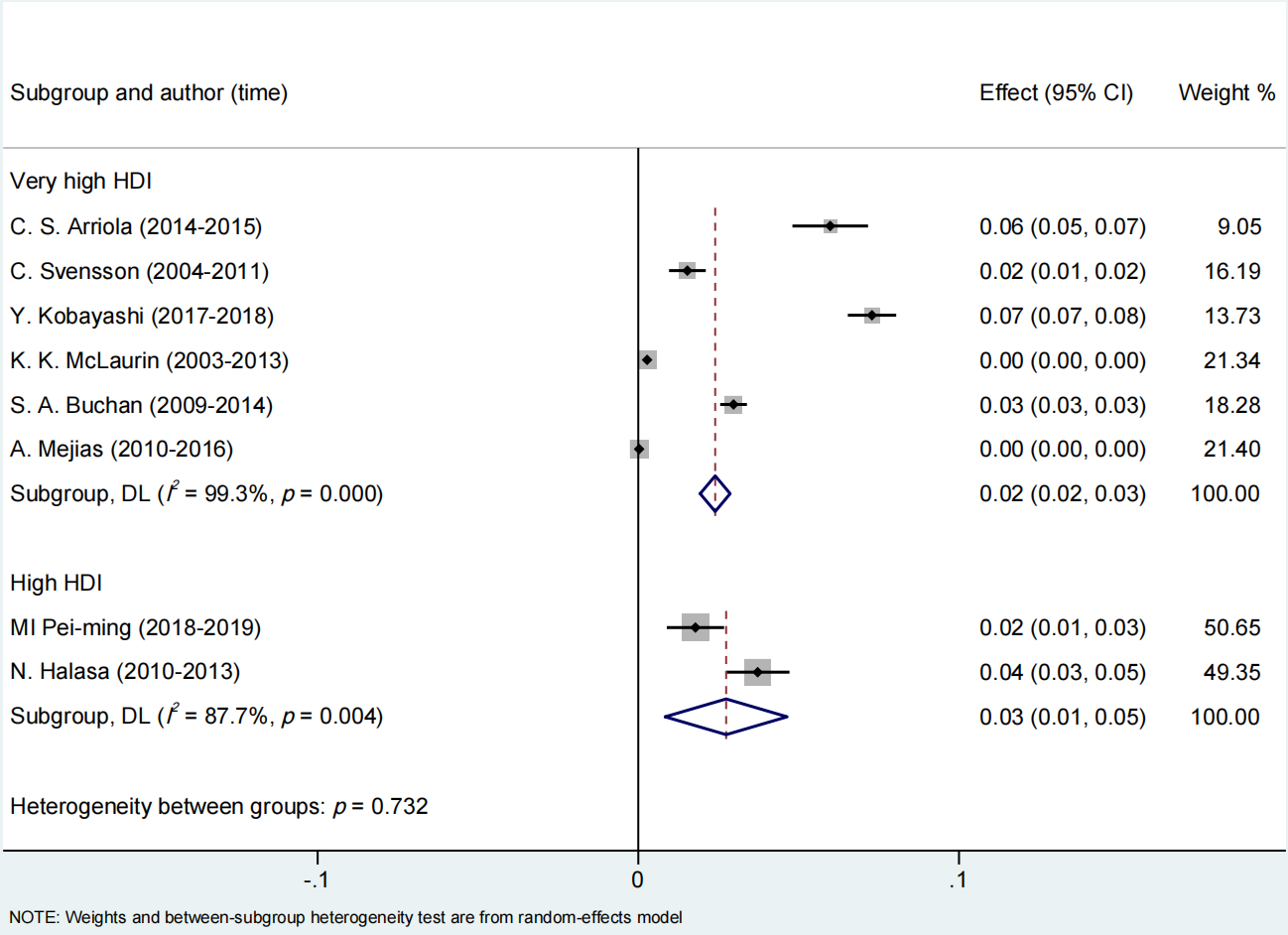 |
| --- | --- |
| **ICU admission** | **Mechanical ventilation** |
| 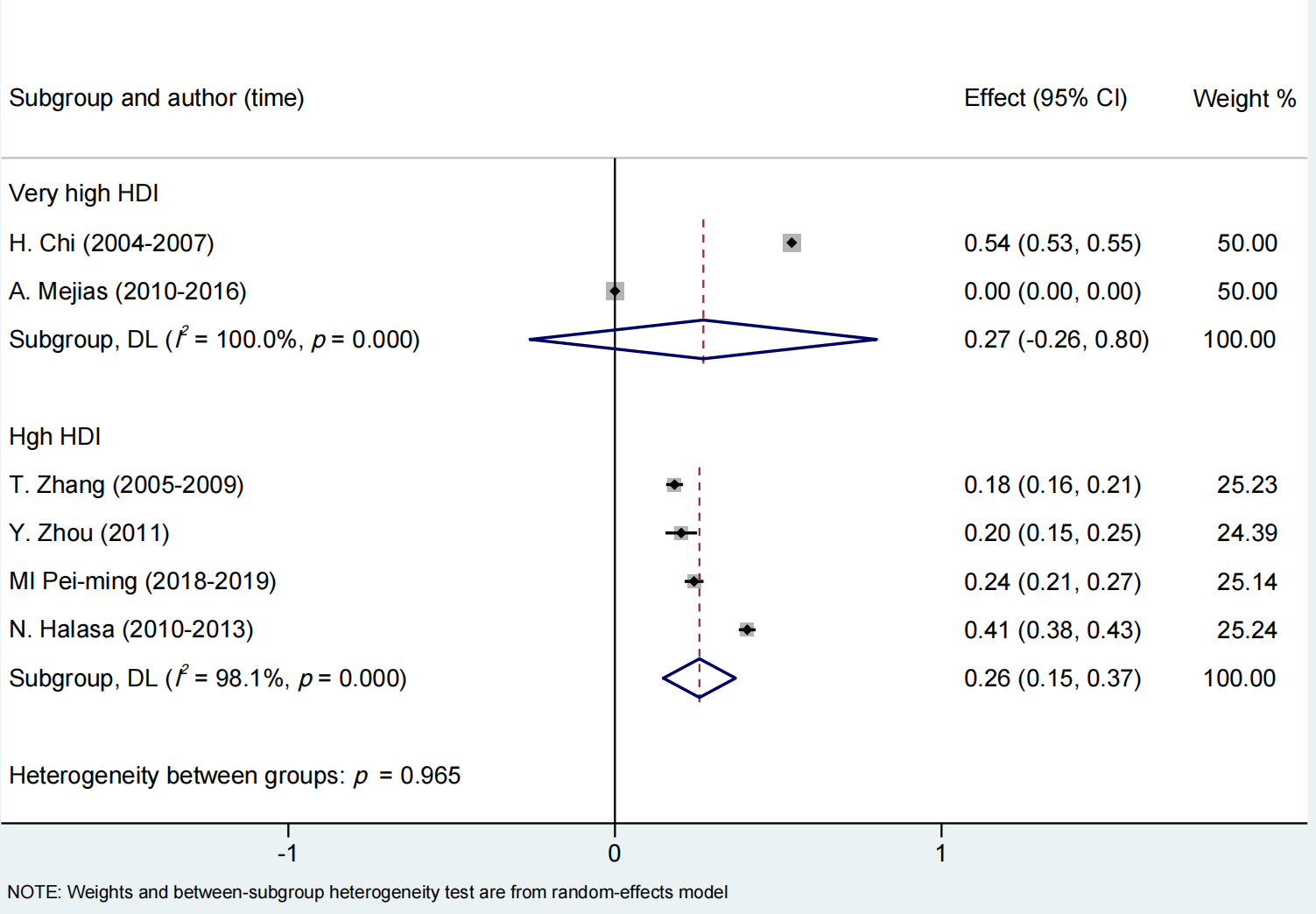 | 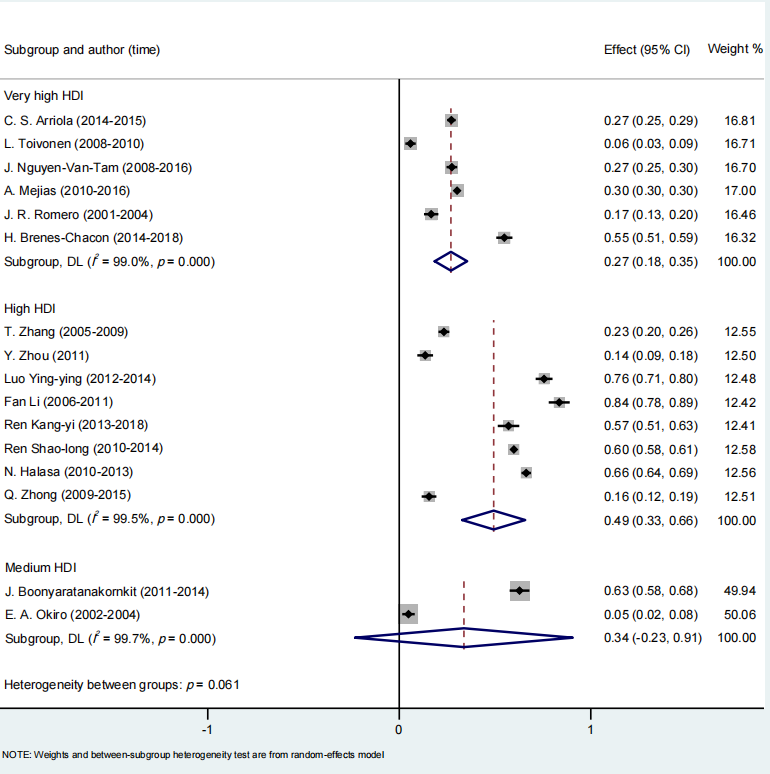 |
| **Oxygen supplementation** | **Wheezing** |
| 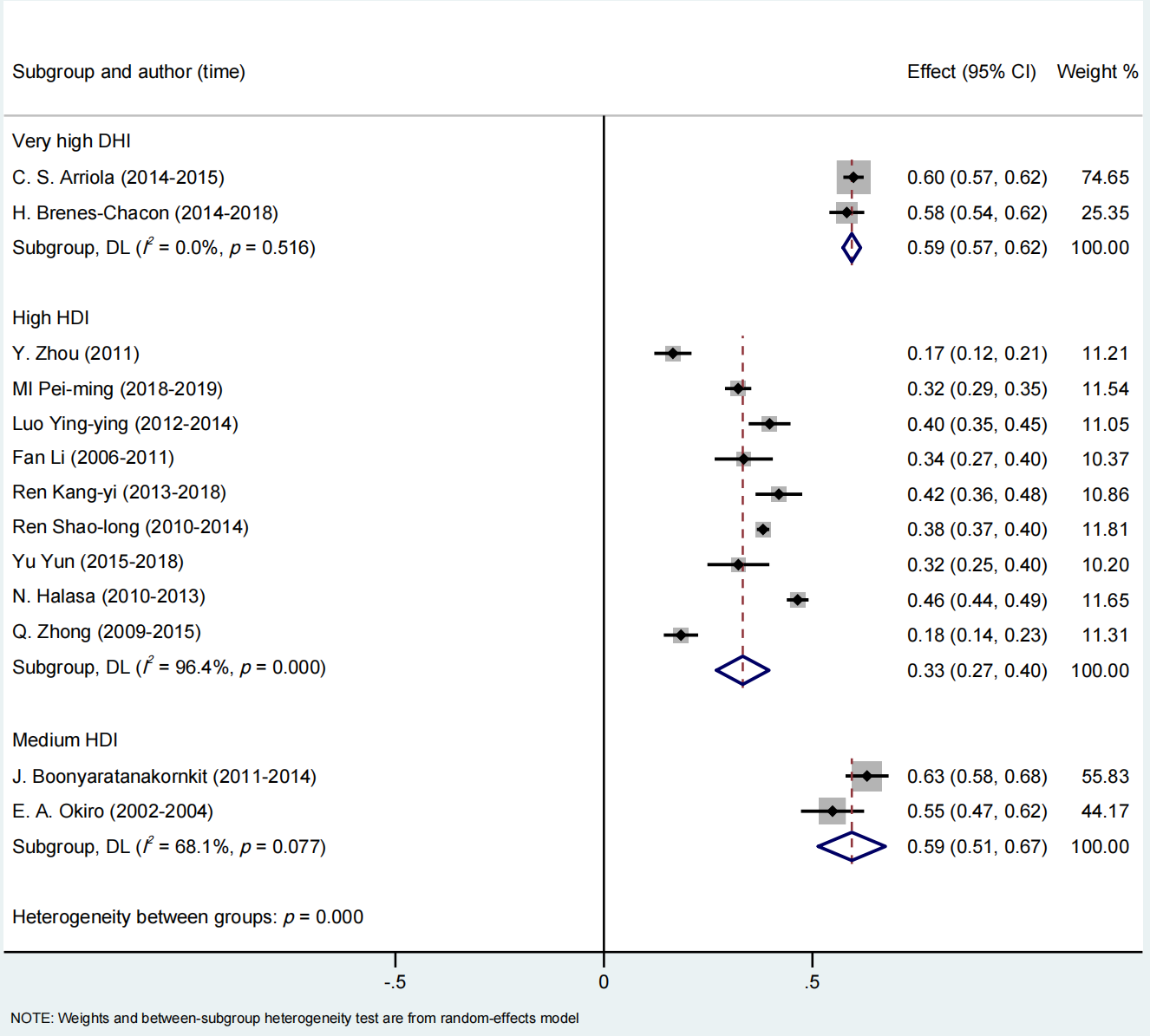 | 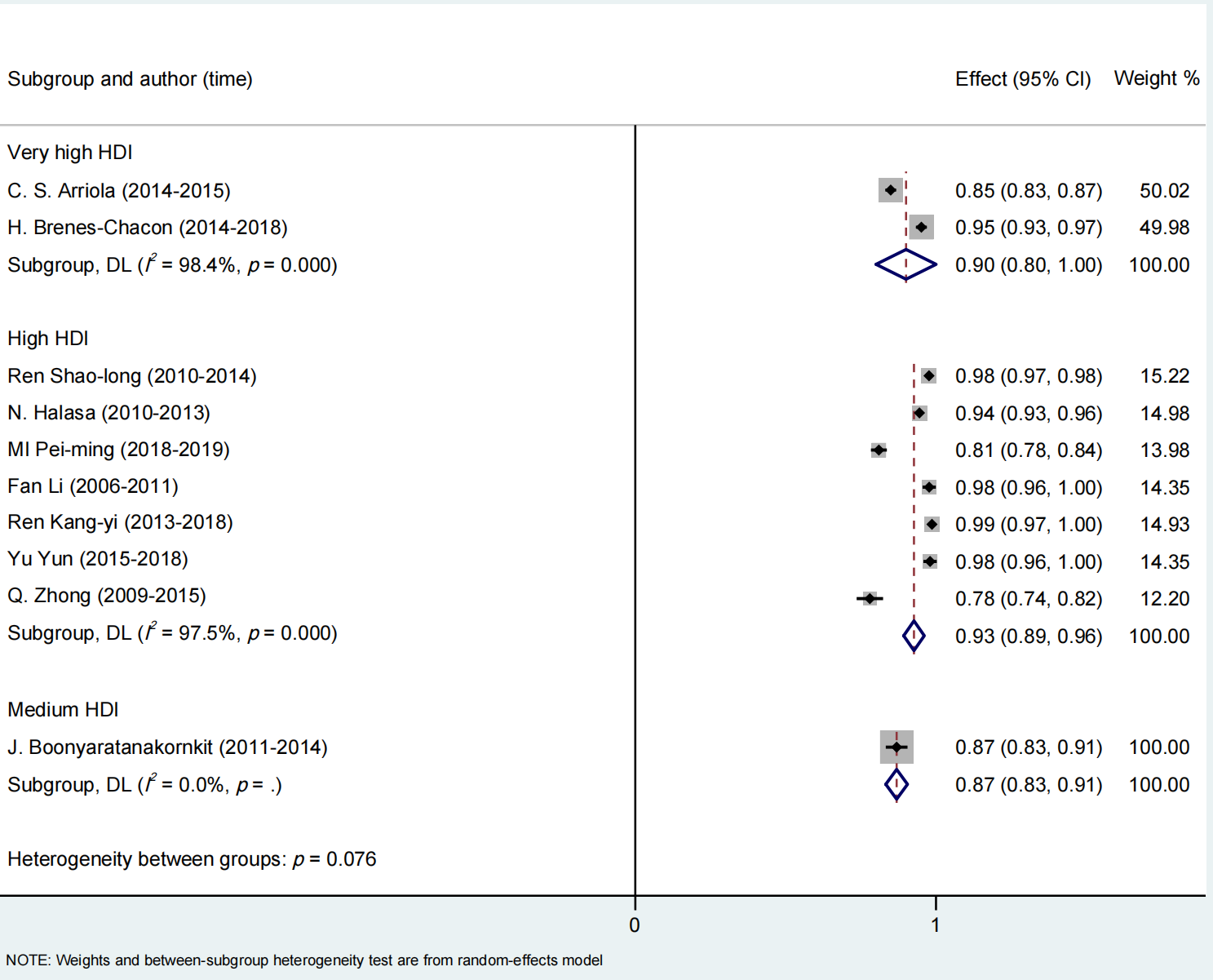 |
| **Fever** | **Cough** |
| 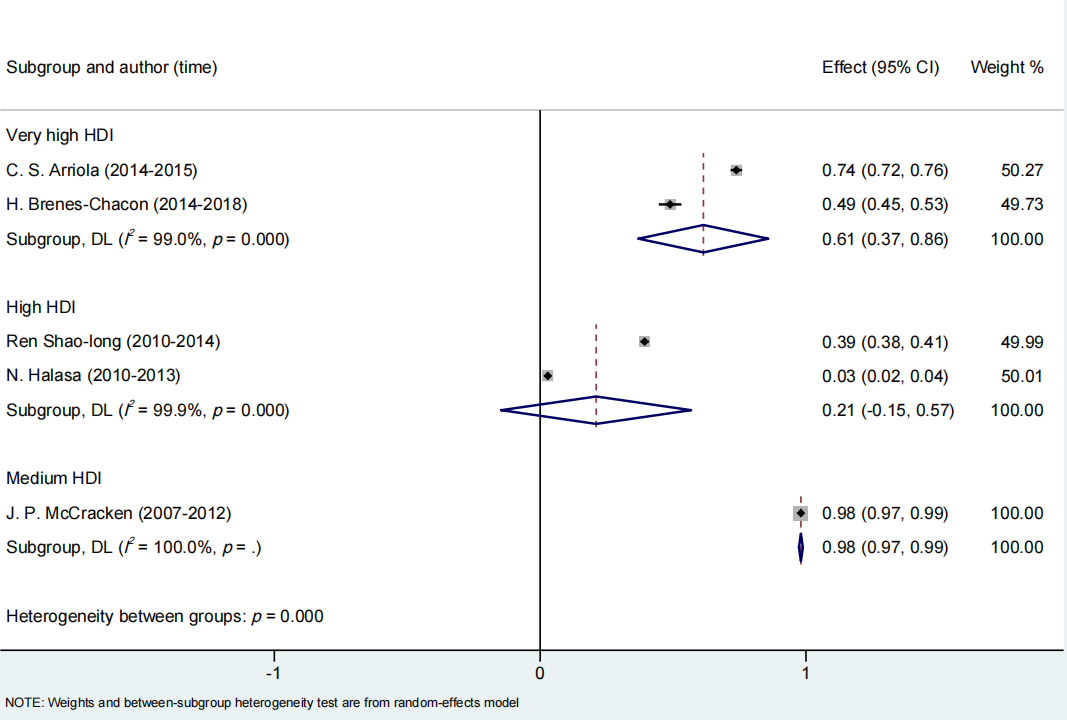 | 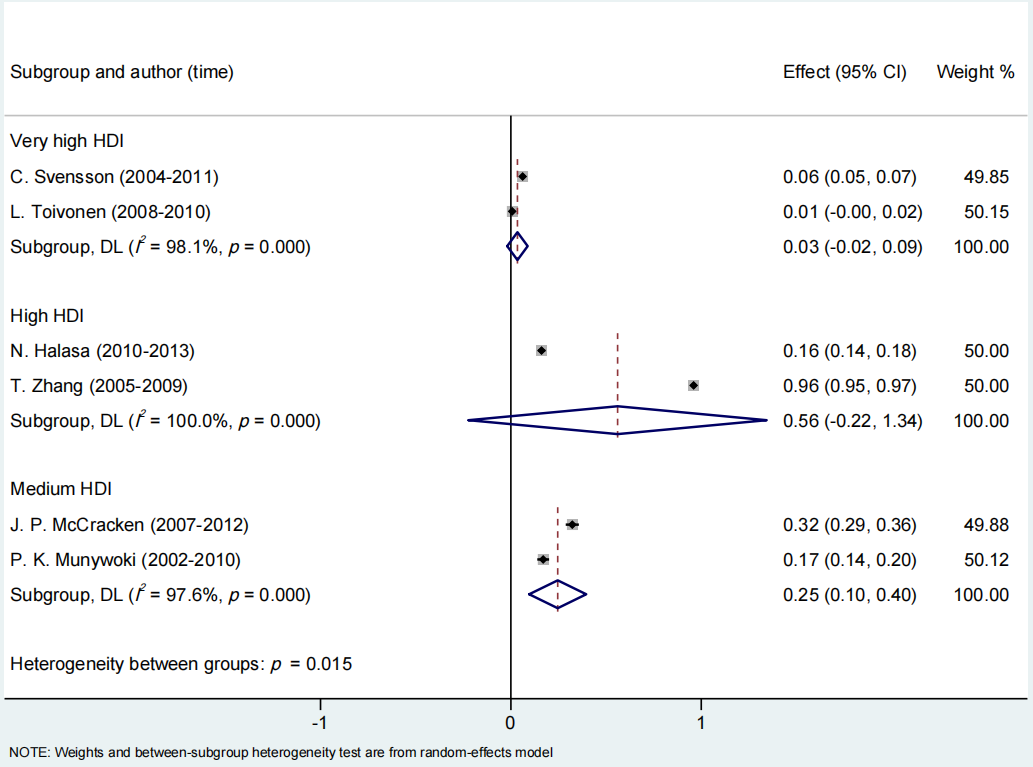 |
| **Rhinorrhea** | **Pneumonia** |
| 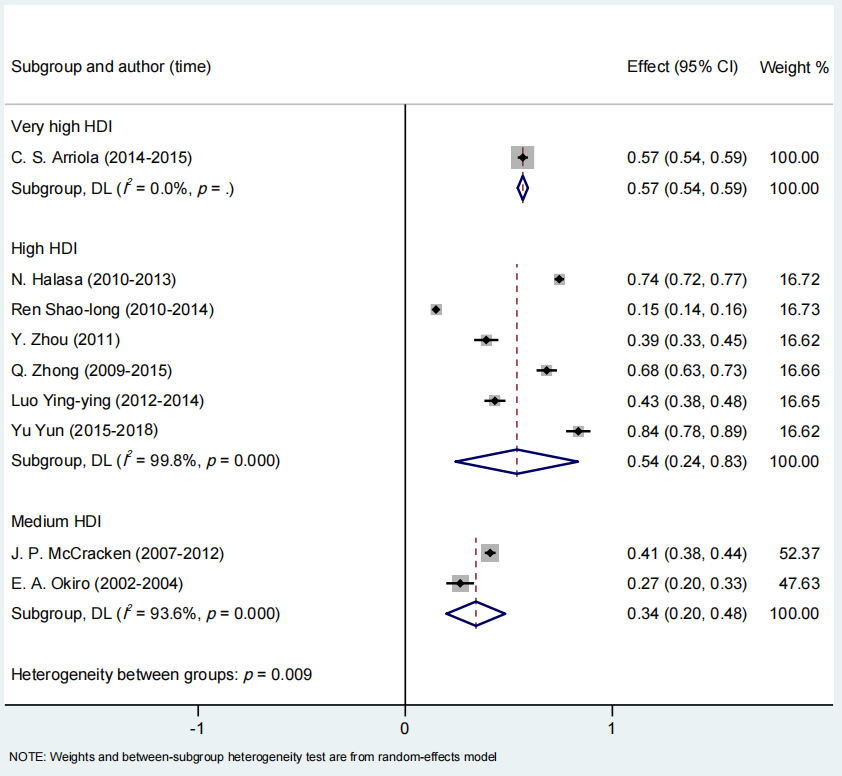 | 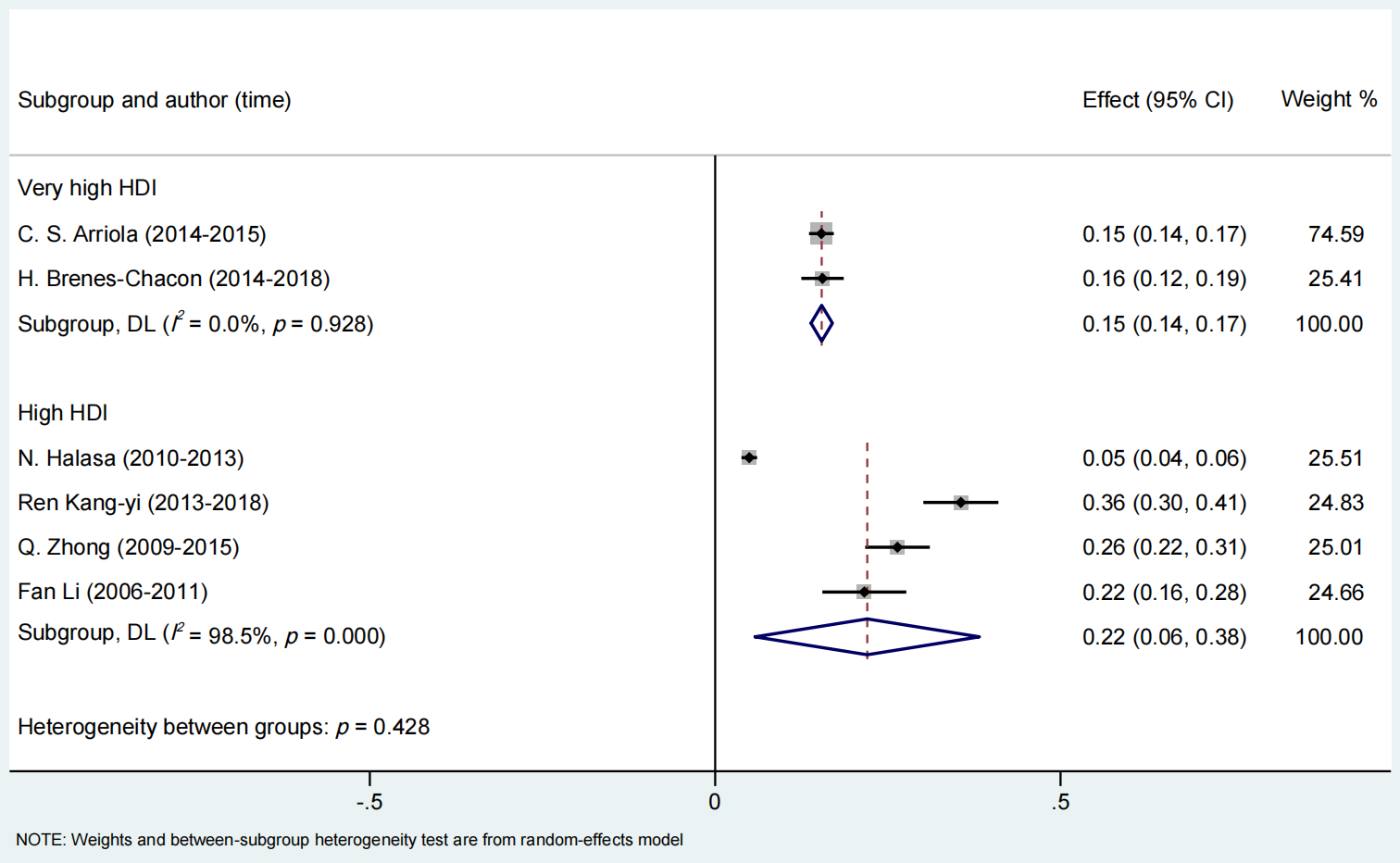 |
| **Shortness of breath** | **Diarrhea** |

**Supplementary figure 2. Common clinical manifestations of RSV infected children in different HDI level countries**

| 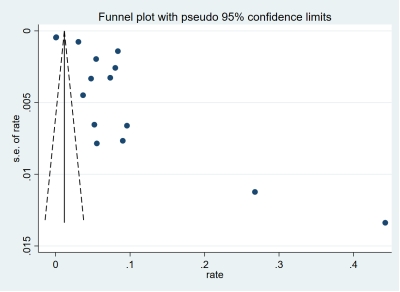 | 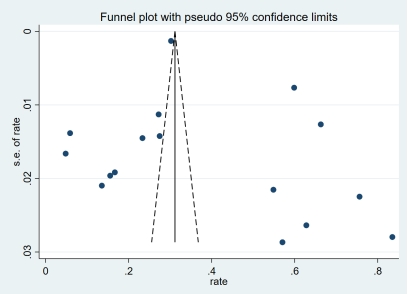 |
| --- | --- |
| **ICU admission (*P* = 0.001)** | **Wheezing (*P* = 0.314)** |
| 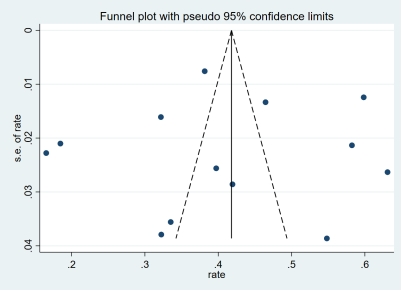 | 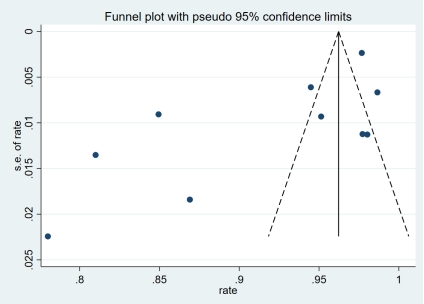 |
| **Fever (*P* = 0.915)** | **Cough (*P* = 0.030)** |

**Supplementary figure 3.** **Publication bias analyzed by funnel plot**

| 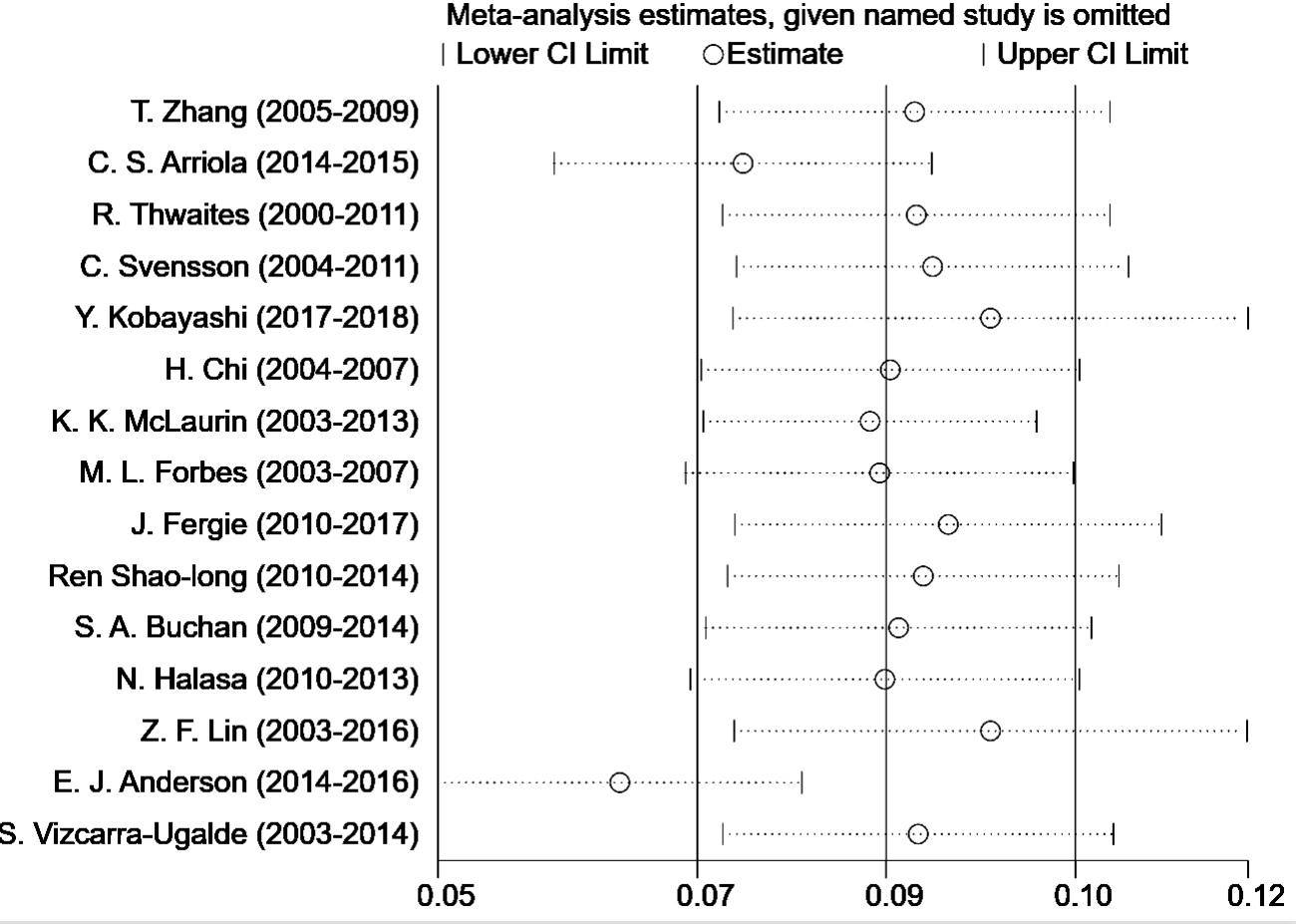 | 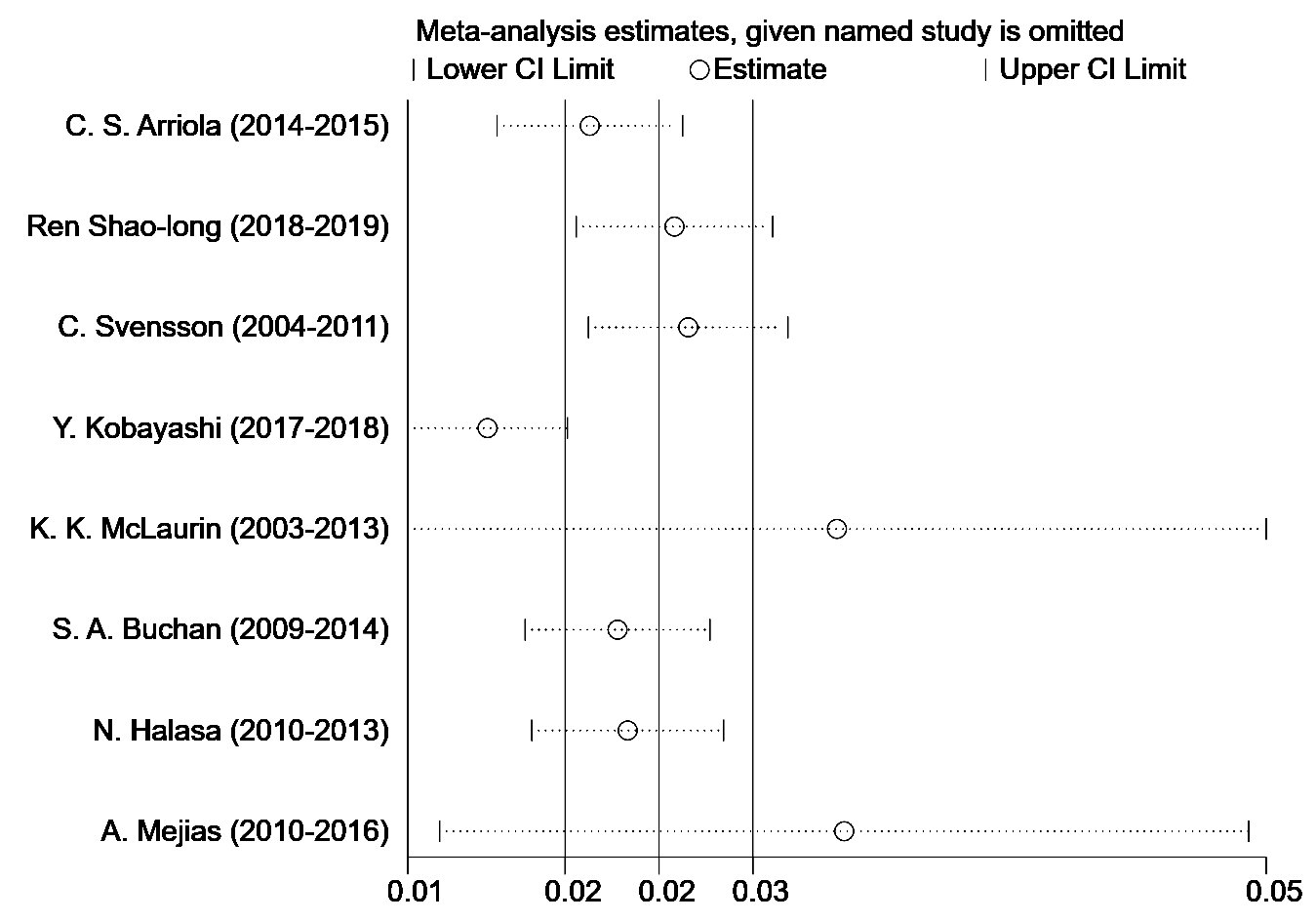 |
| --- | --- |
| **ICU admission** | Mechanical ventilation |
| 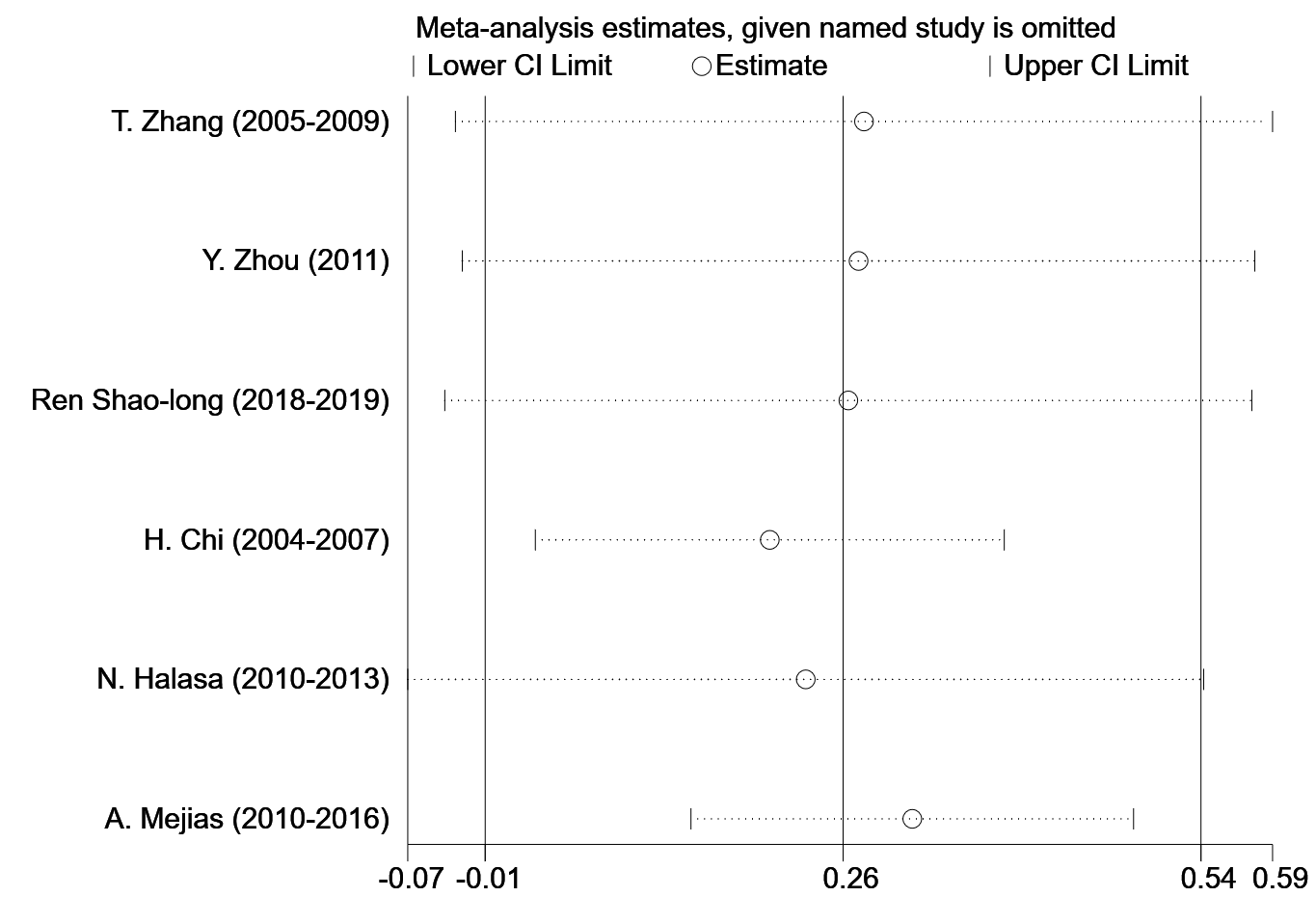 | 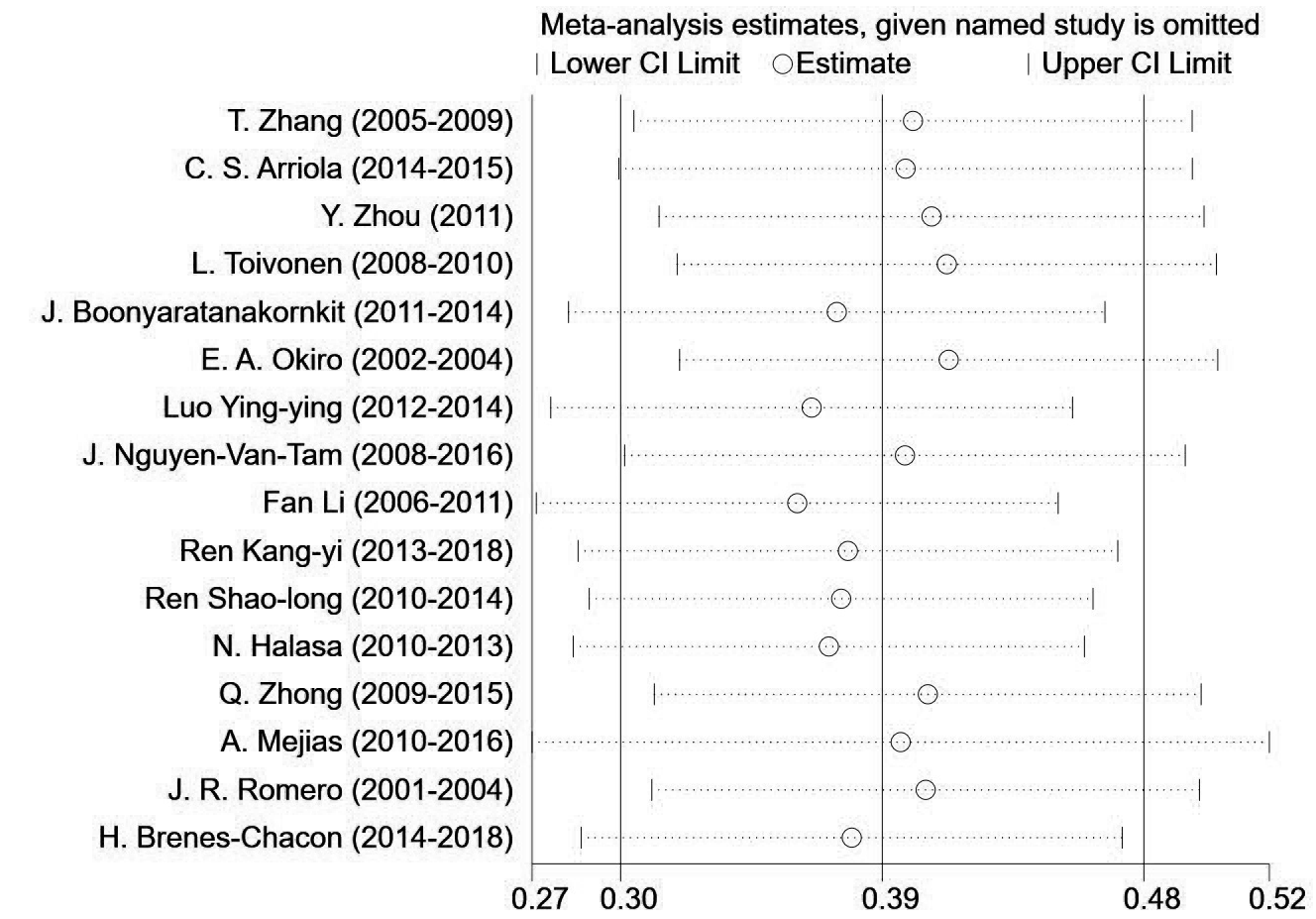 |
| **Oxygen supplementary** | **Wheezing** |
| 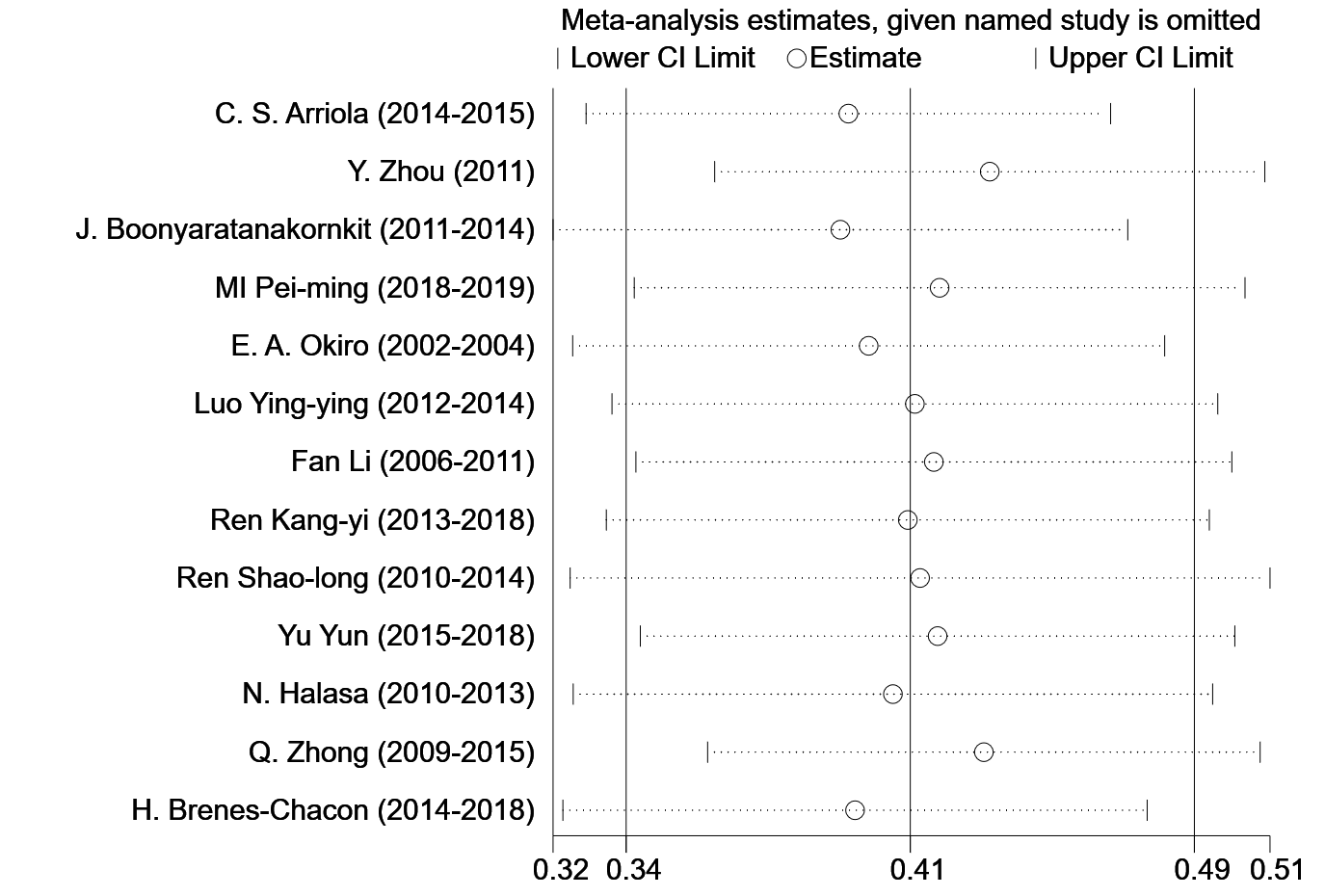 | 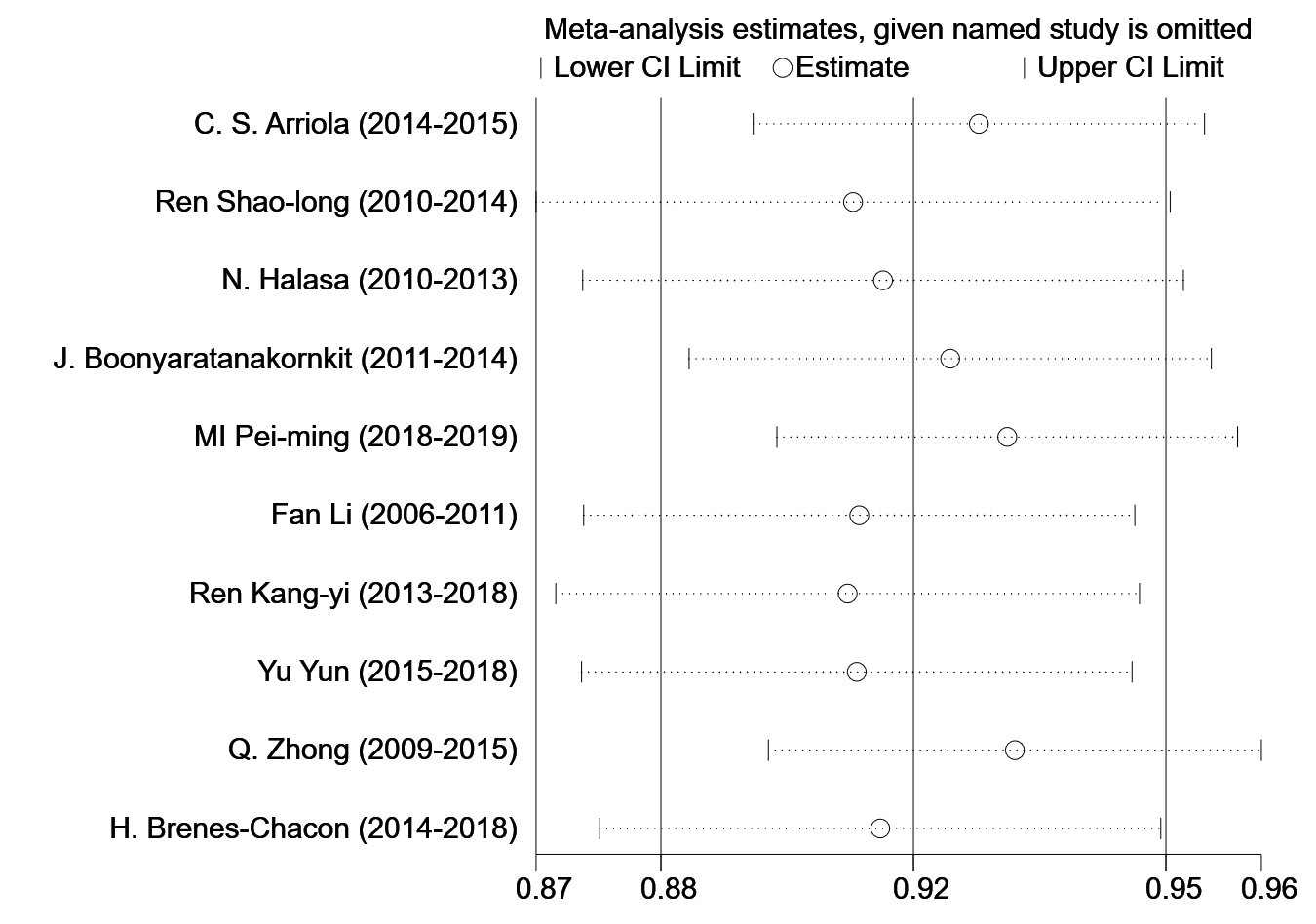 |
| **Fever** | **Cough** |
| 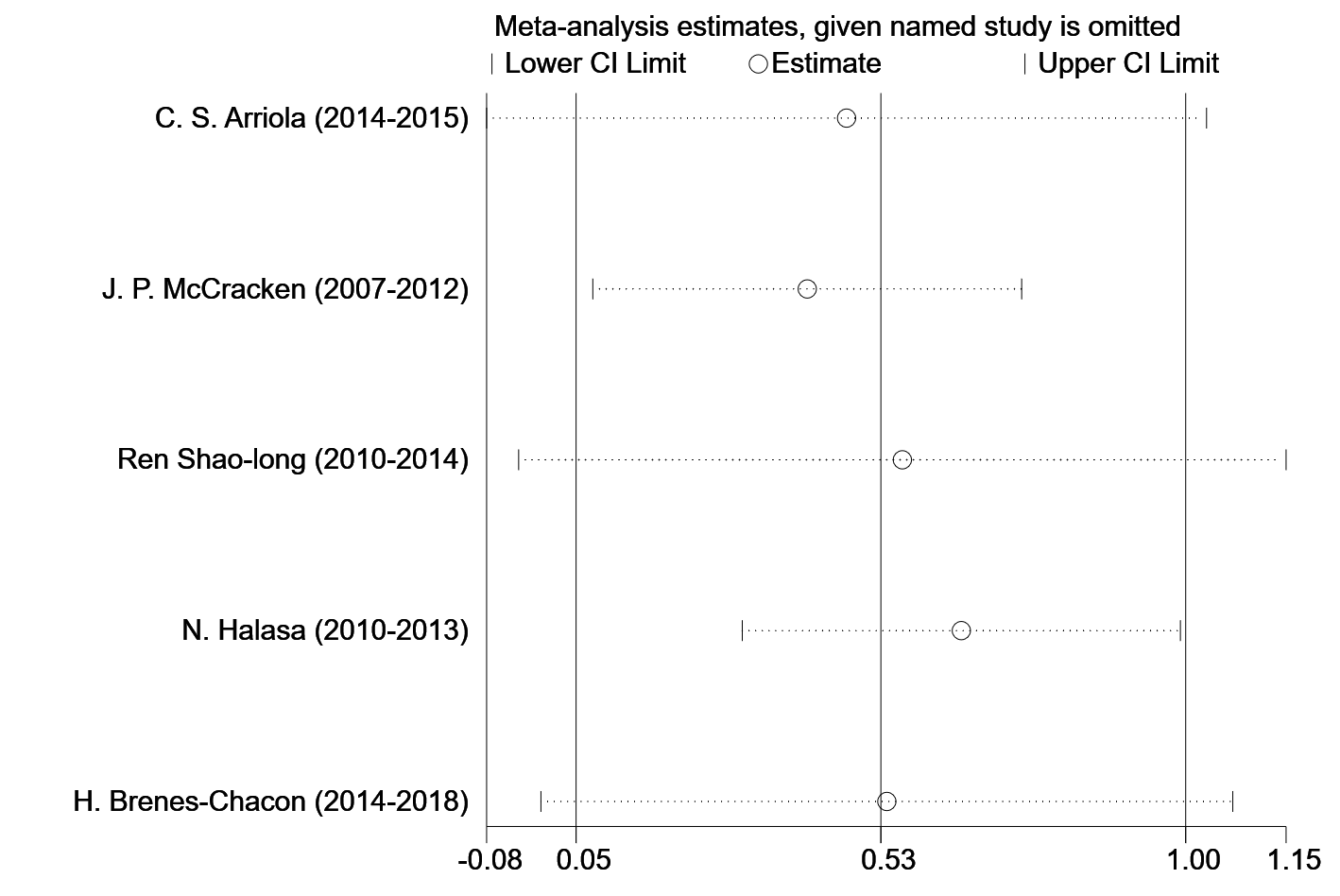 | 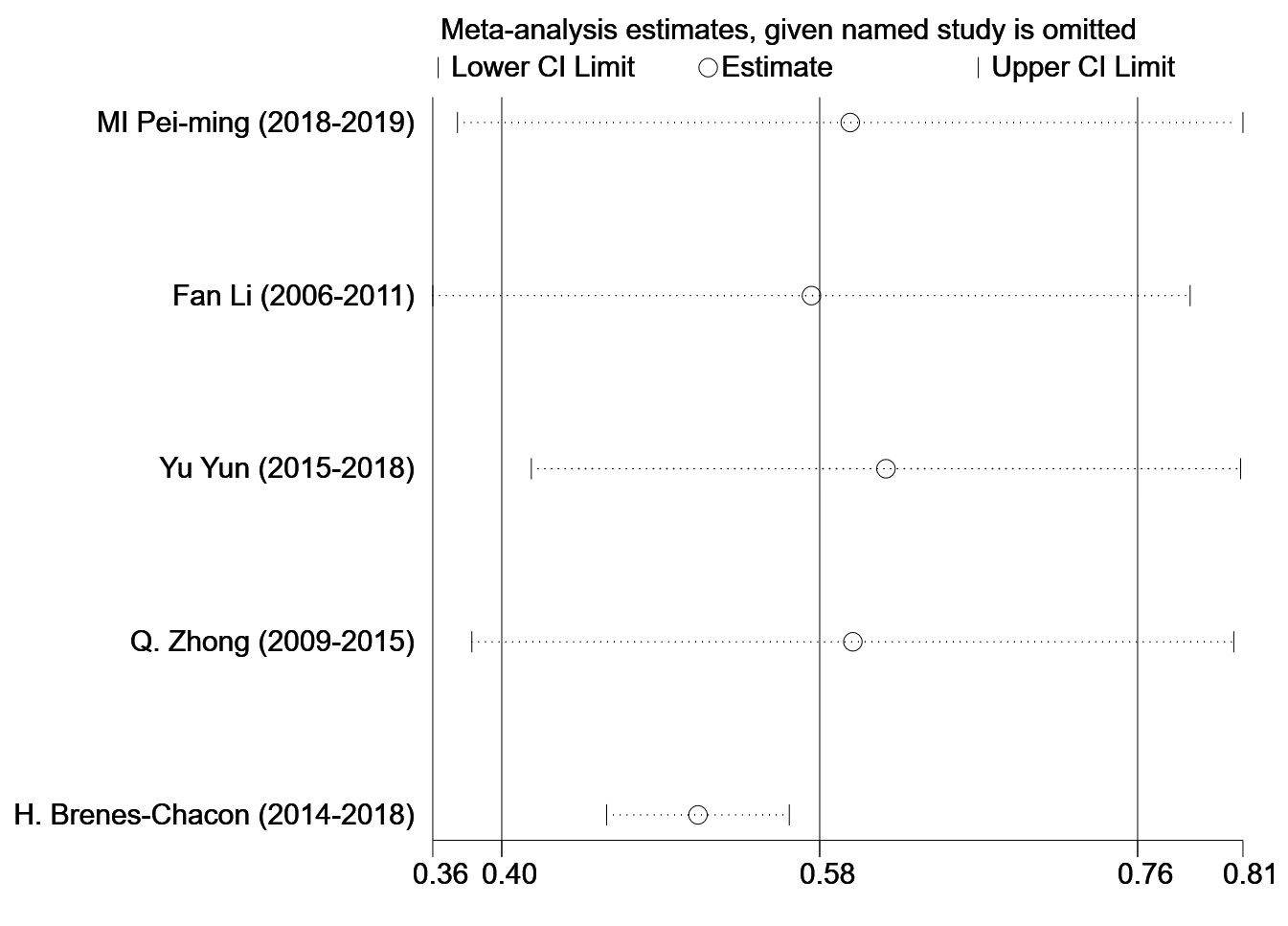 |
| **Rhinorrhea** | **Nasal congestion** |
| 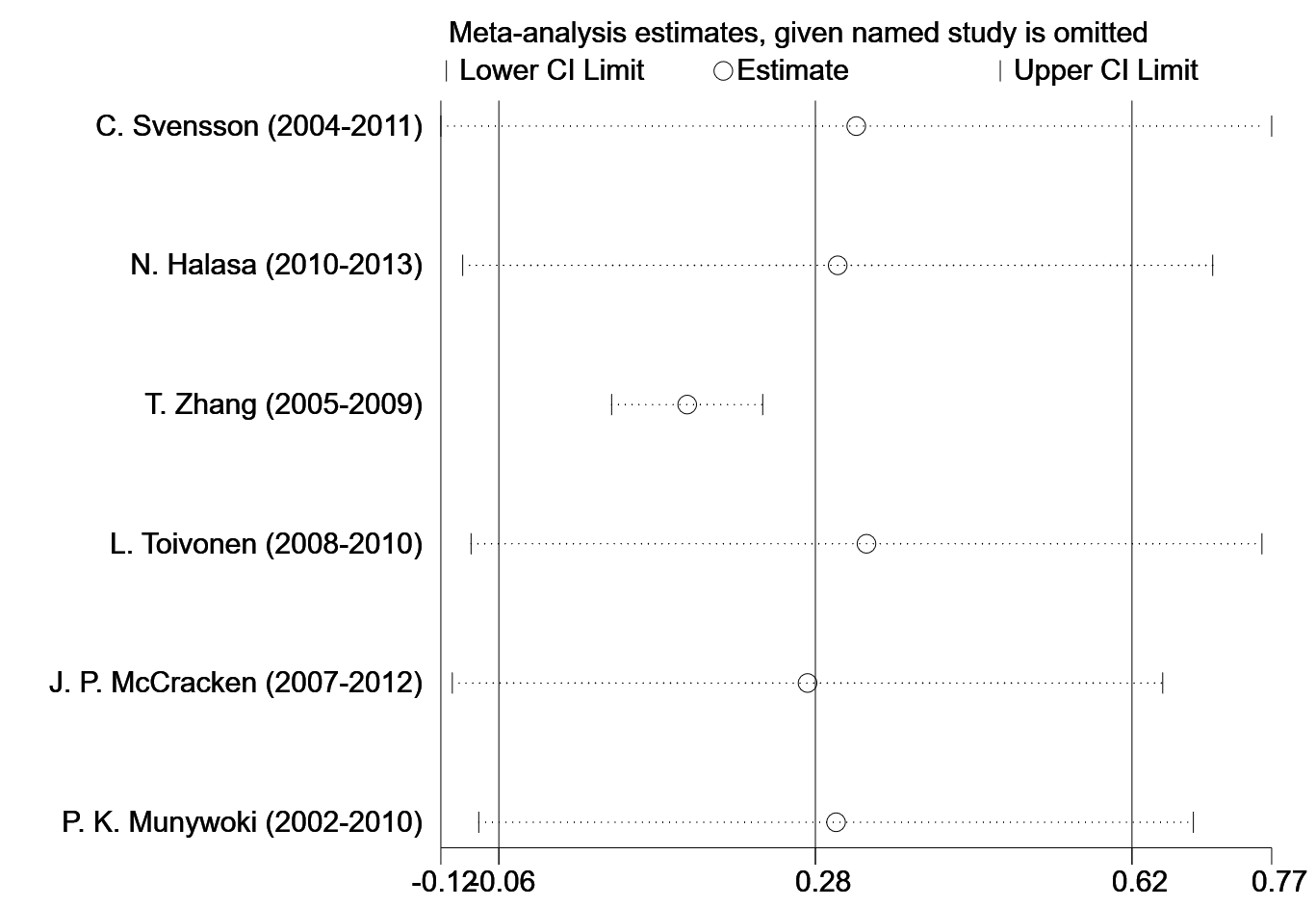 | 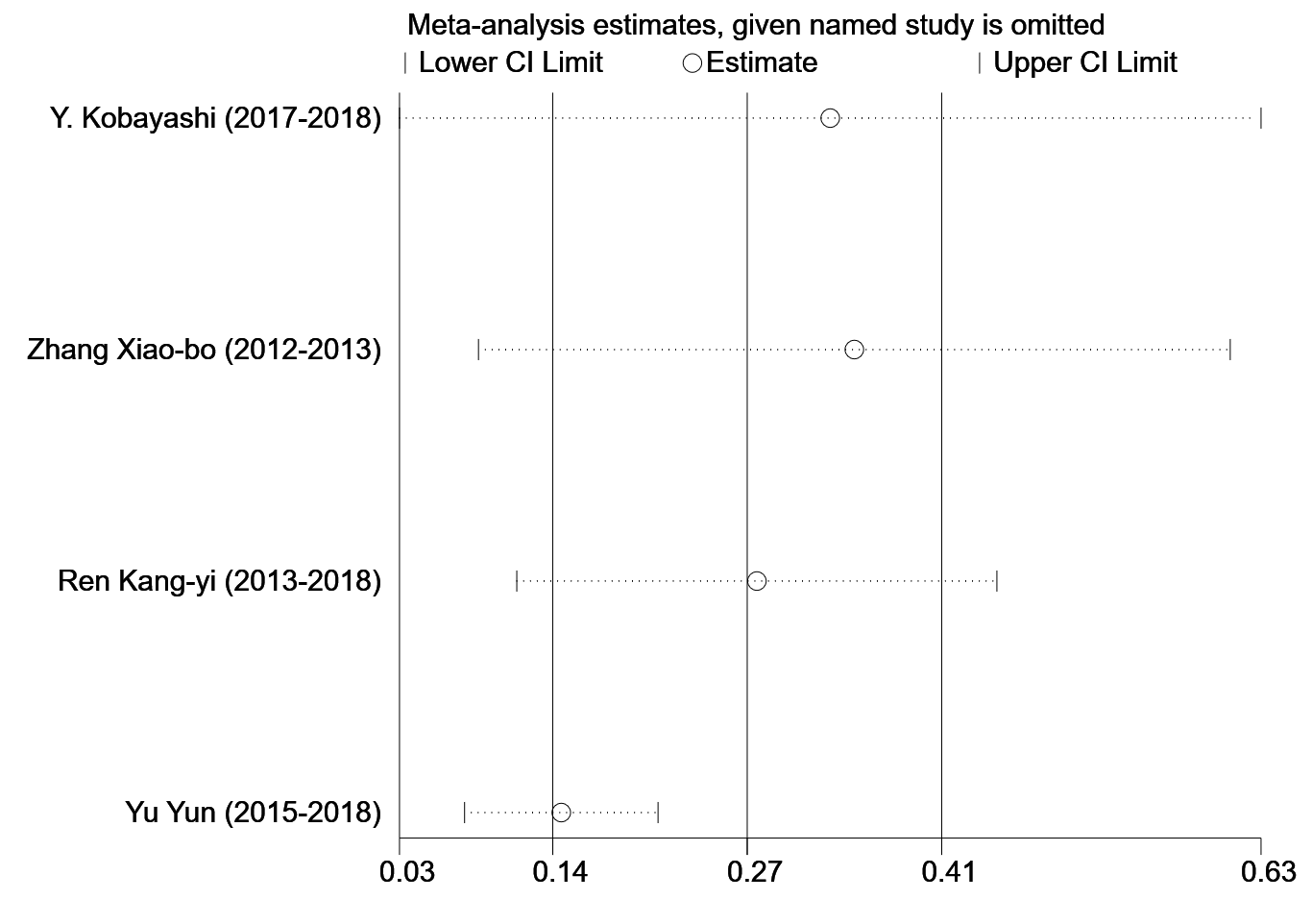 |
| **Pneumonia** | **Respiratory failure** |
| 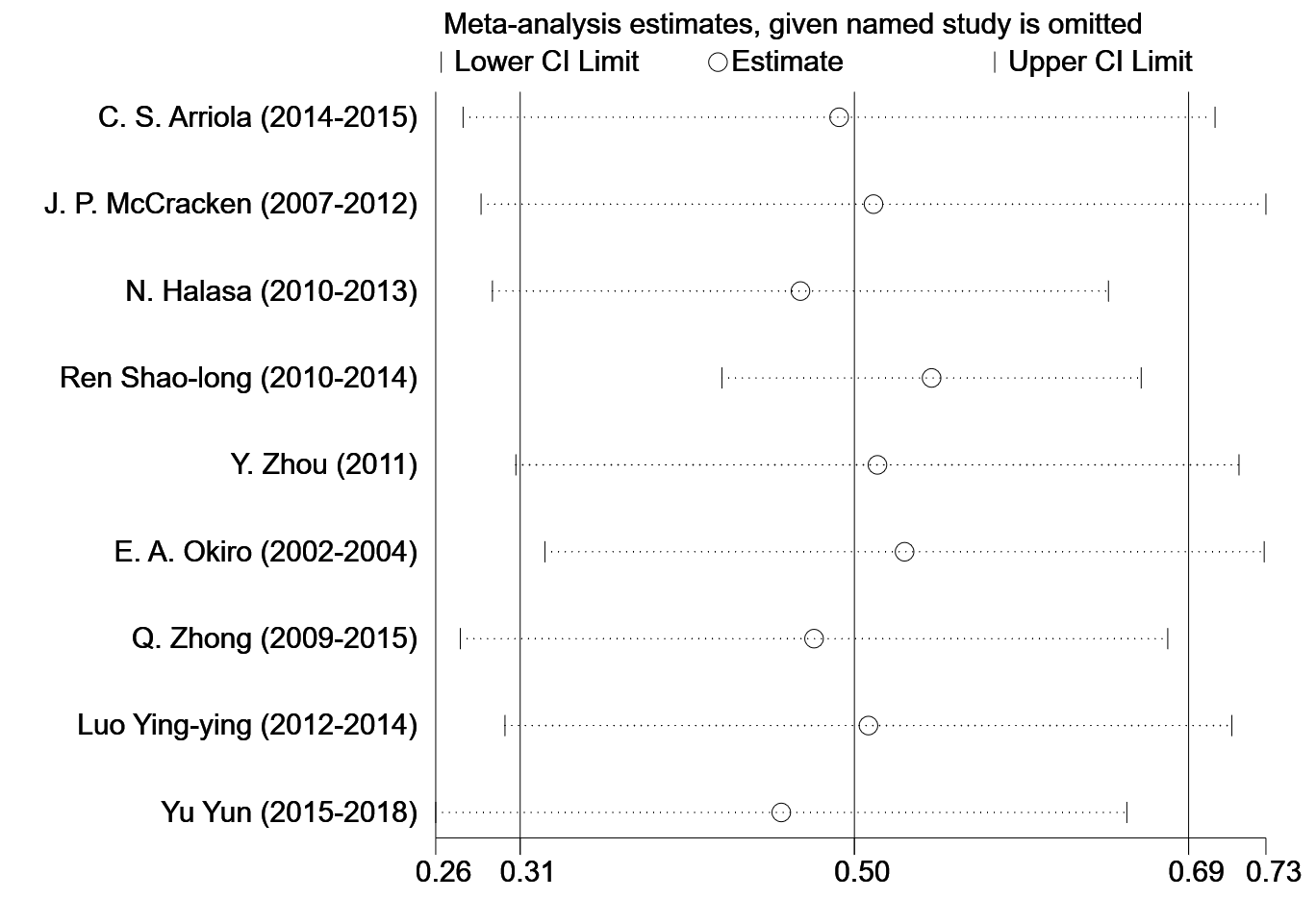 | 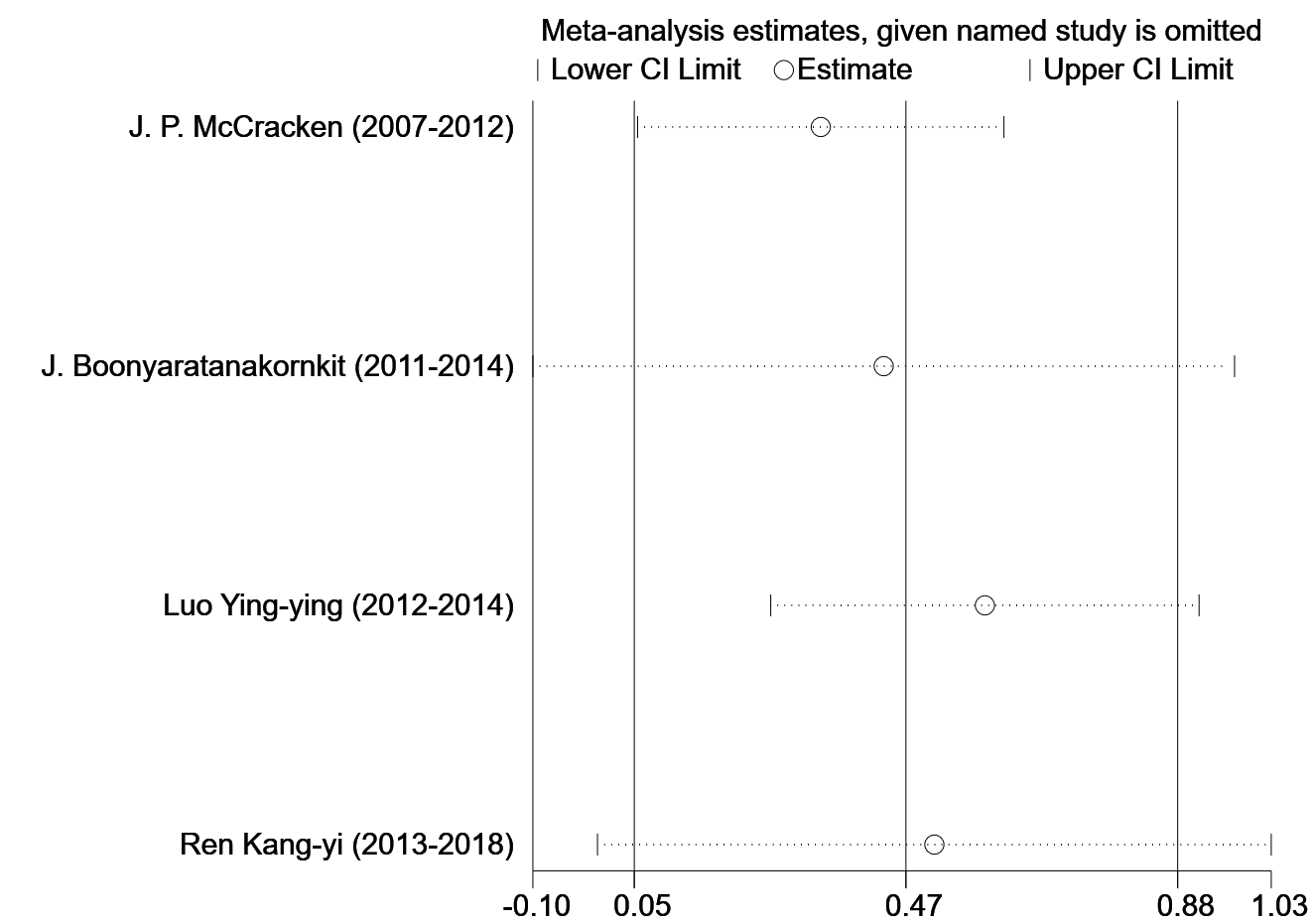 |
| **Shortness of breath** | **Dyspnea** |
| 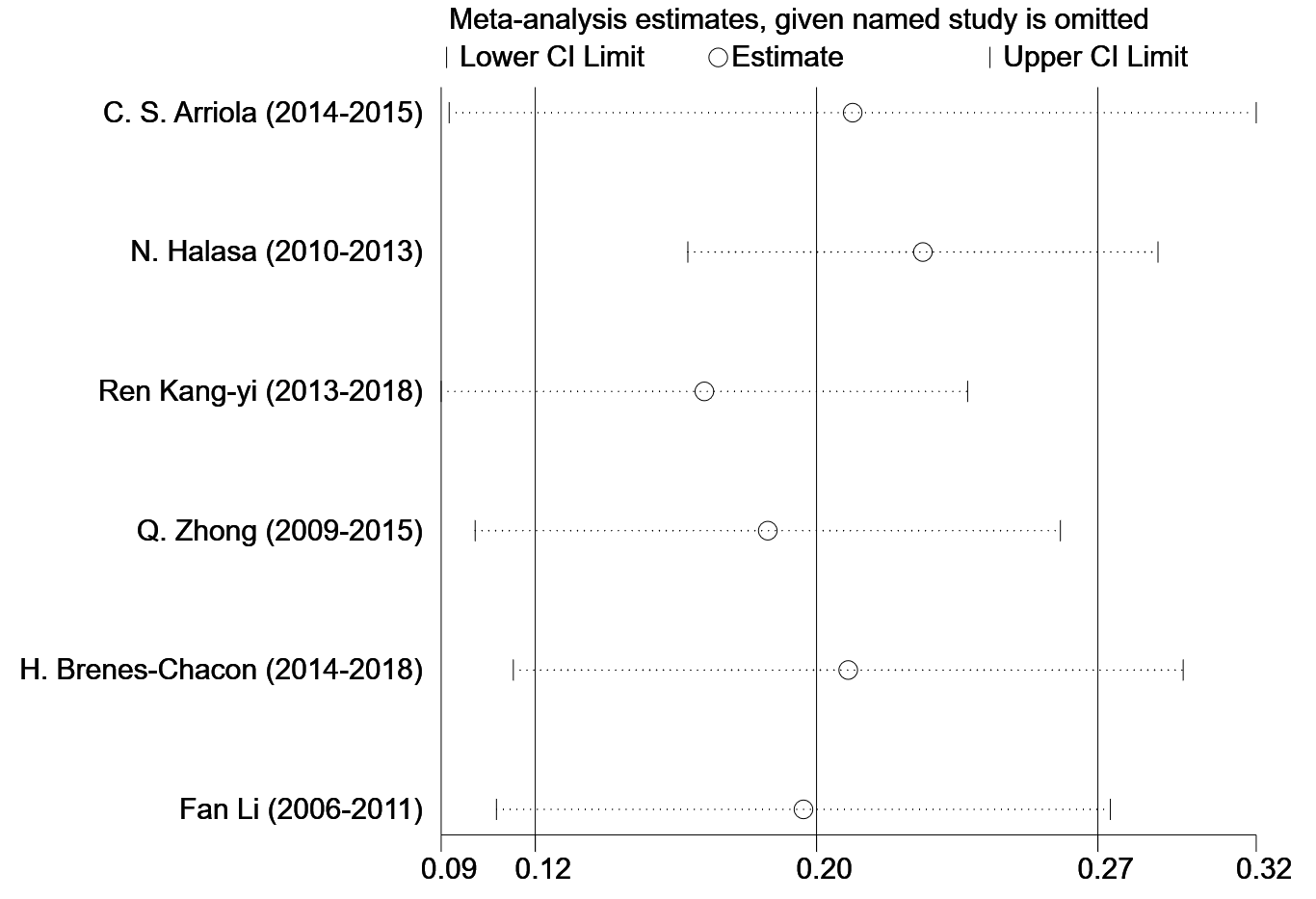 | 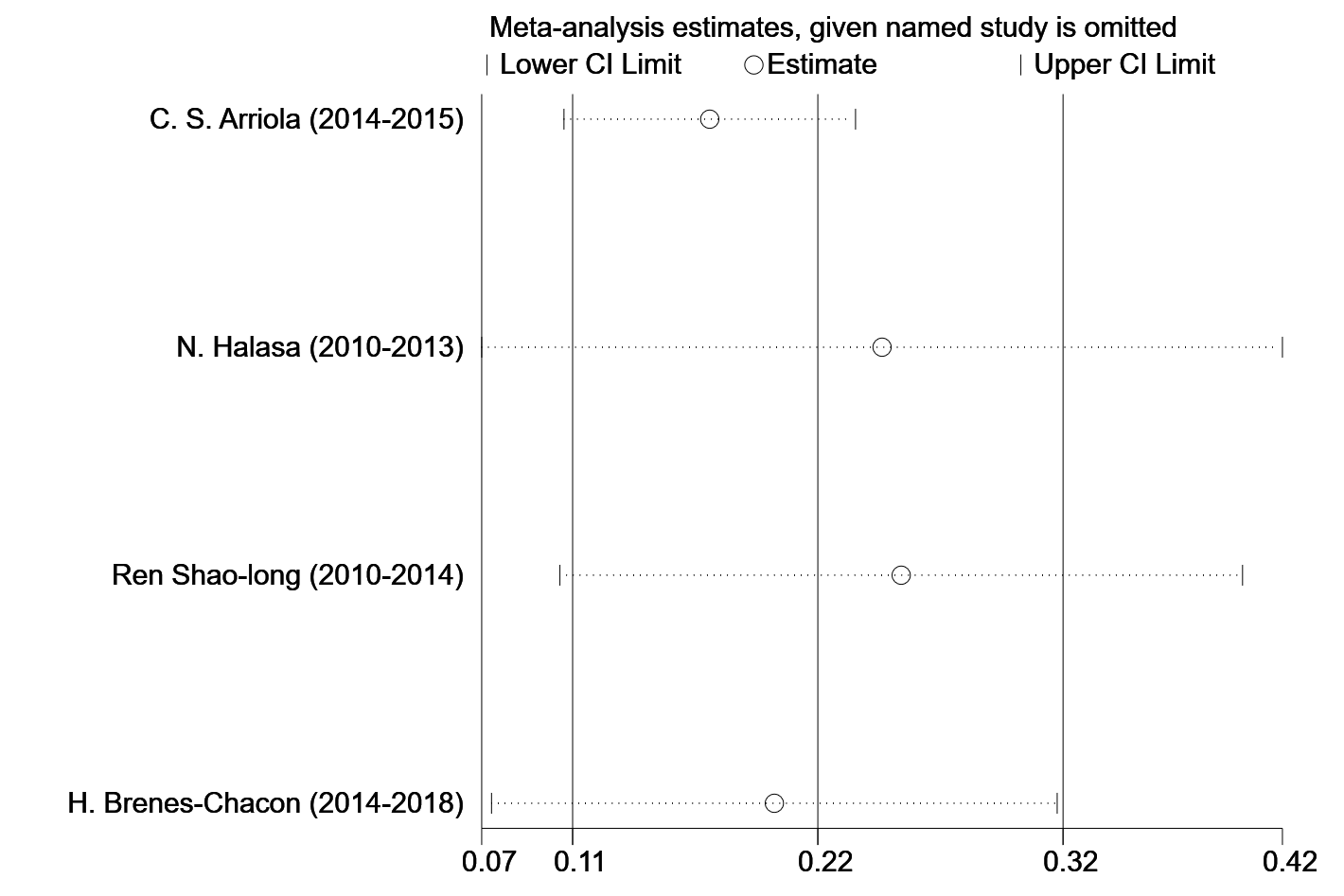 |
| **Diarrhea** | **Vomiting** |
| 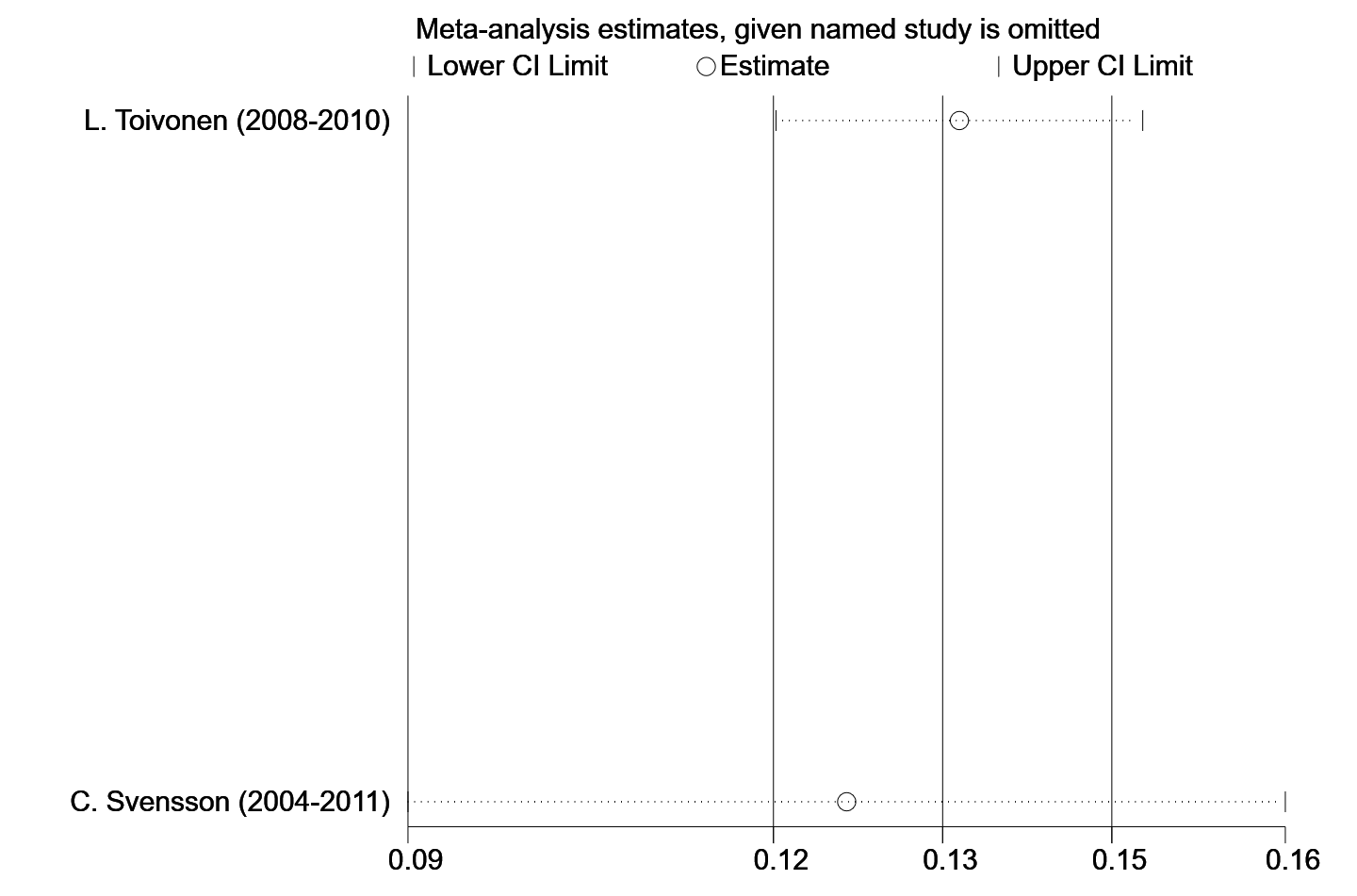 | 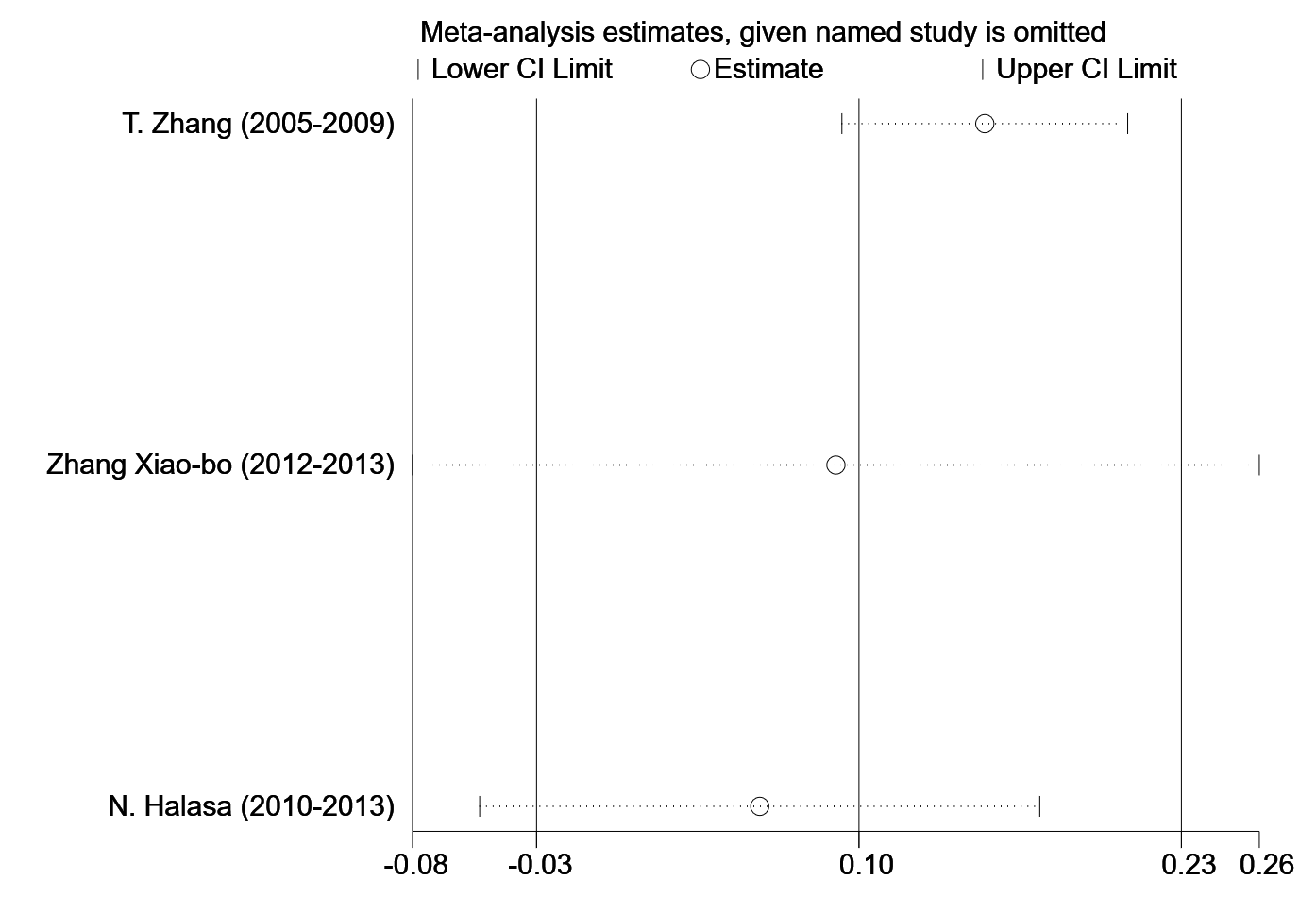 |
| **Otitis media** | **septice** |
| 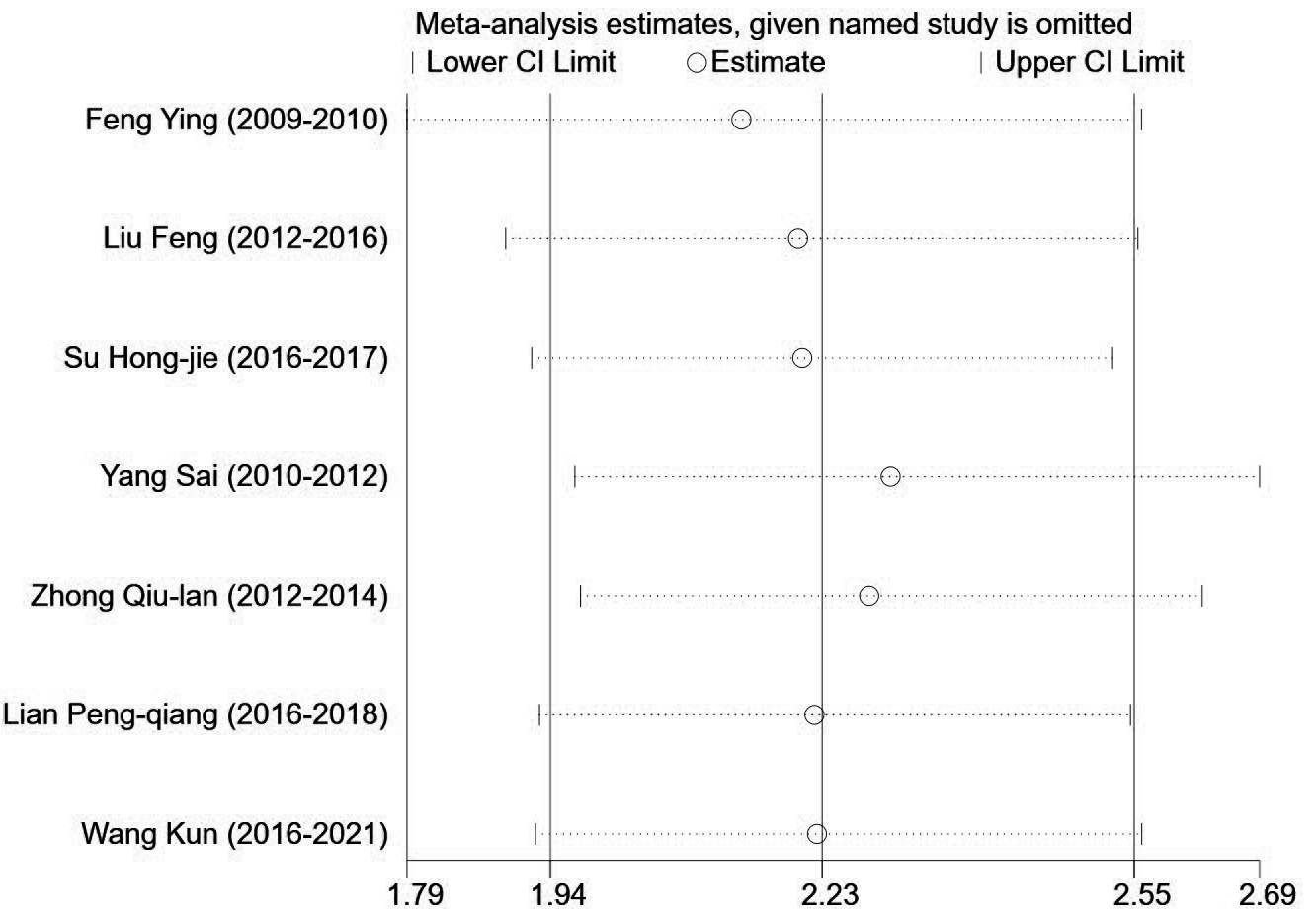 | 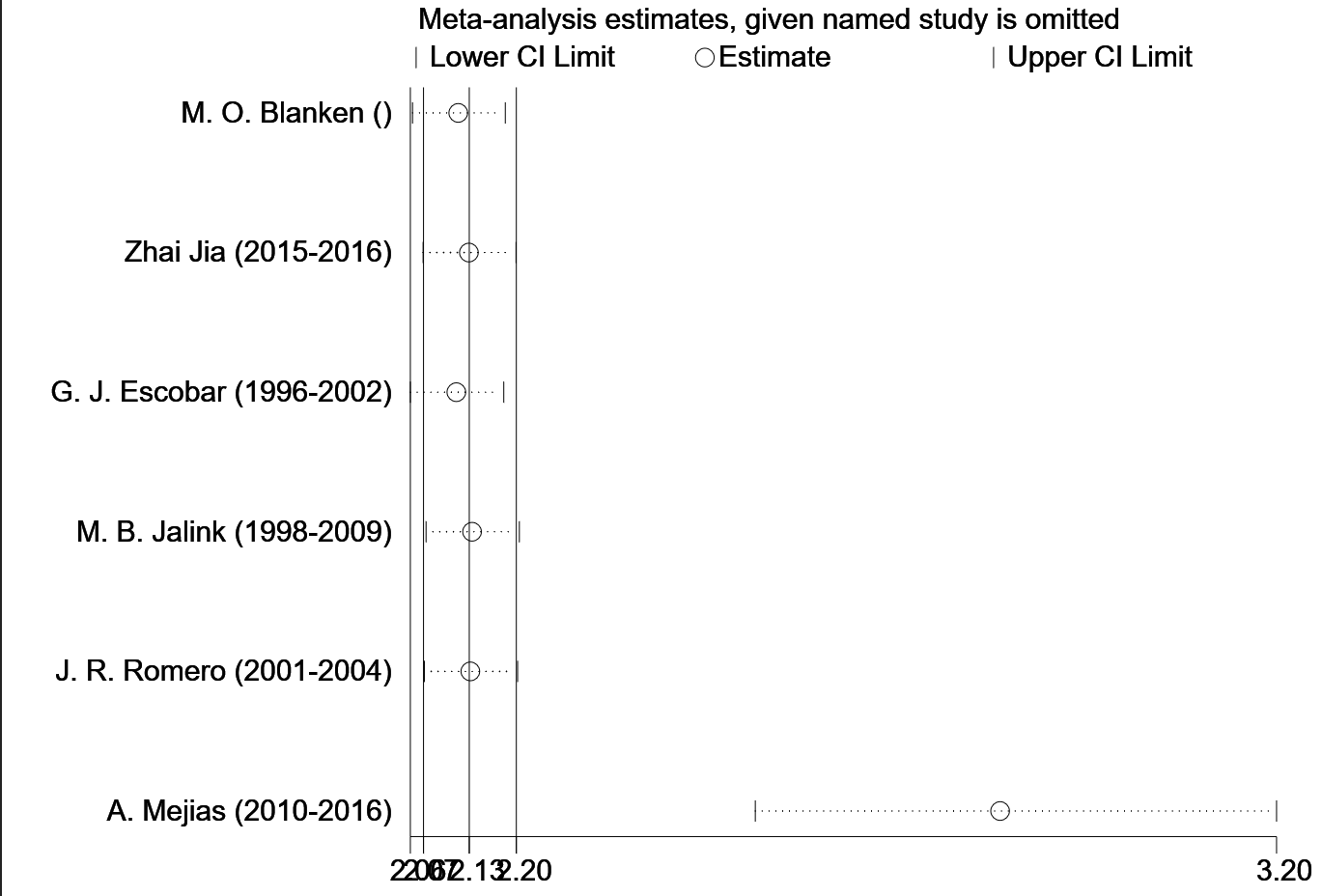 |
| **Occurrence of wheezing** | **Subsequent development of recurrent wheeze illness** |

**Supplementary figure 4.** **Sensitivity analysis**

| **Section and Topic** | **Item #** | **Checklist item** | **Location where item is reported** |
| --- | --- | --- | --- |
| **TITLE** | | |  |
| Title | 1 | Identify the report as a systematic review. | 1 |
| **ABSTRACT** | | |  |
| Abstract | 2 | See the PRISMA 2020 for Abstracts checklist. | 2 |
| **INTRODUCTION** | | |  |
| Rationale | 3 | Describe the rationale for the review in the context of existing knowledge. | 3-4 |
| Objectives | 4 | Provide an explicit statement of the objective(s) or question(s) the review addresses. | 3-4 |
| **METHODS** | | |  |
| Eligibility criteria | 5 | Specify the inclusion and exclusion criteria for the review and how studies were grouped for the syntheses. | 5, Table 1 |
| Information sources | 6 | Specify all databases, registers, websites, organisations, reference lists and other sources searched or consulted to identify studies. Specify the date when each source was last searched or consulted. | 4 |
| Search strategy | 7 | Present the full search strategies for all databases, registers and websites, including any filters and limits used. | Supplementary Table 1 |
| Selection process | 8 | Specify the methods used to decide whether a study met the inclusion criteria of the review, including how many reviewers screened each record and each report retrieved, whether they worked independently, and if applicable, details of automation tools used in the process. | 4-4 |
| Data collection process | 9 | Specify the methods used to collect data from reports, including how many reviewers collected data from each report, whether they worked independently, any processes for obtaining or confirming data from study investigators, and if applicable, details of automation tools used in the process. | 5-6 |
| Data items | 10a | List and define all outcomes for which data were sought. Specify whether all results that were compatible with each outcome domain in each study were sought (e.g. for all measures, time points, analyses), and if not, the methods used to decide which results to collect. | 6 |
|  | 10b | List and define all other variables for which data were sought (e.g. participant and intervention characteristics, funding sources). Describe any assumptions made about any missing or unclear information. | 6 |
| Study risk of bias assessment | 11 | Specify the methods used to assess risk of bias in the included studies, including details of the tool(s) used, how many reviewers assessed each study and whether they worked independently, and if applicable, details of automation tools used in the process. | 6 |
| Effect measures | 12 | Specify for each outcome the effect measure(s) (e.g. risk ratio, mean difference) used in the synthesis or presentation of results. | 6 |
| Synthesis methods | 13a | Describe the processes used to decide which studies were eligible for each synthesis (e.g. tabulating the study intervention characteristics and comparing against the planned groups for each synthesis (item #5)). | 6 |
|  | 13b | Describe any methods required to prepare the data for presentation or synthesis, such as handling of missing summary statistics, or data conversions. | 6 |
|  | 13c | Describe any methods used to tabulate or visually display results of individual studies and syntheses. | 6 |
|  | 13d | Describe any methods used to synthesize results and provide a rationale for the choice(s). If meta-analysis was performed, describe the model(s), method(s) to identify the presence and extent of statistical heterogeneity, and software package(s) used. | 6 |
|  | 13e | Describe any methods used to explore possible causes of heterogeneity among study results (e.g. subgroup analysis, meta-regression). | 6 |
|  | 13f | Describe any sensitivity analyses conducted to assess robustness of the synthesized results. | 6 |
| Reporting bias assessment | 14 | Describe any methods used to assess risk of bias due to missing results in a synthesis (arising from reporting biases). | N/A |
| Certainty assessment | 15 | Describe any methods used to assess certainty (or confidence) in the body of evidence for an outcome. | N/A |
| **RESULTS** | | |  |
| Study selection | 16a | Describe the results of the search and selection process, from the number of records identified in the search to the number of studies included in the review, ideally using a flow diagram. | Figure 1 |
|  | 16b | Cite studies that might appear to meet the inclusion criteria, but which were excluded, and explain why they were excluded. | N/A |
| Study characteristics | 17 | Cite each included study and present its characteristics. | Supplementary Tables 2-4 |
| Risk of bias in studies | 18 | Present assessments of risk of bias for each included study. | Supplementary Tables 5-6 |
| Results of individual studies | 19 | For all outcomes, present, for each study: (a) summary statistics for each group (where appropriate) and (b) an effect estimate and its precision (e.g. confidence/credible interval), ideally using structured tables or plots. | 7; Supplementary Tables 2-4 |
| Results of syntheses | 20a | For each synthesis, briefly summarise the characteristics and risk of bias among contributing studies. | 7 |
|  | 20b | Present results of all statistical syntheses conducted. If meta-analysis was done, present for each the summary estimate and its precision (e.g. confidence/credible interval) and measures of statistical heterogeneity. If comparing groups, describe the direction of the effect. | 7-12; Table 2; Table 3; figure 2 |
|  | 20c | Present results of all investigations of possible causes of heterogeneity among study results. | 9 |
|  | 20d | Present results of all sensitivity analyses conducted to assess the robustness of the synthesized results. | 9 |
| Reporting biases | 21 | Present assessments of risk of bias due to missing results (arising from reporting biases) for each synthesis assessed. | 9 |
| Certainty of evidence | 22 | Present assessments of certainty (or confidence) in the body of evidence for each outcome assessed. | N/A |
| **DISCUSSION** | | |  |
| Discussion | 23a | Provide a general interpretation of the results in the context of other evidence. | 9 |
|  | 23b | Discuss any limitations of the evidence included in the review. | 13 |
|  | 23c | Discuss any limitations of the review processes used. | 13 |
|  | 23d | Discuss implications of the results for practice, policy, and future research. | 14 |
| **OTHER INFORMATION** | | |  |
| Registration and protocol | 24a | Provide registration information for the review, including register name and registration number, or state that the review was not registered. | 2 |
|  | 24b | Indicate where the review protocol can be accessed, or state that a protocol was not prepared. | 4-5 |
|  | 24c | Describe and explain any amendments to information provided at registration or in the protocol. | N/A |
| Support | 25 | Describe sources of financial or non-financial support for the review, and the role of the funders or sponsors in the review. | 16 |
| Competing interests | 26 | Declare any competing interests of review authors. | 16 |
| Availability of data, code and other materials | 27 | Report which of the following are publicly available and where they can be found: template data collection forms; data extracted from included studies; data used for all analyses; analytic code; any other materials used in the review. | 16 |

*From:*  Page MJ, McKenzie JE, Bossuyt PM, Boutron I, Hoffmann TC, Mulrow CD, et al. The PRISMA 2020 statement: an updated guideline for reporting systematic reviews. BMJ 2021;372:n71. doi: 10.1136/bmj.n71

For more information, visit: <http://www.prisma-statement.org/>

Reference:

1 Zhang T, Zhu Q, Zhang X, Ding Y, Steinhoff M, Black S, et al. Clinical Characteristics and Direct Medical Cost of Respiratory Syncytial Virus Infection in Children Hospitalized in Suzhou, China. The Pediatric Infectious Disease Journal 2014;33.

2 Arriola CS, Kim L, Langley G, Anderson EJ, Openo K, Martin AM, et al. Estimated Burden of Community-Onset Respiratory Syncytial Virus–Associated Hospitalizations Among Children Aged &lt;2 Years in the United States, 2014–15. Journal of the Pediatric Infectious Diseases Society 2019;9:587-595.

3 Zhou Y, Tong L, Li M, Wang Y, Li L, Yang D, et al. Recurrent Wheezing and Asthma After Respiratory Syncytial Virus Bronchiolitis. Frontiers in Pediatrics 2021;9.

4 Toivonen L, Karppinen S, Schuez-Havupalo L, Teros-Jaakkola T, Mertsola J, Waris M, et al. Respiratory syncytial virus infections in children 0–24 months of age in the community. Journal of Infection 2020;80:69-75.

5 Boonyaratanakornkit J, Englund JA, Magaret AS, Bu Y, Tielsch JM, Khatry SK, et al. Primary and Repeated Respiratory Viral Infections Among Infants in Rural Nepal. Journal of the Pediatric Infectious Diseases Society 2018;9:21-29.

6 MI Pei-ming TM-f, HUANG Jia-yi, WEN Shang-mei, LIU Zhi-wei. Clinical analysis of 842 children with Respiratory Syncytial Virus pneumonia. CHINA MODERN MEDICINE 2019;26:45-48.

7 Okiro EA, Ngama M, Bett A, Nokes DJ. The incidence and clinical burden of respiratory syncytial virus disease identified through hospital outpatient presentations in Kenyan children. PLoS One 2012;7:e52520.

8 McCracken JP, Arvelo W, Ortíz J, Reyes L, Gray J, Estevez A, et al. Comparative epidemiology of human metapneumovirus‐and respiratory syncytial virus‐associated hospitalizations in G uatemala. Influenza and other respiratory viruses 2014;8:414-421.

9 Thwaites R, Buchan S, Fullarton J, Morris C, Grubb E, Rodgers-Gray B, et al. Clinical burden of severe respiratory syncytial virus infection during the first 2 years of life in children born between 2000 and 2011 in Scotland. European Journal of Pediatrics 2020;179:791-799.

10 Svensson C, Berg K, Sigurs N, Trollfors B. Incidence, risk factors and hospital burden in children under five years of age hospitalised with respiratory syncytial virus infections. Acta paediatrica 2015;104:922-926.

11 Kobayashi Y, Togo K, Agosti Y, McLaughlin JM. Epidemiology of respiratory syncytial virus in Japan: A nationwide claims database analysis. Pediatr Int 2022;64:e14957.

12 Chi H, Chang IS, Tsai F-Y, Huang L-M, Shao P-L, Chiu N-C, et al. Epidemiological Study of Hospitalization Associated With Respiratory Syncytial Virus Infection in Taiwanese Children Between 2004 and 2007. Journal of the Formosan Medical Association 2011;110:388-396.

13 Yingying L. Clinical analysis of 365 children with respiratory syncytial virus pneumonia. Guangdong Medical Journal 2015;36:2558-2560.

14 Li-ling ZX-bWC-kLL-jJG-lWL-bSPXJQ. Clinical features and disease burden of acute low respiratory infection caused by respiratory syncytial virus in hospitalizedneonatesand infants. Chin J Evid Based Pediatr 2014;9:45-48.

15 McLaurin KK, Farr AM, Wade SW, Diakun DR, Stewart DL. Respiratory syncytial virus hospitalization outcomes and costs of full-term and preterm infants. Journal of Perinatology 2016;36:990-996.

16 Forbes ML, Hall CB, Jackson A, Masaquel AS, Mahadevia PJ. Comparative costs of hospitalisation among infants at high risk for respiratory syncytial virus lower respiratory tract infection during the first year of life. Journal of Medical Economics 2010;13:136-141.

17 Fergie J, Suh M, Jiang X, Fryzek JP, Gonzales T. Respiratory syncytial virus and all-cause bronchiolitis hospitalizations among preterm infants using the pediatric health information system (PHIS). The Journal of infectious diseases 2022;225:1197-1204.

18 Nguyen-Van-Tam J, Wyffels V, Smulders M, Mazumder D, Tyagi R, Gupta N, et al. Cumulative incidence of post-infection asthma or wheezing among young children clinically diagnosed with respiratory syncytial virus infection in the United States: A retrospective database analysis. Influenza Other Respir Viruses 2020;14:730-738.

19 Yu-chuan FLZY-mW. clinical and epidemiological characteristics of lower resiratory tract syncytial virus infections. Chin J Nosocomio 2013;23:5483-5484+5487.

20 REN Kang-Yi RL, DENG Yu, XIE Xiao-Hong, ZANG Na, XIE Jun, LUO Zheng-Xiu, LUO Jian, FU Zhou, LIU EnMei, LI Qu-Bei. Epidemiological characteristics of respiratory syncytial virus in hospitalized children with acute lower respiratory tract infection in Chongqing, China, from 2013 to 2018: an analysis of 2 066 cases. Chin J Contemp Pediatr 2021;23:67-73.

21 Gen-ming RS-lSX-jSTSWCQ-hXJTJ-mZTZ. Analysis of clinical characteristics and influencing factors of respiratory syncytial virus infection among children under 5 years old in Suzhou. Chin J Dis Control Prev 2021;25:1336-1340+1364.

22 Yu Yun LL, Chen Li-ping. Clinical analysis of 152 cases of neonatal respiratory syncytial virus pneumonia. Jiangxi Medical Journal 2020;55:1685-1686.

23 Buchan SA, Chung H, Karnauchow T, McNally JD, Campitelli MA, Gubbay JB, et al. Characteristics and outcomes of young children hospitalized with laboratory-confirmed influenza or respiratory syncytial virus in Ontario, Canada, 2009–2014. The Pediatric Infectious Disease Journal 2019;38:362-369.

24 Halasa N, Williams J, Faouri S, Shehabi A, Vermund SH, Wang L, et al. Natural history and epidemiology of respiratory syncytial virus infection in the Middle East: Hospital surveillance for children under age two in Jordan. Vaccine 2015;33:6479-6487.

25 Linssen RS, Bem RA, Kapitein B, Rengerink KO, Otten MH, den Hollander B, et al. Burden of respiratory syncytial virus bronchiolitis on the Dutch pediatric intensive care units. European journal of pediatrics 2021;180:3141-3149.

26 Anderson EJ, Carbonell-Estrany X, Blanken M, Lanari M, Sheridan-Pereira M, Rodgers-Gray B, et al. Burden of Severe Respiratory Syncytial Virus Disease Among 33-35 Weeks' Gestational Age Infants Born During Multiple Respiratory Syncytial Virus Seasons. Pediatr Infect Dis J 2017;36:160-167.

27 Zhong Q, Feng H, Lu Q, Liu X, Zhao Q, Du Y, et al. Recurrent wheezing in neonatal pneumonia is associated with combined infection with Respiratory Syncytial Virus and Staphylococcus aureus or Klebsiella pneumoniae. Scientific Reports 2018;8:1-8.

28 Munywoki PK, Ohuma EO, Ngama M, Bauni E, Scott JAG, Nokes DJ. Severe lower respiratory tract infection in early infancy and pneumonia hospitalizations among children, Kenya. Emerging infectious diseases 2013;19:223.

29 Mejias A, Wu B, Tandon N, Chow W, Varma R, Franco E, et al. Risk of childhood wheeze and asthma after respiratory syncytial virus infection in full‐term infants. Pediatric Allergy and Immunology 2020;31:47-56.

30 Romero JR, Stewart DL, Buysman EK, Fernandes AW, Jafri HS, Mahadevia PJ. Serious early childhood wheezing after respiratory syncytial virus lower respiratory tract illness in preterm infants. Clinical therapeutics 2010;32:2422-2432.

31 Brenes-Chacon H, Garcia-Mauriño C, Moore-Clingenpeel M, Mertz S, Ye F, Cohen DM, et al. Age-dependent interactions among clinical characteristics, viral loads and disease severity in young children with respiratory syncytial virus infection. The Pediatric infectious disease journal 2021;40:116-122.

32 Vizcarra-Ugalde S, Rico-Hernández M, Monjarás-Ávila C, Bernal-Silva S, Garrocho-Rangel ME, Ochoa-Pérez UR, et al. Intensive care unit admission and death rates of infants admitted with respiratory syncytial virus lower respiratory tract infection in Mexico. The Pediatric Infectious Disease Journal 2016;35:1199-1203.

33 Demont C, Petrica N, Bardoulat I, Duret S, Watier L, Chosidow A, et al. Economic and disease burden of RSV-associated hospitalizations in young children in France, from 2010 through 2018. BMC Infectious Diseases 2021;21:730.

34 Kramer R, Duclos A, Lina B, Casalegno J-S. Cost and burden of RSV related hospitalisation from 2012 to 2017 in the first year of life in Lyon, France. Vaccine 2018;36:6591-6593.

35 Helfrich AM, Nylund CM, Eberly MD, Eide MB, Stagliano DR. Healthy Late-preterm infants born 33–36+6 weeks gestational age have higher risk for respiratory syncytial virus hospitalization. Early Human Development 2015;91:541-546.

36 Feng Ying LZ, Fu Zhou, L, Luo Jian, L, Liu Enmei Bacteria and viruses in hospitalized infants with community acquired pneumonia. J Clin Pediatr;31:1042-1045.

37 Ling LFWTXCYDLHG. The risk factors influencing pediatric asthmatic bronchitis. JOURNAL OF GUANGXI MEDICAL UNIVERSITY 2017;34:1486-1489.

38 Hospital，Jincheng，ShanxiSu SHTJPs. Analysis of distribution characteristics of common pathogens in infants with wheezing. Primary Medical Forum; 23: 1335-1337.

39 YANG Sai WG, RONG Ji-ayan , LIU Xiangteng, WANG BingJie. Risk factors for infants and young children with wheezing in Zhongshan city J Clin Pediatr; 32: 126-130.

40 ZHONG Qiu-lan YJ-c, ZHU Xu-liang. Analysis of high risk factors for pediatric wheezing. Chin J Mod Drug Appl 2016;10:10-11.

41 Zhi-yong LP-qANZPZ. Analysis of the correlation between asthmatic and respiratory viral infections in infants. CHINA MODERN MEDICINE 2019;26:107-109.

42 Wang Kun ZY, Wang Tie-yan, Chen Jing, Zhao Wei. Analysis of risk factors for asthmatic bronchitis in children. Modern Medicine and Health Research 2022;16:106-109.

43 Blanken MO, Korsten K, Achten NB, Tamminga S, Nibbelke EE, Sanders EA, et al. Population‐attributable risk of risk factors for recurrent wheezing in moderate preterm infants during the first year of life. Paediatric and perinatal epidemiology 2016;30:376-385.

44 Yongsheng ZJZYG. Logistic Ｒegression Analysis for Detecting the Ｒisk Factors for Infants Ｒecurrent Wheezing. J Med Theor ＆ Prac 2018;31:21-23.

45 Escobar GJ, Ragins A, Li SX, Prager L, Masaquel AS, Kipnis P. Recurrent Wheezing in the Third Year of Life Among Children Born at 32 Weeks' Gestation or Later: Relationship to Laboratory-Confirmed, Medically Attended Infection With Respiratory Syncytial Virus During the First Year of Life. Archives of Pediatrics & Adolescent Medicine 2010;164:915-922.

46 Simões EAF, Dani V, Potdar V, Crow R, Satav S, Chadha MS, et al. Mortality From Respiratory Syncytial Virus in Children Under 2 Years of Age: A Prospective Community Cohort Study in Rural Maharashtra, India. Clinical Infectious Diseases 2021;73:S193-S202.

47 Jalink MB, Langley JM, Dodds L, Andreou P. Severe respiratory syncytial virus infection in preterm infants and later onset of asthma. The Pediatric Infectious Disease Journal 2019;38:1121-1125.
